# Efficacy and safety of topically applied therapeutic ammonia oxidising bacteria in adults with mild-to-moderate atopic dermatitis and moderate-to-severe pruritus: a randomised, double-blind, placebo-controlled, dose-ranging, phase 2b trial

**DOI:** 10.1016/j.eclinm.2023.102002

**Published:** 2023-05-16

**Authors:** Jonathan I. Silverberg, Peter A. Lio, Eric L. Simpson, Connie Li, Daniel R. Brownell, Ioannis Gryllos, Judith Ng-Cashin, Todd Krueger, Victoria R. Swaidan, Robin L. Bliss, Hyun D. Kim

**Affiliations:** a George Washington University School of Medicine and Health Sciences, Washington, DC, USA; b Northwestern University Feinberg School of Medicine, Chicago, IL, USA; c Department of Dermatology, Oregon Health & Science University, Portland, OR, USA; d AOBiome Therapeutics, Inc., Cambridge, MA, USA; e Biorasi, LLC, Aventura, FL, USA; f Veristat, LLC, Southborough, MA, USA

**Keywords:** Atopic dermatitis, Pruritus, Ammonia-oxidising bacteria, Live biotherapeutic product, Anti-inflammatory

## Abstract

**Background:**

Topical anti-inflammatory therapy is a cornerstone of treatment for atopic dermatitis (AD). However, many unmet needs remain with existing therapies. B244 is a live topical biotherapeutic being tested for the reduction of pruritus and improvement of eczema signs in patients with AD. We aimed to assess the safety and efficacy of B244, compared to vehicle, for patients with mild-to-moderate AD and moderate-to-severe pruritus.

**Methods:**

In this randomised, placebo-controlled, double-blind phase 2b trial, adults aged 18–65 years with mild-to-moderate AD and moderate-to-severe pruritus were enrolled across 56 sites in the USA. Patients were randomised 1:1:1 into a low-dose (optical density at 600 nm [OD] 5.0), high-dose (OD 20.0), or vehicle group for the 4-week treatment period and a 4 week follow-up period. Patients were instructed to apply the topical spray twice daily throughout the treatment period. Randomisation was centrally based (random alternating blocks of 6 and 3) and stratified by site. All participants, investigators, and those assessing outcomes were blinded to the treatment group assignments. The primary endpoint was the mean change in pruritus as measured by the Worst Itch Numeric Rating Scale (WI-NRS) at 4 weeks. Safety was tracked throughout the study. Primary efficacy analyses included the modified intent-to-treat (mITT) population, encompassing those who received at least one dose of study drug and attended at least one post-baseline visit. The safety population included all participants who received at least one does of study drug. This study is registered with ClinicalTrials.gov, NCT04490109.

**Findings:**

Between June 4, 2020 and October 22, 2021, 547 eligible patients were enrolled. All study endpoints were meaningfully improved with B244 compared to vehicle. The WI-NRS score was reduced by 34% (−2.8 B244 vs −2.1 placebo, p = 0.014 and p = 0.015 for OD 20.0 and OD 5.0), from a baseline score of >8. B244 was well tolerated with no serious adverse events (SAEs); treatment-emergent adverse events (TEAEs) and treatment related TEAEs were low in incidence, mild in severity, and transient. 33 (18%) of 180 patients given B244 OD 5.0, 29 (16%) of 180 patients given B244 OD 20.0, and 17 (9%) of 186 patients given placebo reported treatment-emergent adverse events; headache was the most frequent (3%, 2%, and 1%, respectively).

**Interpretation:**

B244 was well tolerated and demonstrated improved efficacy compared to vehicle in all primary, secondary, and exploratory endpoints and should be further developed as a novel, natural, fast-acting topical spray treatment option for AD and associated pruritus.

**Funding:**

AOBiome Therapeutics.

**Research in context**

**Evidence before this study**

Prior Phase 1 and Phase 2a studies of B244 for the treatment of atopic dermatitis (AD) showed favorable data for safety and efficacy endpoints. Specifically, the Phase 2a study showed significant improvements in pruritus. Prior to the start of this study, we searched clinicaltrials.gov for randomized, blinded, clinical trial using keywords “atopic dermatitis” and “pruritus”. We searched PubMed for high-quality publications on the topics of “atopic dermatitis,” “eczema,” “itch,” and “live biotherapeutic.” Additionally, research of the current treatment guidelines suggested that pruritus was a significant unmet need not well-addressed by the available treatments. We encountered safety concerns with topical corticosteroids, Janus kinase (JAK) inhibitors, and biologics, as well as irritation concerns with some topicals. Our toxicology studies supported an increase in dose for this study, which allowed for a 2.5 and 10-fold increase as compared to our previous studies in AD. We hypothesized that an increased dose would drive separation between treatment and control groups, without safety risks. The study was powered to overcome the placebo effect often observed in atopic dermatitis trials testing topicals, leading to the largest randomised clinical trial (RCT) to date for a topical live biotherapeutic.

**Added value of this study**

Our results demonstrate significant improvements in clinical signs and symptoms metrics at both the low (Optical Density (OD) 5.0) and high (OD 20.0) dose levels. Importantly, the primary endpoint in itch relief (mean change in Worst Itch Numeric Rating Scale (WI-NRS)) was meaningfully improved, along with secondary/exploratory endpoints of mean change in Investigator Global Assessment (IGA), mean change in Eczema Area and Severity Index (EASI), and the clinically meaningful itch response rate (≥4 point WI-NRS improvement from baseline), IGA success, and EASI-75. Itch relief appeared fast acting, as observed by separation of active and vehicle treatment groups after 2 days of treatment. After discontinuing use, follow-up assessments at 8 weeks maintained significant improvements, after being off treatment for 4 weeks. Our study showed that B244 treatment is very safe, with few adverse events in any treatment or vehicle groups. In agreement with previous RCTs, B244 maintained its track record of safety in human use.

**Implications of all the available evidence**

Itch (pruritus) is the most common and burdensome symptom among patients suffering from atopic dermatitis. For some therapeutic options that improve AD clinical signs and itch symptoms, itch relief can take as long as 16 weeks. In our study, B244 demonstrates significant itch reduction within 1 week, with separation between treatment and control groups as early as day 2. By demonstrating significant efficacy and safety, our Phase 2b study suggests that B244 can be a successful treatment option for patients with mild-to-moderate AD and moderate-to-severe itch.

## Introduction

Atopic dermatitis (AD) is a chronic inflammatory skin disease that affects nearly 20% of children and 2–10% of adults.^3^ Pruritus is a universal symptom of AD, and its management remains a challenge for physicians. Pruritus, defined as an unpleasant sensation that produces the urge to scratch^1^ is the most common and burdensome symptoms of AD.^2^ AD is often described as “the itch that rashes” because itch often precedes the appearance of skin lesions. The itch cycle exacerbates damage to the epidermal barrier leading to water loss and dryness, and creates a hospitable environment for skin pathogens leading to infections and worsening of inflammation and symptoms. AD exhibits heterogeneous clinical phenotypes, including different combinations of itch and lesional severity. This is supported by a variety of clinical AD phenotypes as defined by severity of itch and AD lesions, with patients exhibiting severe itch and mild to moderate lesions (21.3–29.1%) and mild to moderate itch and mild to moderate lesions (59.4–62.3%) totaling 80.7–91.4% of the total AD phenotypes.^4^

AD pathogenesis is associated with immune dysregulation of the type 2 inflammatory response, disruption of the skin barrier function, and dysbiosis resulting from a loss of microbial diversity due to an overabundance of *Staphylococcus* bacterial genus.^5^, ^6^, ^7^ *Staphylococcus aureus* is consistently found in eczematous skin lesions in patients with AD. Correlation between the severity of the disease and presence of *Staphylococcus aureus* is well established and was shown that presence of bacteria is an important factor in skin aggravation that leads to loss of microbial diversity in AD flares.^7^^,^^8^ Most of the current topical therapies have anti-inflammatory mechanism of action. Antiseptic and antipruritic therapies to reduce epidermal-barrier dysfunction, immune dysregulation, and infection are used in clinical practice.^2^

B244 consists of a purified strain of *Nitrosomonas eutropha*, an ammonia oxidising bacteria (AOB), originally isolated from soil samples. AOB are essential for the initial step in environmental nitrification processes, specifically the oxidation of ammonia (NH_3_) to nitric oxide (NO) and nitrite (NO_2_-). *Nitrosomonas* are Gram-negative chemolithoautotrophic betaproteobacteria that obtain energy solely from NH_3_ oxidation, while fixing carbon dioxide (CO_2_) for their carbon needs.^9^ B244 may reduce survival of pathogenic bacteria including *Staphylococcus aureus*, which is implicated in disruption of skin-barrier function and AD exacerbation. B244 also reduced Th2-associated cytokines IL-4, IL-5, and IL-13 *in vitro* which were implicated in AD pathology.^10^ The unique metabolic and antimicrobial activity of *Nitrosomonas*, in combination with their lack of virulence render these bacteria as attractive candidates for topical delivery of nitric oxide and nitrite, known to be anti-inflammatory and anti-microbial, respectively, to improve clinical signs and symptoms of AD.

The study reported herein was a dose escalation follow-on Phase 2b study with 2.5-fold (optical density (OD) 5.0) and 10-fold (OD 20.0) higher doses used compared to two earlier studies. First was an open-label, multicentre, Phase 1b study of B244 delivered as a topical spray to assess safety in 28 pediatric patients aged 2–17 years with mild to moderate AD (NCT03775434). Results suggest that B244 treatment (twice daily topical spray) is safe, well-tolerated, and efficacious in the treatment of AD in pediatric patients over 28-days of treatment. The second study was a prospective, vehicle controlled, double blind, multicentre, randomised Phase 2a trial of twice daily B244 versus vehicle application for 28 days in 122 adults with mild-moderate AD (NCT03235024). Results demonstrated safety, tolerability, and an efficacy signal for itch.

## Methods

### Study design and participants

This was a double-blind, randomised, placebo-controlled, Phase 2b study conducted at 56 study centres in the USA. The total duration of the study for each patient was approximately 11 weeks: 1-week screening period (−3 to −2 weeks), 2-week washout period before the baseline (−2 weeks to −1 day), twice-a-day application (10 pumps per application, 0.14 ml per pump) of topical spray study drug (B244 1 × 10^10^ cells/ml [OD 5.0], B244 4 × 10^10^ cells/ml [OD 20.0], or vehicle for 4 weeks (baseline [Day 0] to Week 4), and 4-week follow-up (Week 4–Week 8). The vehicle is a sodium phosphate-based buffer solution, and does not interact with the active ingredient. B244 and matching vehicle were filled in identical 30 ml white topical spray bottles, packaged, and labeled.

Eligible patients were adults (aged 18–65 years) with AD for greater than 12 months consistent with a diagnosis of AD, as defined by the 2014 American Academy of Dermatology (AAD) Guidelines of Care for the Management of AD,^5^ which involved 10%–40% body surface area (BSA), static investigator global assessment (IGA) of 2–3, and a score of ≥7 points on the numerical rating scale (NRS) for itch.^11^^,^^12^ The full list of inclusion and exclusion criteria are described in the Appendix.

The study was conducted in accordance with the ethical principles that have their origins in the Declaration of Helsinki and with Good Clinical Practice (GCP) guidelines as denoted in the International Council for Harmonization (ICH) E6 requirements. Study protocols and documents were reviewed and approved by Advarra, an institutional review board (IRB). All patients were provided a written informed consent form to participate in the study prior to being screened. The CONSORT guidelines were utilized in the reporting of these study results.

Throughout the study, there were a small number of deviations from protocol. For the safety population, there were 5 (3%), 12 (7%), and 6 (3%) patients in the OD 5.0, OD 20.0, and vehicle, respectively, with at least one major protocol deviation/violation (PD/PV), of which the most frequent was use of a prohibited concomitant medication or rescue medication. All secondary and exploratory outcomes are reported here, with the exception of the average itch—numerical rating scale (AI-NRS), as these data (not shown) were deemed to be redundant with the WI-NRS data reported. All outcomes described were pre-specified, no post-hoc analyses are reported.

### Randomisation and masking

Patients were randomly assigned (1:1:1) in this double blind, parallel design study to receive B244 OD 5.0, B244 OD 20.0, or vehicle for twice-daily use. Randomisation was centrally based (random alternating blocks of 6 and 3), stratified by site, and performed using IBM Clinical Development EDC/RTSM. AOBiome Therapeutics and contract research organization personnel involved with study conduct, patients, study investigators, and study centre personnel remained blinded to the treatment assignment throughout the study. Each patient received a randomisation number at the time of randomisation, which was used to identify the study medication kit assigned to the patient and indicate the treatment to be administered to that patient. The dispensing containers of B244 OD 5.0, B244 OD 20.0, and vehicle were identical in appearance to maintain blinding.

### Procedures

All patients attended a screening visit no more than 21 days prior to baseline (Day 0) for 1 week and entered a 2-week washout period (washout of excluded medications listed in the Appendix; allowed to use emollients, oral H1 antihistamines, and permitted rescue medications) upon meeting all eligibility criteria. Upon randomisation at baseline, patients received study drug (B244 OD 5.0, B244 OD 20.0, or vehicle) and were instructed to apply 10 pumps of the study drug to all affected areas twice daily (approximately 12 h apart) for 28 consecutive days. Patients were instructed to apply 10 pumps per application, covering all affected areas of their skin lesions, starting with the most-affected areas. Patients were allowed to use their usual choice of bland emollients throughout the study. Certain rescue medication was permitted upon site investigator evaluation on patient condition as described in the Appendix. Patients were required to return to the study centre at baseline, Week 2, and Week 4 for visit assessments. At home, patients were instructed to enter daily eCOA/ePRO diaries for itch, local tolerability, study drug administration, and rescue medication use. All patients were asked to attend a Week 8 follow-up visit 4 weeks after the last dose of the study drug.

Worst itch in the past 24 h was assessed using a numeric rating scale (WI-NRS) and completed once daily (between 5 pm and midnight local time) by patients from screening to Week 8 (range: 0–10). Patient Oriented Eczema Measure (POEM)^13^ and 5-D Pruritus Scale^14^ were reported by patients at site visits at screening, baseline, Week 2, Week 4, and Week 8. IGA and EASI^15^ were assessed by investigators at screening, baseline, Week 2, Week 4, and Week 8. Safety was assessed at the study visits at screening, baseline, Week 2, Week 4, and Week 8 and continuous assessment of adverse events reported throughout the study duration. Patient-reported local tolerability assessed the following symptoms daily by using electronic clinical outcome assessments/electronic patient reported outcomes (eCOA/ePRO) at home (between 5 pm and midnight local time) during the first 7 days of treatment to determine change from the previous day (see Appendix): skin redness and/or color change, itching, burning and/or stinging, pain and/or tenderness, and new or changing rash according to the following scale for each category: none (0), mild (1), moderate (2), and severe (3). By request of the FDA, tolerability was monitored for the first 7 days only. Once this tolerability was established, close monitoring was determined to no longer be needed.

### Outcomes

The primary endpoint was the mean change in WI-NRS from baseline to Week 4. Secondary and exploratory endpoints were the proportion of patients with ≥4 point improvement in WI-NRS from baseline to Week 4, mean change in WI-NRS from baseline to Week 2, proportion of patients with ≥4 point improvement in WI-NRS from baseline to Week 2, mean change in POEM from baseline to Week 4, mean change in 5-D Pruritus Scale from baseline to Week 4, mean change in IGA from baseline to Week 4, mean change in EASI from baseline to Week 4, proportion of patients with IGA of Clear or Almost Clear and ≥2 point improvement from baseline to Week 4, proportion of patients with IGA of Clear or Almost Clear at Week 4, proportion of patients with ≥75% improvement in EASI (EASI-75) from baseline to Week 4, and proportion of patients with ≥90% improvement in EASI (EASI-90) from baseline to Week 4 (see Appendix).

Safety and tolerability endpoints included incidence of treatment-emergent adverse events (TEAEs) and serious adverse events (SAEs), changes in vital signs and clinical laboratory parameters following study drug exposure, and changes in local skin tolerability following application of study drug. All TEAEs recorded during the study were coded according to Medical Dictionary for Regulatory Activities (MedDRA) version 23.0.

### Statistical analysis

The study was designed to complete 160 evaluable patients per group to achieve at least 80% power to detect a pairwise difference of 0.65 in the primary endpoint of mean WI-NRS change from baseline to Week 4 between one of two active doses of B244 and vehicle control when assuming a standard deviation of 2.5 and applying a Dunnett Testing Method at a one-sided familywise error rate of 0.10.

Primary efficacy analysis was conducted in the modified intent-to-treat (mITT) population, which included all randomised patients who applied at least 1 dose of the study drug and had at least one post-baseline on-site visit. All data presented are from the mITT population unless otherwise included as supportive analysis from the per-protocol (PP) population, which included all patients in the mITT population without any major protocol deviations impacting efficacy assessments, completed the Week 4 visit, and applied at least 50% of the study drug. Safety analysis was conducted in the safety population, which included all patients who applied at least 1 dose of study drug.

The primary endpoint (mean change in WI-NRS from baseline to Week 4) was analysed using analysis of covariance (ANCOVA) models with treatment group and baseline weekly average WI-NRS as explanatory variables. Hypothesis testing was done using a Dunnett Testing Method, applying pairwise comparisons of each respective B244 dose group to vehicle using a one-sided familywise error rate of 0.10. The treatment effect was estimated via least squares means (LSM) using vehicle as reference and adjusted for multiplicity according to the Dunnett Testing Method and presented with one-sided 90% confidence interval (CI) to determine if B244 is superior to vehicle. The difference in treatment groups in change from baseline values at post-baseline timepoints were also analysed using a mixed model with repeated measures (MMRM). Other secondary and exploratory endpoints (continuous measures of WI-NRS, IGA, EASI, POEM, 5-D Pruritus Scale) were analysed using descriptive statistics or a logistic regression model (responder status) at each respective timepoint.

The primary analysis included data for all patients in the respective population (mITT or PP). Sub-analysis was conducted and excluded data collected on/after the date of first on-treatment rescue medication use through the end of the study. For the WI-NRS endpoint, this excluded data from week of discontinuation/first on-treatment rescue medication use through the end of the study. Non-responder imputation (NRI) analysis was also conducted. Patients who discontinued treatment early or who took on-treatment rescue medications were imputed as a non-responder for assessments that occurred on/after the date of discontinuation/on-treatment rescue medication use through the end of the study. For the WI-NRS endpoint, this excluded data from week of discontinuation/first on-treatment rescue medication use through the end of the study. Data from patients who discontinued early were imputed for NRI analysis if they had at least one TEAE leading to discontinuation. Worst case analysis was also performed where all values on/after the date of first on-treatment rescue medication use or all values missing due to patient early discontinuation was replaced with the worst value observed (i.e., Worst Observation Carried Forward or WOCF) for each patient between first post-baseline assessment and first use of on-treatment rescue medication. ITT analysis included all patients who were randomised, regardless of exposure to treatment where patients missing Week 4 values had their measurement imputed to be the Last Observation Carried Forward (LOCF) value on treatment or baseline, whichever was later.

This study was registered with ClinicalTrials.gov, number NCT04490109. All statistical analyses were performed using SAS version 9.4.

### Role of the funding source

The funder of the study had a role in study design, data collection, data analysis, data interpretation, and writing of the report. JS, CL, VS, RB, and HK accessed and verified the data. All authors provided approval for the submission of the manuscript for publication.

## Results

**Fig. 1:**
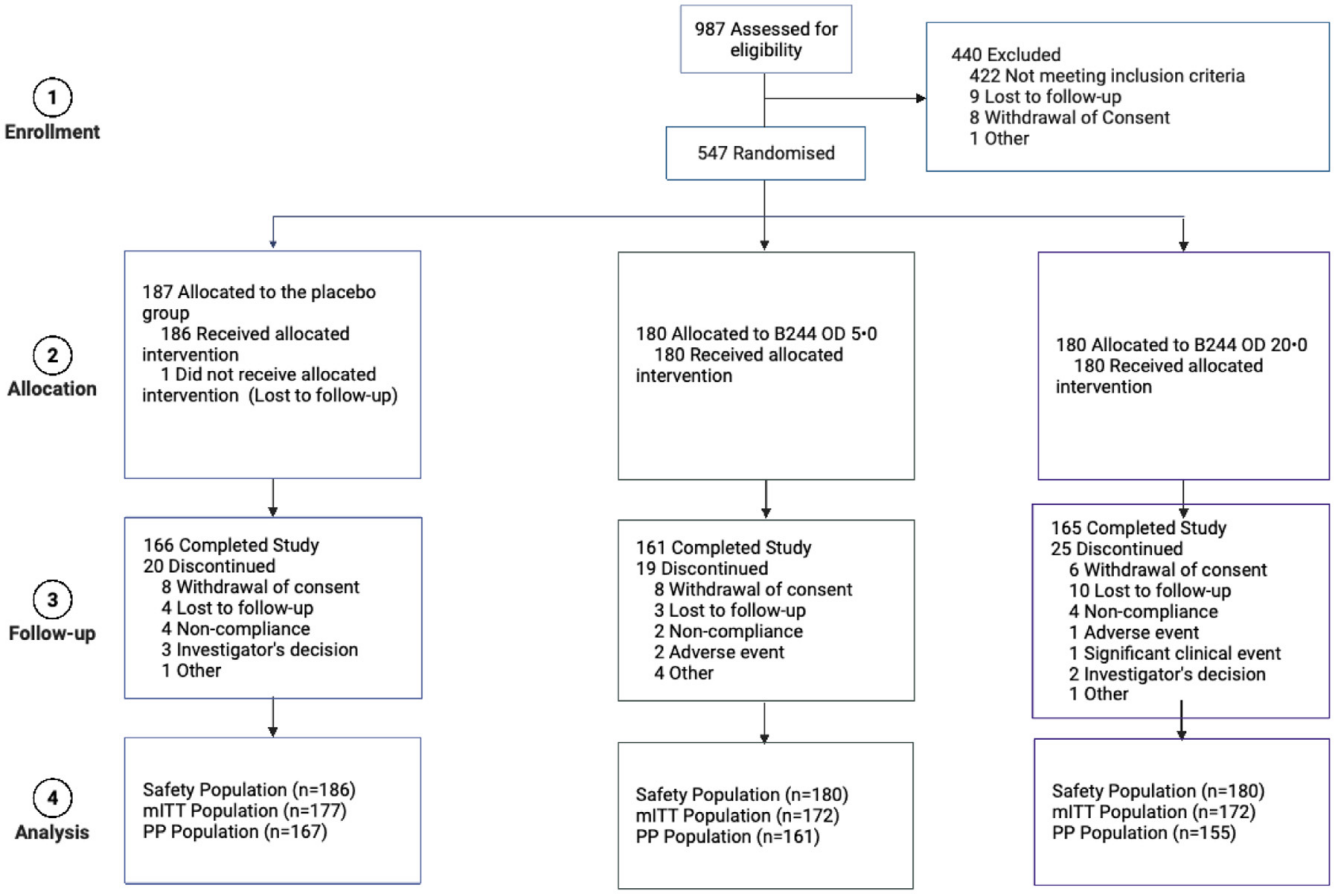
Trial Profile. PP population includes all patients in the mITT population without any major protocol deviations that may have an impact on the efficacy assessments, who complete their Week 4 visit, and who administer at least 50% of investigational product (IP). mITT, modified intent-to-treat population. PP, Per protocol population. Figure created with BioRender.

Between June 4, 2020, and October 22, 2021, 987 patients were screened for eligibility, of whom 547 adults were randomly assigned to receive B244 OD 5.0 [1 × 10^10^ cells/mL] (n = 180), B244 OD 20.0 [4 × 10^10^ cells/mL] (n = 180), or vehicle (n = 187; Fig. 1). 546 patients (safety population; 180 in the B244 OD 5.0 group, 180 in the B244 OD 20.0 group, 186 in the vehicle group) received one or more doses of study treatment, of whom 482 patients (88%) [161 (89%) patients in the B244 OD 5.0 group, 155 (86%) patients in the B244 OD 20.0 group, and 166 (89%) patients in the vehicle group] completed 4 weeks of treatment and 4 weeks of follow-up. Efficacy analysis was performed on 521 patients in the mITT population [172 (96%) patients in the B244 OD 5.0 group, 172 (96%) patients in the B244 OD 20.0 group, and 177 (95%) patients in the vehicle group] and the PP population [161 (89%) patients in the B244 OD 5.0 group, 155 (86%) patients in the B244 OD 20.0 group, and 167 (90%) patients in the vehicle group]. Pooled B244 group included both B244 OD 5.0 and OD 20.0 groups. Two (1%) of 180 patients in the B244 OD 5.0 group, and none from the other groups discontinued study or treatment because of COVID-19 during the double-blind treatment period.

Demographics and baseline characteristics were balanced between the treatment groups in the mITT population (Table 1), including baseline WI-NRS score, IGA score, EASI score, percentage of body surface area affected, rescue medication use, and the proportion of patients with mild and moderate IGA, and were similar in the PP and safety populations. Mean WI-NRS score at baseline was nearly 8.2. Proportion of patients with mild (IGA = 2) and moderate (IGA = 3) AD at baseline ranged 27–31% and 68–73%, respectively. Rescue medication use during the baseline period (2 weeks prior to the baseline visit) was balanced between treatment groups with 31/180 (17%) patients in the B244 OD 5.0 group, 27/180 (15%) patients in the B244 OD 20.0 group, and 35/186 (19%) patients in the vehicle group. Overall rescue medication use while on-treatment (Weeks 1–4) was low for all groups with 10/180 (6%) of patients in the OD 5.0 group, 16/180 (9%) patients in the OD 20.0 group, and 11/186 (6%) in the vehicle group and remained low during the follow-up period (Weeks 5–8) with 11/180 (7%) of patients in the OD 5.0 group, 8/180 (5%) patients in the OD 20.0 group, and 8/186 (5%) in the vehicle group. There was good compliance with the topical application of the investigational product (IP) across all treatment groups but trended higher for the B244 treatment groups (90% for B244 OD 5.0 and 93% for B244 OD 20.0) relative to the vehicle group (85%).

**Table 1:** Demographics and baseline characteristics of the mITT population.

	Statistic	B244 OD 5.0 (N = 172)	B244 OD 20.0 (N = 172)	Pooled B244 (N = 344)	Vehicle (N = 177)
Age					
18–40 years	n (%)	78 (45)	80 (47)	158 (46)	74 (42)
41–54 years	n (%)	57 (33)	52 (30)	109 (32)	64 (36)
55–65 years	n (%)	37 (22)	40 (23)	77 (22)	39 (22)
Age	n	172	172	344	177
	Mean (SD)	42.2 (12.93)	42.1 (13.49)	42.1 (13.19)	42.1 (13.15)
	Median	43.0	42.5	43.0	43.0
	Min, Max	18, 65	18, 65	18, 65	18, 64
Sex					
Male	n (%)	54 (31)	61 (36)	115 (33)	55 (31)
Female	n (%)	118 (69)	111 (65)	229 (67)	122 (69)
Ethnicity					
Hispanic or Latino	n (%)	87 (51)	88 (51)	175 (51)	85 (48)
Not Hispanic or Latino	n (%)	85 (49)	84 (49)	169 (49)	92 (52)
Race					
American Indian or Alaskan Native	n (%)	0	0	0	0
Asian	n (%)	19 (11)	18 (11)	37 (11)	21 (12)
Black or African American	n (%)	36 (21)	41 (24)	77 (22)	39 (22)
Native Hawaiian or Other Pacific Islander	n (%)	0	0	0	1 (1)
White	n (%)	113 (66)	111 (65)	224 (65)	114 (64)
Other	n (%)	4 (2)	2 (1)	6 (2)	2 (1)
Height (cm)	n	172	172	344	177
	Mean (SD)	166.100 (9.706)	165.608 (9.923)	165.854 (9.804)	166.102 (8.836)
	Median	165.000	165.100	165.100	165.100
	Min, Max	135.00, 190.50	125.27, 187.90	125.27, 190.50	147.00, 191.00
Weight (kg)	n	172	172	344	177
	Mean (SD)	84.144 (22.104)	81.351 (19.603)	82.747 (20.907)	81.908 (20.653)
	Median	80.300	80.800	80.650	78.100
	Min, Max	45.57, 181.00	36.70, 165.00	36.70, 181.00	44.90, 156.49
BMI (kg/m^2^)	n	172	172	344	177
	Mean (SD)	30.576 (8.112)	29.610 (6.577)	30.093 (7.390)	29.729 (7.403)
	Median	28.990	28.510	28.705	28.290
	Min, Max	16.69, 72.40	16.99, 55.49	16.69, 72.40	18.55, 55.68
BSA (%)					
10–20%	n (%)	108 (63)	113 (66)	221 (64)	118 (67)
>20–30%	n (%)	53 (31)	52 (30)	105 (31)	52 (29)
>30–40%	n (%)	11 (6)	7 (4)	18 (5)	7 (4)
BSA (%)	n	172	172	344	177
	Mean (SD)	18.82 (7.247)	18.02 (6.069)	18.42 (6.686)	18.08 (6.600)
	Median	19.00	17.00	18.00	17.00
	Min, Max	10.0, 56.0	10.0, 39.0	10.0, 56.0	4.0, 39.0
Smoking status					
Yes	n (%)	30 (17)	28 (16)	58 (17)	29 (16)
No	n (%)	142 (83)	144 (84)	286 (83)	148 (84)
Rescue medication^a^					
Yes	n (%)	30 (17)	27 (16)	57 (17)	33 (19)
No	n (%)	142 (83)	145 (84)	287 (83)	144 (81)
Rescue medication days^a^	n	30	27	57	33
	Mean (SD)	1.4 (0.72)	1.3 (0.78)	1.4 (0.74)	1.7 (1.85)
	Median	1.0	1.0	1.0	1.0
	Min, Max	1, 4	1, 4	1, 4	1, 9
WI-NRS	n	172	172	344	177
	Mean (SD)	8.21 (0.913)	8.26 (1.004)	8.23 (0.959)	8.26 (1.094)
	Median	8.10	8.20	8.20	8.30
	Min, Max	6.2, 10.0	6.0, 10.0	6.0, 10.0	2.7, 10.0
5-D pruritus scale total score	n	172	172	344	177
	Mean (SD)	17.0 (2.59)	16.6 (2.86)	16.8 (2.73)	17.0 (2.89)
	Median	17.0	16.0	16.0	17.0
	Min, Max	11, 25	10, 24	10, 25	11, 25
EASI					
≤10	n (%)	125 (73)	116 (67)	241 (70)	122 (69)
>10	n (%)	47 (27)	56 (33)	103 (30)	55 (31)
EASI total score	n	172	172	344	177
	Mean (SD)	9.23 (5.463)	8.98 (4.321)	9.11 (4.920)	8.92 (4.560)
	Median	7.75	7.90	7.80	7.70
	Min, Max	1.0, 40.8	2.0, 25.2	1.0, 40.8	1.5, 24.4
IGA					
Mild	n (%)	53 (31)	53 (31)	106 (31)	48 (27)
Moderate	n (%)	117 (68)	119 (69)	236 (69)	129 (73)
Severe	n (%)	2 (1)	0	2 (1)	0
IGA	n	172	172	344	177
	Mean (SD)	2.7 (0.48)	2.7 (0.46)	2.7 (0.47)	2.7 (0.45)
	Median	3.0	3.0	3.0	3.0
	Min, Max	2, 4	2, 3	2, 4	2, 3
POEM total score	n	172	172	344	177
	Mean (SD)	16.6 (5.03)	15.7 (4.88)	16.1 (4.97)	16.1 (5.49)
	Median	16.0	16.0	16.0	16.0
	Min, Max	7, 28	4, 28	4, 28	6, 28

OD, Optical density. BMI, Body mass index. BSA, Body surface area. WI-NRS, Worst itch numerical rating scale. EASI, Eczema area and severity index. IGA, Investigator global assessment. POEM, Patient oriented eczema measure.

a Baseline rescue medication days is the number of rescue medication days recorded during the 2 weeks (Day −14 to Day −1) prior to the baseline visit.

**Fig. 2:**
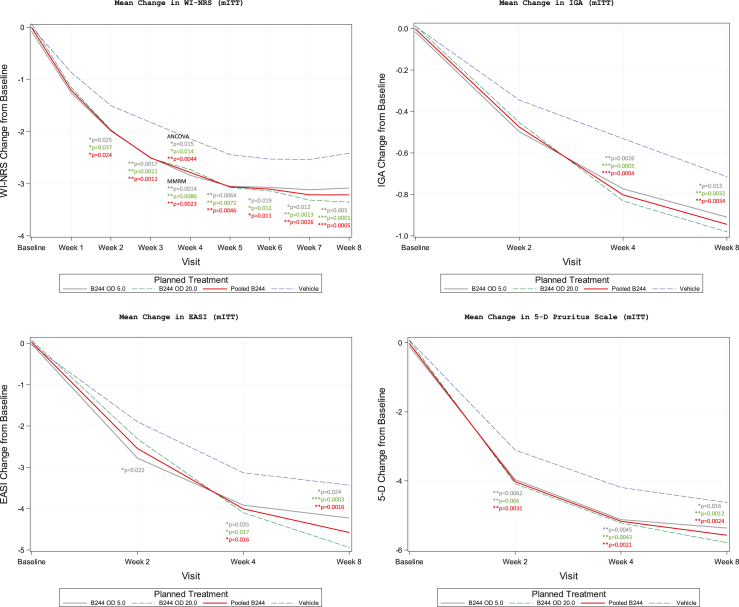
Least-squares mean change from baseline for itch and Atopic Dermatitis clinical signs in the modified intent-to-treat population. AD, Atopic dermatitis. OD, Optical density. WI-NRS, Worst itch numerical rating scale. IGA, Investigator global assessment. EASI, Eczema area and severity index.

Patients in the B244 treatment groups showed significantly larger reductions in their WI-NRS score from baseline to Week 4 compared to patients in the vehicle group. The primary endpoint of mean change in WI-NRS from baseline to Week 4, as analysed by ANCOVA, is summarized in Table 2 for the mITT population and was similar in the PP population. Treatment with either dose of B244 (OD 5.0 or OD 20.0) demonstrated a significant treatment effect versus vehicle at Week 4. The mean reduction from baseline of 34% was equivalent for the OD 5.0 and the OD 20.0 treatment groups with a LSM change from baseline of −2.8 (95% CI −3.2, −2.4). Consequently, pooled B244 showed similar results with LSM = −2.8 (95% CI −3.1, −2.5). The decrease from the mean was less for the vehicle group with LSM = −2.1 (95% CI 2.5, −1.8). The LSM difference was −0.7 [p = 0.015] for OD 5.0, −0.7 [p = 0.014] for OD 20.0, and −0.7 [p = 0.0044] for pooled B244 with no LSM difference (0.0 [p = 0.49]) between OD 5.0 and OD 20.0 (Fig. 2). The results for the sub-analysis, ITT analysis, and worst-case analysis were consistent with that of the primary analysis of mean change in WI-NRS score from baseline to Week 4.

**Table 2:** Efficacy outcomes, baseline to week 4 of the mITT, PP, or ITT populations.

Mean change in WI-NRS from baseline to Week 4—ANCOVA (modified intent-to-treat population)						
Visit	Statistic type	Statistic	B244 OD 5.0 (N = 172)	B244 OD 20.0 (N = 172)	Pooled B244 (N = 344)	Vehicle (N = 177)
Week 4 CFB	ANCOVA	n	166	165	331	174
		LSM (SE)	−2.8 (0.184)	−2.8 (0.184)	−2.8 (0.130)	−2.1 (0.180)
		95% CI	(−3.2, −2.4)	(−3.2, −2.4)	(−3.1, −2.5)	(−2.5, −1.8)
	v. Vehicle	LSM difference (SE)	−0.7 (0.257)	−0.7 (0.257)	−0.7 (0.222)	N/A
		90% UCL adjusted	−0.3	−0.3	−0.4	N/A
		p-value	0.015	0.014	0.0044	N/A
	20.0 v 5.0	LSM difference (SE)	N/A	0.0 (0.266)	N/A	N/A
		90% UCL adjusted	N/A	0.3	N/A	N/A
		p-value	N/A	0.49	N/A	N/A

Mean change in WI-NRS from baseline to week 4—Sub-analysis (modified intent-to-treat population)						
Visit	Statistic type	Statistic	B244 OD 5.0 (N = 172)	B244 OD 20.0 (N = 172)	Pooled B244 (N = 344)	Vehicle (N = 177)
Week 4 CFB	MMRM	n	158	150	308	164
		LSM (SE)	−2.9 (0.141)	−2.8 (0.143)	−2.9 (0.125)	−2.2 (0.174)
		95% CI	(−1.6, −1.1)	(−1.4, −0.9)	(−1.5, −1.0)	(−1.2, −0.5)
	v. Vehicle	LSM difference (SE)	−0.7 (0.224)	−0.6 (0.225)	−0.7 (0.214)	N/A
		95% CI	(−1.2, −0.3)	(−1.0, −0.2)	(−1.1, −0.3)	N/A
		p-value	0.0009	0.0072	0.0016	N/A
	20.0 v. 5.0	LSM difference (SE)	N/A	−0.4 (0.263)	N/A	N/A
		95% CI	N/A	(−0.9, 0.1)	N/A	N/A
		p-value	N/A	0.15	N/A	N/A

Mean change in WI-NRS from baseline to Week 4—ANCOVA—WOCF (modified intent-to-treat population)						
Visit	Statistic type	Statistic	B244 OD 5.0 (N = 172)	B244 OD 20.0 (N = 172)	Pooled B244 (N = 344)	Vehicle (N = 177)
Week 4 CFB	ANCOVA	n	166	165	331	174
		LSM (SE)	−2.7 (0.185)	−2.7 (0.186)	−2.7 (0.131)	−2.1 (0.181)
		95% CI	(−3.1, −2.4)	(−3.1, −2.3)	(−3.0, −2.5)	(−2.4, −1.7)
	v. Vehicle	LSM difference (SE)	−0.6 (0.259)	−0.6 (0.259)	−0.6 (0.223)	N/A
		90% UCL Adjusted	−0.3	−0.3	−0.3	N/A
		p-value	0.017	0.022	0.0064	N/A
	20.0 v 5.0	LSM difference (SE)	N/A	0.0 (0.268)	N/A	N/A
		90% UCL adjusted	N/A	0.4	N/A	N/A
		p-value	N/A	0.53	N/A	N/A

Mean change in wi-nrs from baseline to Week 4—ANCOVA—LOCF (intent-to-treat population)						
Visit	Statistic type	Statistic	B244 OD 5.0 (N = 180)	B244 OD 20.0 (N = 180)	Pooled B244 (N = 360)	Vehicle (N = 187)
Week 4 CFB	ANCOVA	n	179	180	359	187
		LSM (SE) [4]	−2.7 (0.176)	−2.7 (0.175)	−2.7 (0.124)	−2.0 (0.172)
		95% CI	(−3.0, −2.4)	(−3.0, −2.3)	(−2.9, −2.5)	(−2.4, −1.7)
	v. Vehicle	LSM difference (SE)	−0.7 (0.246)	−0.7 (0.246)	−0.7 (0.212)	N/A
		90% UCL adjusted	−0.4	−0.3	−0.4	N/A
		p-value	0.0088	0.0096	0.0024	N/A
	20.0 v 5.0	LSM difference (SE)	N/A	0.0 (0.254)	N/A	N/A
		90% UCL adjusted	N/A	0.3	N/A	N/A
		p-value	N/A	0.51	N/A	N/A

Proportion of patients with ≥4 point improvement in wi-nrs from baseline to Week 4—primary analysis (modified intent-to-treat population)						
Visit	Statistic type	Statistic	B244 OD 5.0 (N = 172)	B244 OD 20.0 (N = 172)	Pooled B244 (N = 344)	Vehicle (N = 177)
Week 4	Logistic	n/N (proportion %) 95% CI^a^	51/166 (30.7) (23.8, 38.3)	51/165 (30.9) (24.0, 38.6)	102/331 (30.8) (25.9, 36.1)	38/174 (21.8) (15.9, 28.7)
	v. Vehicle	Odds ratio (95% CI)	1.59 (0.97, 2.58)	1.60 (0.98, 2.61)	1.59 (1.04, 2.45)	N/A
		p-value	0.064	0.059	0.033	N/A
	20.0 v 5.0	Odds ratio (95% CI)	N/A	1.01 (0.63, 1.61)	N/A	N/A
		p-value	N/A	0.97	N/A	N/A

Proportion of patients with ≥4 point improvement in WI-NRS from baseline to Week 4—primary analysis (per protocol population)						
Visit	Statistic type	Statistic	B244 OD 5.0 (N = 161)	B244 OD 20.0 (N = 155)	Pooled B244 (N = 316)	Vehicle (N = 167)
Week 4	Logistic	n/N (proportion %) 95% CI^a^	49/158 (31.0) (23.9, 38.8)	49/152 (32.2) (24.9, 40.3)	98/310 (31.6) (26.5, 37.1)	34/164 (20.7) (14.8, 27.7)
	v. Vehicle	Odds ratio (95% CI)	1.72 (1.04, 2.85)	1.82 (1.09, 3.02)	1.77 (1.13, 2.76)	N/A
		p-value	0.036	0.021	0.013	N/A
	20.0 v 5.0	Odds ratio (95% CI)	N/A	1.06 (0.66, 1.71)	N/A	N/A
		p-value	N/A	0.82	N/A	N/A

Proportion of patients with ≥4 point improvement in WI-NRS from baseline to Weeks 4–non-responder imputation (NRI) analysis (per-protocol population)						
Visit	Statistic type	Statistic	B244 OD 5.0 (N = 161)	B244 OD 20.0 (N = 155)	Pooled B244 (N = 316)	Vehicle (N = 167)
Week 4	Logistic	n/N (proportion %) 95% CI^a^	47/158 (29.7) (22.7, 37.5)	49/152 (32.2) (24.9, 40.3)	96/310 (31.0) (25.9, 36.4)	34/164 (20.7) (14.8, 27.7)
	v. Vehicle	Odds ratio (95% CI)	1.62 (0.97, 2.69)	1.82 (1.09, 3.02)	1.72 (1.10, 2.68)	N/A
		p-value	0.064	0.021	0.018	N/A
	20.0 v 5.0	Odds ratio (95% CI)	N/A	1.12 (0.69, 1.82)	N/A	N/A
		p-value	N/A	0.64	N/A	N/A

Proportion of patients with ≥4 point improvement in WI-NRS from baseline to Week 4—LOCF (intent-to-treat population)						
Visit	Statistic Type	Statistic	B244 O.D. 5.0 (N = 180)	B244 O.D. 20.0 (N = 180)	Pooled B244 (N = 360)	Vehicle (N = 187)
Week 4	Logistic	n/N (proportion %) 95% CI^a^	53/179 (29.6) (23.0, 36.9)	52/180 (28.9) (22.4, 36.1)	105/359 (29.2) (24.6, 34.3)	39/187 (20.9) (15.3, 27.4)
	v. Vehicle	Odds ratio (95% CI)	1.60 (0.99, 2.57)	1.54 (0.96, 2.49)	1.57 (1.03, 2.39)	N/A
		p-value	0.055	0.076	0.036	N/A
	20.0 v 5.0	Odds ratio (95% CI)	N/A	0.97 (0.61, 1.52)	N/A	N/A
		p-value	N/A	0.88	N/A	N/A

Mean change in IGA from baseline to Week 4–primary analysis (modified intent-to-treat population)						
Visit	Statistic type	Statistic	B244 OD 5.0 (N = 172)	B244 OD 20.0 (N = 172)	Pooled B244 (N = 344)	Vehicle (N = 177)
Week 4 CFB	MMRM	n	166	164	330	171
		LSM (SE)	−0.8 (0.056)	−0.8 (0.056)	−0.8 (0.046)	−0.5 (0.064)
		95% CI	(−0.9, −0.7)	(−0.9, −0.7)	(−0.9, −0.7)	(−0.7, −0.4)
	v. Vehicle	LSM difference (SE)	−0.3 (0.085)	−0.3 (0.085)	−0.3 (0.079)	N/A
		95% CI	(−0.4, −0.1)	(−0.5, −0.1)	(−0.4, −0.1)	N/A
		p-value	0.0026	0.0005	0.0004	N/A
	20.0 v. 5.0	LSM difference (SE)	N/A	−0.1 (0.096)	N/A	N/A
		95% CI	N/A	(−0.3, 0.1)	N/A	N/A
		p-value	N/A	0.34	N/A	N/A

Mean change in IGA from baseline to Week 4–sub-analysis (modified intent-to-treat population)						
Visit	Statistic type	Statistic	B244 OD 5.0 (N = 172)	B244 OD 20.0 (N = 172)	Pooled B244 (N = 344)	Vehicle (N = 177)
Week 4 CFB	MMRM	n	157	150	307	161
		LSM (SE)	−0.8 (0.057)	−0.8 (0.058)	−0.8 (0.047)	−0.5 (0.065)
		95% CI	(−0.9, −0.7)	(−0.9, −0.7)	(−0.9, −0.7)	(−0.7, −0.4)
	v. Vehicle	LSM difference (SE)	−0.3 (0.087)	−0.3 (0.087)	−0.3 (0.081)	N/A
		95% CI	(−0.4, −0.1)	(−0.5, −0.1)	(−0.4, −0.1)	N/A
		p-value	0.0022	0.001	0.0006	N/A
	20.0 v. 5.0	LSM difference (SE)	N/A	−0.1 (0.099)	N/A	N/A
		95% CI	N/A	(-0.2, 0.1)	N/A	N/A
		p-value	N/A	0.61	N/A	N/A

Mean change in IGA from baseline to Week 4—WOCF (modified intent-to-treat population)						
Visit	Statistic type	Statistic	B244 O.D. 5.0 (N = 172)	B244 O.D. 20.0 (N = 172)	Pooled B244 (N = 344)	Vehicle (N = 177)
Week 4 CFB	MMRM	n	167	164	331	171
		LSM (SE)	−0.7 (0.055)	−0.8 (0.055)	−0.7 (0.045)	−0.5 (0.063)
		95% CI	(−0.8, −0.6)	(−0.9, −0.6)	(−0.8, −0.7)	(−0.6, −0.4)
	v. Vehicle	LSM difference (SE)	−0.2 (0.084)	−0.3 (0.084)	−0.2 (0.078)	N/A
		95% CI	(−0.4, −0.1)	(−0.4, −0.1)	(−0.4, −0.1)	N/A
		p-value	0.0045	0.0023	0.0016	N/A
	20.0 v. 5.0	LSM difference (SE)	N/A	0.0 (0.095)	N/A	N/A
		95% CI	N/A	(−0.2, 0.1)	N/A	N/A
		p-value	N/A	0.66	N/A	N/A

Mean change in IGA from baseline to Week 4—LOCF (intent-to-treat population)						
Visit	Statistic type	Statistic	B244 O.D. 5.0 (N = 180)	B244 O.D. 20.0 (N = 180)	Pooled B244 (N = 360)	Vehicle (N = 187)
Week 4 CFB	MMRM	n	180	180	360	187
		LSM (SE)	−0.8 (0.054)	−0.8 (0.054)	−0.8 (0.044)	−0.5 (0.062)
		95% CI	(−0.9, −0.6)	(−0.9, −0.7)	(−0.9, −0.7)	(−0.6, −0.4)
	v. Vehicle	LSM difference (SE)	−0.3 (0.082)	−0.3 (0.082)	−0.3 (0.076)	N/A
		95% CI	(−0.4, −0.1)	(−0.4, −0.1)	−0.4, −0.1)	N/A
		p-value	0.0023	0.0009	0.0006	N/A
	20.0 v. 5.0	LSM difference (SE)	N/A	−0.1 (0.092)	N/A	N/A
		95% CI	N/A	(−0.3, 0.1)	N/A	N/A
		p-value	N/A	0.46	N/A	N/A

Proportion of patients with IGA of clear or almost clear and ≥2 point improvement from baseline to Week, 4–primary analysis (modified intent-to-treat population)						
Visit	Statistic type	Statistic	B244 OD 5.0 (N = 172)	B244 OD 20.0 (N = 172)	Pooled B244 (N = 344)	Vehicle (N = 177)
Week 4	Logistic	n/N (Proportion %) 95% CI^a^	36/166 (21.7) (15.7, 28.7)	43/164 (26.2) (19.7, 33.6)	79/330 (23.9) (19.4, 28.9)	21/171 (12.3) (7.8, 18.2)
	v. Vehicle	Odds ratio (95% CI)	1.98 (1.10, 3.56)	2.54 (1.43, 4.51)	2.25 (1.33, 3.79)	N/A
		p-value	0.023	0.0015	0.0024	N/A
	20.0 v 5.0	Odds ratio (95% CI)	N/A	1.28 (0.77, 2.13)	N/A	N/A
		p-value	N/A	0.34	N/A	N/A

Proportion of patients with IGA of clear or almost clear and ≥2 point improvement from baseline to Week 4—sub-analysis (modified intent-to-treat population)						
Visit	Statistic type	Statistic	B244 OD 5.0 (N = 172)	B244 OD 20.0 (N = 172)	Pooled B244 (N = 344)	Vehicle (N = 177)
Week 4	logistic	n/N (Proportion %) 95% CI^a^	35/157 (22.3) (16.0, 29.6)	40/150 (26.7) (19.8, 34.5)	75/307 (24.4) (19.7, 29.6)	19/161 (11.8) (7.3, 17.8)
	v. Vehicle	Odds ratio (95% CI)	2.14 (1.17, 3.94)	2.72 (1.49, 4.95)	2.42 (1.40, 4.17)	N/A
		p-value	0.014	0.0011	0.0015	N/A
	20.0 v 5.0	Odds ratio (95% CI)	N/A	1.27 (0.75, 2.14)	N/A	N/A
		p-value	N/A	0.37	N/A	N/A

Proportion of patients with IGA of clear or almost clear and ≥2 point improvement from baseline to Week 4—non-responder imputation (NRI) analysis (modified intent-to-treat population)						
Visit	Statistic type	Statistic	B244 OD 5.0 (N = 172)	B244 OD 20.0 (N = 172)	Pooled B244 (N = 344)	Vehicle (N = 177)
Week 4	Logistic	n/N (proportion %) 95% CI^a^	35/167 (21.0) (15.1, 27.9)	40/164 (24.4) (18.0, 31.7)	75/331 (22.7) (18.3, 27.6)	19/171 (11.1) (6.8, 16.8)
	v. Vehicle	Odds ratio (95% CI)	2.12 (1.16, 3.89)	2.58 (1.42, 4.68)	2.34 (1.36, 4.03)	N/A
		p-value	0.015	0.0018	0.0021	N/A
	20.0 v 5.0	Odds ratio (95% CI)	N/A	1.22 (0.73, 2.04)	N/A	N/A
		p-value	N/A	0.46	N/A	N/A

Proportion of patients with IGA of clear or almost clear and ≥2 point improvement from baseline to Week 4—LOCF (intent-to-treat population)						
Visit	Statistic type	Statistic	B244 O.D. 5.0 (N = 180)	B244 O.D. 20.0 (N = 180)	Pooled B244 (N = 360)	Vehicle (N = 187)
Week 4	Logistic	n/N (proportion %) 95% CI^a^	38/180 (21.1) (15.4, 27.8)	44/180 (24.4) (18.4, 31.4)	82/360 (22.8) (18.5, 27.5)	23/187 (12.3) (8.0, 17.9)
	v. Vehicle	Odds ratio (95% CI)	1.91 (1.09, 3.36)	2.31 (1.33, 4.01)	2.10 (1.27, 3.47)	N/A
		p-value	0.025	0.0031	0.0036	N/A
	20.0 v 5.0	Odds ratio (95% CI)	N/A	1.21 (0.74, 1.98)	N/A	N/A
		p-value	N/A	0.45	N/A	N/A

Mean change in EASI total score from baseline to Week 4–primary analysis (modified intent-to-treat population)						
Visit	Statistic type	Statistic	B244 OD 5.0 (N = 172)	B244 OD 20.0 (N = 172)	Pooled B244 (N = 344)	Vehicle (N = 177)
Week 4 CFB	MMRM	n	166	164	330	171
		LSM (SE)	−3.9 (0.249)	−4.0 (0.250)	−4.0 (0.208)	−3.1 (0.289)
		95% CI	(−4.4, −3.5)	(−4.5, −3.6)	(−4.4, −3.6)	(−3.7, −2.6)
	v. Vehicle	LSM difference (SE)	−0.8 (0.382)	−0.9 (0.382)	−0.9 (0.356)	N/A
		95% CI	(−1.6, −0.1)	(−1.7, −0.2)	(−1.6, −0.2)	N/A
		p-value	0.035	0.017	0.016	N/A
	20.0 v. 5.0	LSM difference (SE)	N/A	−0.4 (0.426)	N/A	N/A
		95% CI	N/A	(−1.2, 0.5)	N/A	N/A
		p-value	N/A	0.38	N/A	N/A

Mean change in EASI total score from baseline to Week 4—sub-analysis (modified intent-to-treat population)						
Visit	Statistic type	Statistic	B244 OD 5.0 (N = 172)	B244 OD 20.0 (N = 172)	Pooled B244 (N = 344)	Vehicle (N = 177)
Week 4 CFB	MMRM	n	157	150	307	161
		LSM (SE)	−4.0 (0.250)	−4.0 (0.253)	−4.0 (0.208)	−3.2 (0.289)
		95% CI	(−4.5, −3.5)	(−4.5, −3.5)	(−4.4, −3.6)	(−3.7, −2.6)
	v. Vehicle	LSM difference (SE)	−0.9 (0.382)	−0.8 (0.384)	−0.8 (0.356)	N/A
		95% CI	(−1.6, −0.1)	(−1.6, −0.1)	(−1.5, −0.1)	N/A
		p-value	0.026	0.030	0.018	N/A
	20.0 v. 5.0	LSM difference (SE)	N/A	−0.1 (0.435)	N/A	N/A
		95% CI	N/A	(−1.0, 0.7)	N/A	N/A
		p-value	N/A	0.80	N/A	N/A

Mean change in EASI total score from baseline to Week 4–WOCF (modified intent-to-treat population)						
Visit	Statistic type	Statistic	B244 O.D. 5.0 (N = 172)	B244 O.D. 20.0 (N = 172)	Pooled B244 (N = 344)	Vehicle (N = 177)
Week 4 CFB	MMRM	n	167	164	331	171
		LSM (SE)	−3.8 (0.244)	−3.8 (0.245)	−3.8 (0.205)	−2.9 (0.286)
		95% CI	(−4.3, −3.3)	(−4.2, −3.3)	(−4.2, −3.4)	(−3.5, −2.3)
	v. Vehicle	LSM difference (SE)	−0.9 (0.376)	−0.8 (0.377)	−0.9 (0.352)	N/A
		95% CI	(−1.6, −0.1)	(−1.6, −0.1)	(−1.6, −0.2)	N/A
		p-value	0.02	0.025	0.015	N/A
	20.0 v. 5.0	LSM difference (SE)	N/A	−0.1 (0.426)	N/A	N/A
		95% CI	N/A	(−0.9, 0.8)	N/A	N/A
		p-value	N/A	0.86	N/A	N/A

Mean change in EASI total score from baseline to Week 4—LOCF (intent-to-treat population)						
Visit	Statistic type	Statistic	B244 O.D. 5.0 (N = 180)	B244 O.D. 20.0 (N = 180)	Pooled B244 (N = 360)	Vehicle (N = 187)
Week 4 CFB	MMRM	n	180	180	360	187
		LSM (SE)	−3.7 (0.242)	−3.7 (0.242)	−3.7 (0.203)	−2.9 (0.282)
		95% CI	(−4.2, −3.2)	(−4.2, −3.2)	(−4.1, −3.3)	(−3.4, −2.3)
	v. Vehicle	LSM difference (SE)	−0.8 (0.372)	−0.8 (0.372)	−0.8 (0.348)	N/A
		95% CI	(−1.6, −0.1)	(−1.6, −0.1)	(−1.5, −0.1)	N/A
		p-value	0.024	0.027	0.017	N/A
	20.0 v. 5.0	LSM difference (SE)	N/A	−0.2 (0.416)	N/A	N/A
		95% CI	N/A	(−1.0, 0.6)	N/A	N/A
		p-value	N/A	0.59	N/A	N/A

Proportion of patients with ≥75% improvement in EASI (EASI-75) from baseline to Week 4–primary analysis (modified intent-to-treat population)						
Visit	Statistic type	Statistic	B244 OD 5.0 (N = 172)	B244 OD 20.0 (N = 172)	Pooled B244 (N = 344)	Vehicle (N = 177)
Week 4	Logistic	n/N (proportion %) 95% CI^a^	46/166 (27.7) (21.1, 35.2)	48/164 (29.3) (22.4, 36.9)	94/330 (28.5) (23.7, 33.7)	27/171 (15.8) (10.7, 22.1)
	v. Vehicle	Odds ratio (95% CI)	2.04 (1.20, 3.49)	2.21 (1.30, 3.75)	2.12 (1.32, 3.42)	N/A
		p-value	0.0086	0.0035	0.0019	N/A
	20.0 v 5.0	Odds ratio (95% CI)	N/A	1.08 (0.67, 1.74)	N/A	N/A
		p-value	N/A	0.75	N/A	N/A

Proportion of patients with ≥75% improvement in EASI (EASI-75) from baseline to Week 4—sub-analysis (modified intent-to-treat population)						
Visit	Statistic type	Statistic	B244 OD 5.0 (N = 172)	B244 OD 20.0 (N = 172)	Pooled B244 (N = 344)	Vehicle (N = 177)
Week 4	Logistic	n/N (proportion %) 95% CI^a^	46/157 (29.3) (22.3, 37.1)	43/150 (28.7) (21.6, 36.6)	89/307 (29.0) (24.0, 34.4)	24/161 (14.9) (9.8, 21.4)
	v. Vehicle	Odds ratio (95% CI)	2.37 (1.36, 4.11)	2.29 (1.31, 4.02)	2.33 (1.42, 3.84)	N/A
		p-value	0.0023	0.0036	0.0009	N/A
	20.0 v 5.0	Odds ratio (95% CI)	N/A	0.97 (0.59, 1.59)	N/A	N/A
		p-value	N/A	0.9	N/A	N/A

Proportion of patients with ≥75% improvement in EASI (EASI-75) from baseline to Week 4—non-responder imputation (NRI) analysis (modified intent-to-treat population)						
Visit	Statistic type	Statistic	B244 OD 5.0 (N = 172)	B244 OD 20.0 (N = 172)	Pooled B244 (N = 344)	Vehicle (N = 177)
Week 4	Logistic [1, 2]	n/N (proportion %) 95% CI^a^	46/167 (27.5) (20.9, 35.0)	43/164 (26.2) (19.7, 33.6)	89/331 (26.9) (22.2, 32.0)	24/171 (14.0) (9.2, 20.2)
	v. Vehicle	Odds ratio (95% CI)	2.33 (1.34, 4.03)	2.18 (1.25, 3.79)	2.25 (1.37, 3.70)	N/A
		p-value	0.0026	0.006	0.0013	N/A
	20.0 v 5.0	Odds ratio (95% CI)	N/A	0.93 (0.57, 1.52)	N/A	N/A
		p-value	N/A	0.79	N/A	N/A

Proportion of patients with ≥75% improvement in EASI (EASI-75) from baseline to Week 4—LOCF (intent-to-treat population)						
Visit	Statistic type	Statistic	B244 O.D. 5.0 (N = 180)	B244 O.D. 20.0 (N = 180)	Pooled B244 (N = 360)	Vehicle (N = 187)
Week 4	Logistic	n/N (proportion %) 95% CI^a^	47/180 (26.1) (19.9, 33.2)	49/180 (27.2) (20.9, 34.3)	96/360 (26.7) (22.2, 31.6)	28/187 (15.0) (10.2, 20.9)
	v. Vehicle	Odds ratio (95% CI)	2.01 (1.19, 3.38)	2.12 (1.26, 3.57)	2.06 (1.30, 3.29)	N/A
		p-value	0.0089	0.0044	0.0022	N/A
	20.0 v 5.0	Odds ratio (95% CI)	N/A	1.06 (0.66, 1.69)	N/A	N/A
		p-value	N/A	0.81	N/A	N/A

Primary analysis required that at least one WI-NRS rating be reported in a week for the weekly average to be calculated.

ANCOVA, Analysis of covariance. CFB, Change from Baseline. LSM, Least Squares Mean. SE, Standard Error. UCL, Upper Confidence Limit for one-sided confidence interval. CI, confidence interval. MMRM, Mixed Model with Repeated Measures. Logistic, Logistic regression model. WI-NRS, Worst itch numerical rating scale. WOCF, Worst observation carried forward. LOCF, Last observation carried forward. NRI, Non responder imputation. IGA, Investigator global assessment. EASI, Eczema area and severity index.

a n is the number of patients who have improved at that visit. N is the number of patients with data at that visit.

**Fig. 3:**
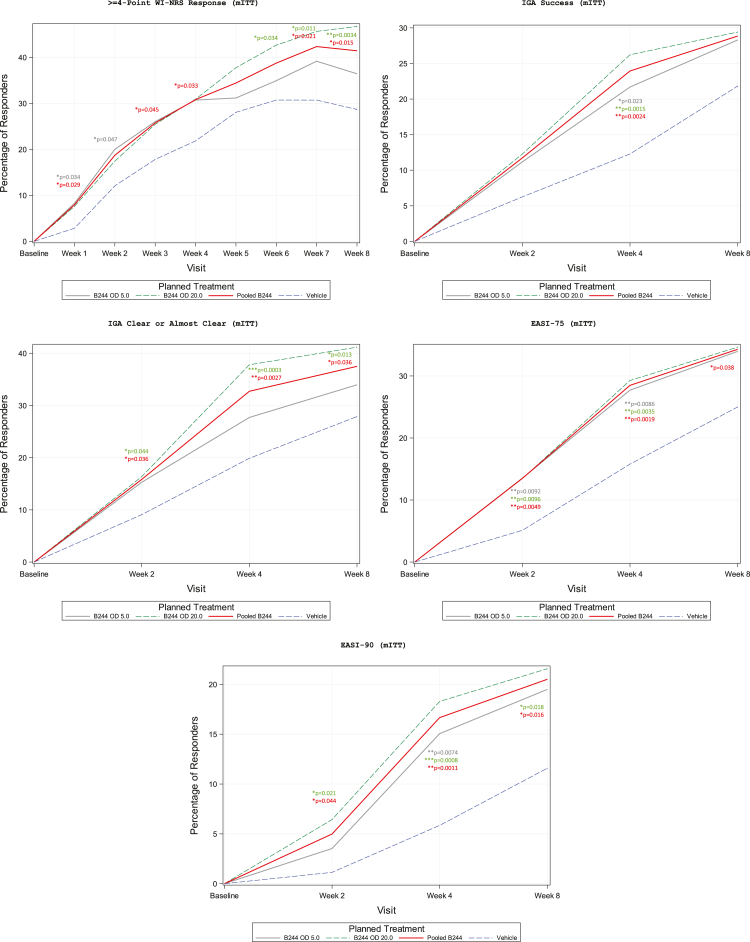
Proportion of patients achieving itch, IGA, and EASI response in the modified intent-to-treat population. Data is shown as percentage of responders. WI-NRS, Worst itch numerical rating scale. IGA, Investigator global assessment. EASI, Eczema area and severity index.

A total of 31% (51/166) of the patients in the OD 5.0 treatment group had a ≥4 point improvement in WI-NRS from baseline to Week 4 and a total of 31% (51/165) in the OD 20.0 treatment group, compared to only 22% (38/174) of the patients in the vehicle group in the mITT population (Table 2, Fig. 3). Although OD 5.0 and OD 20.0 groups approached a significantly greater proportion of patients with ≥4 point improvement (odds ratio (OR) = 1.59; p = 0.064 and OR = 1.60; p = 0.059, respectively), pooled B244 was the only group to reach a level of statistical significance (OR = 1.59; p = 0.033) compared to the vehicle group, likely due to the larger sample size. No difference was observed comparing OD 5.0–OD 20.0 (OR = 1.01; p = 0.97). In the PP population, all B244 groups had a significantly higher proportion of patients who experienced a ≥4 point improvement in WI-NRS from baseline to Week 4 compared to patients in the vehicle group. That is, 31% (49/158), 32% (49/152), and 32% (98/310) of patients in OD 5.0 (OR = 1.72; p = 0.036), OD 20.0 (OR = 1.82; p = 0.021), and pooled B244 (OR = 1.77; p = 0.013), respectively, compared to 21% (34/164) for the vehicle group, experienced this level of improvement. Similar to the mITT population, no difference was observed comparing OD 5.0–OD 20.0 (OR = 1.06; p = 0.82). In the NRI and ITT analysis, results were consistent with that of the primary analysis of proportion of patients that had a ≥4 point improvement in WI-NRS from baseline to Week 4.

Patients in the B244 treatment groups showed significantly larger reductions in their IGA score from baseline to Week 4 compared to patients in the vehicle group. Treatment with either dose of B244 (OD 5.0 or OD 20.0) demonstrated a significant treatment effect in mean change in IGA from baseline versus the vehicle group at Week 4 in the mITT population (Table 2) and was similar in the PP population. The mean reduction from baseline was equivalent for the OD 5.0 and the OD 20.0 treatment groups with LSM change from baseline = −0.8 (95% CI −0.9, −0.7). The decrease from baseline was less for the vehicle group with LSM = −0.5 (95% CI −0.7, −0.4). The LSM difference was −0.3 [p = 0.0026] for OD 5.0, −0.3 [p = 0.0005] for OD 20.0, and −0.3 [p = 0.0004] for pooled B244 (Fig. 2). No difference was detected between OD 5.0 and OD 20.0 (LSM difference of −0.1 [p = 0.34]). In the sub-analysis, ITT analysis and worst-case analysis, results were consistent with that of the primary analysis for mean change in IGA score from baseline to Week 4.

A total of 22% (36/166) of the patients in the OD 5.0 treatment group, 26% (43/164) in the OD 20.0 treatment group, and 24% (79/330) of patients in the pooled B244 group had a ≥2 point improvement in IGA to Clear or Almost Clear from baseline to Week 4, compared to only 12% (21/171) of patients in the vehicle group in the mITT population (Table 2) and was similar in the PP population. All B244 groups had a significantly higher proportion of patients who experienced this response rate compared to patients in the vehicle group (OR = 1.98, p = 0.023 for OD 5.0; OR = 2.54, p-value = 0.0015 for OD 20.0; and OR = 2.25, p = 0.0024 for pooled B244) (Fig. 3). Similar to other analyses, no difference was observed between OD 5.0 and OD 20.0 at Week 4 (OR = 1.28, p = 0.34). Results for the sub-analysis, NRI analysis, and ITT analysis were consistent with that of the primary analysis for proportion of patients with ≥2 point improvement in IGA to Clear or Almost Clear from baseline to Week 4.

Patients in the B244 treatment groups showed significantly larger reductions in their EASI total score from baseline to Week 4 compared to patients in the vehicle group. Treatment with either dose of B244 (OD 5.0 or OD 20.0) demonstrated a significant treatment effect versus the vehicle group at Week 4 in the mean change in EASI total score from baseline to Week 4 in the mITT population (Table 2) and was similar in the PP population. The mean reduction in EASI total score from baseline was LSM change from baseline = −3.9 (95% CI −4.4, −3.5) for the OD 5.0 treatment group and LSM = −4.0 (95% CI −4.5, −3.6) for the OD 20.0 group. The decrease from baseline was less for the vehicle group with LSM = −3.1 (95% CI −3.7, −2.6). The LSM difference was −0.8 [p = 0.035] for OD 5.0, −0.9 [p = 0.017] for OD 20.0, and −0.9 [p = 0.016] for pooled B244 (Fig. 2). No difference was detected between OD 5.0 and OD 20.0 (LSM difference of −0.4 [p = 0.38]). In the sub-analysis, ITT analysis and worst-case analysis, results were consistent with that of the primary analysis.

A total of 28% (46/166) of the patients in the OD 5.0 treatment group, 29% (48/164) in the OD 20.0 treatment group, and 29% (94/330) of patients in the pooled B244 group had a ≥75% improvement in EASI from baseline to Week 4 compared to 16% (27/171) of patients in the vehicle group in the mITT population (Table 2) and was similar in the PP population. All B244 groups had a significantly higher proportion of patients who experienced this response rate compared to patients in the vehicle group (OR = 2.04, p = 0.0086 for OD 5.0; OR = 2.21, p = 0.0035 for OD 20.0; and OR = 2.12, p = 0.0019 for pooled B244) (Fig. 3). Similar to other analyses, no difference was observed between OD 5.0 and OD 20.0 at Week 4 (OR = 1.08, p = 0.75). The results for the sub-analysis, NRI analysis, and ITT analysis were consistent with the proportion of patients with ≥75% improvement in EASI total score from baseline to Week 4.

For onset of treatment effect in itch, both B244 treatment groups experienced a significantly larger reduction from baseline compared to the vehicle group at Week 2 and as early as Week 1 with separation between B244 treatment groups and vehicle group starting as early as Day 2 (Fig. 2 and Appendix). Durability of treatment effect was observed at Week 8 after 4 weeks on-treatment and 4 weeks off-treatment across WI-NRS, IGA, and EASI measures (Fig. 2 and Appendix). Stratified analysis on baseline characteristics for WI-NRS, IGA, and EASI measures showed similar results. Efficacy outcomes were also similar across the 3 baseline BSA strata (10–20%, >20–30%, >30–40%).

B244 was well tolerated in this study, with most treatment-emergent adverse events (TEAEs) classified as mild to moderate. Overall, 33 (18%) of the patients in the B244 OD 5.0 group, 29 16% in the B244 OD 20.0 group, and 17 (9%) in the vehicle group reported at least one TEAE in the study (Table 3). Specific TEAEs were well balanced among the groups. For all groups, TEAEs were most frequently reported in musculoskeletal and connective tissue disorders and nervous system disorders. Headache and arthalgia were reported to be the most frequent TEAEs in the study (≥2% of patients in any group), but all were determined to be unrelated to treatment and not dose dependent, were mild in severity, and did not lead to any study discontinuations. One patient in the B244 OD 20.0 group reported a Grade 3 TEAE of back pain which was not considered to be related to the IP.

**Table 3:** Treatment-emergent adverse events in the safety population.

Overall summary of treatment-emergent adverse events (safety population)					
	Statistic	B244 OD 5.0 (N = 180)	B244 OD 20.0 (N = 180)	Pooled B244 (N = 360)	Vehicle (N = 186)
Total number of TEAEs	n	44	36	80	20
Total number of TESAEs	n	0	0	0	0
Number of patients with:					
At least one TEAE	n (%)	33 (18)	29 (16)	62 (17)	17 (9)
At least one treatment-related TEAE	n (%)	5 (3)	6 (3)	10 (3)	3 (2)
At least one severe (Grade 3) TEAE	n (%)	0	1 (1)	1 (0.3)	0
At least one treatment-related severe (Grade 3) TEAE	n (%)	0	0	0	0
At least one TEAE leading to study discontinuation	n (%)	2 (1)	2 (1)	4 (1)	0
At least one TEAE leading to death	n (%)	0	0	0	0
At least one TESAE	n (%)	0	0	0	0
At least one treatment-related TESAE	n (%)	0	0	0	0

Summary of treatment-emergent adverse events with ≥2% frequency by system organ class and preferred term (safety population)					
	Statistic	B244 OD 5.0 (N = 180)	B244 OD 20.0 (N = 180)	Pooled B244 (N = 360)	Vehicle (N = 186)
Total number of TEAEs	n	44	36	80	20
Number of patients with at least one TEAE	n (%)	33 (18)	29 (16)	62 (17)	17 (9)
Musculoskeletal and connective tissue disorders	n (%)	2 (1)	10 (6)	12 (3)	4 (2)
Arthralgia	n (%)	1 (1)	4 (2)	5 (1)	0
Nervous system disorders	n (%)	6 (3)	5 (3)	11 (3)	1 (1)
Headache	n (%)	6 (3)	4 (2)	10 (3)	1 (1)

Summary of treatment-emergent adverse events leading to discontinuation from study by system organ class and preferred term (safety population)					
	Statistic	B244 OD 5.0 (N = 180)	B244 OD 20.0 (N = 180)	Pooled B244 (N = 360)	Vehicle (N = 186)
Total number of TEAEs	n	2	2	4	0
Number of patients with at least one TEAE	n (%)	2 (1)	2 (1)	4 (1)	0
Infections and infestations	n (%)	2 (1)	0	2 (1)	0
COVID-19	n (%)	2 (1)	0	2 (1)	0
Skin and subcutaneous tissue disorders	n (%)	0	2 (1)	2 (1)	0
Dermatitis atopic	n (%)	0	2 (1)	2 (1)	0

Summary of treatment-emergent adverse events by system organ class and preferred term (safety population)					
	Statistic	B244 OD 5.0 (N = 180)	B244 OD 20.0 (N = 180)	Pooled B244 (N = 360)	Vehicle (N = 186)
Total number of TEAEs	n	44	36	80	20
Number of patients with at least one TEAE	n (%)	33 (18)	29 (16)	62 (17)	17 (9)
Gastrointestinal disorders	n (%)	5 (3)	4 (2)	9 (3)	2 (1)
Abdominal pain	n (%)	1 (1)	0	1 (0.3)	0
Abdominal pain upper	n (%)	0	2 (1)	2 (1)	0
Diarrhea	n (%)	0	1 (1)	1 (0.3)	0
Dyspepsia	n (%)	2 (1)	0	2 (1)	0
Gastroesophageal reflux disease	n (%)	0	0	0	1 (1)
Nausea	n (%)	1 (1)	0	1 (0.3)	1 (1)
Toothache	n (%)	1 (1)	1 (1)	2 (1)	0
General disorders and administration site conditions	n (%)	6 (3)	3 (2)	9 (3)	4 (2)
Application site dryness	n (%)	0	0	0	1 (1)
Application site erythema	n (%)	2 (1)	0	2 (1)	0
Application site pain	n (%)	1 (1)	1 (1)	2 (1)	2 (1)
Application site pruritus	n (%)	3 (2)	0	3 (1)	0
Application site rash	n (%)	1 (1)	1 (1)	2 (1)	0
Fatigue	n (%)	0	0	0	1 (1)
Pyrexia	n (%)	0	1 (1)	1 (0.3)	0
Vaccination site discomfort	n (%)	3 (2)	0	3 (1)	1 (1)
Immune system disorders	n (%)	1 (1)	0	1 (0.3)	0
Food allergy	n (%)	1 (1)	0	1 (0.3)	0
Infections and infestations	n (%)	7 (4)	6 (3)	13 (4)	3 (2)
COVID-19	n (%)	2 (1)	3 (2)	5 (1)	1 (1)
Gastroenteritis viral	n (%)	0	2 (1)	2 (1)	0
Hordeolum	n (%)	1 (1)	0	1 (0.3)	0
Influenza	n (%)	1 (1)	0	1 (0.3)	0
Nasopharyngitis	n (%)	1 (1)	1 (1)	2 (1)	0
Urinary tract infection	n (%)	2 (1)	0	2 (1)	2 (1)
Vulvovaginal candidiasis	n (%)	0	0	0	1 (1)
Injury, poisoning and procedural complications	n (%)	2 (1)	1 (1)	3 (1)	0
Hand fracture	n (%)	0	1 (1)	1 (0.3)	0
Ligament sprain	n (%)	2 (1)	0	2 (1)	0
Investigations	n (%)	1 (1)	0	1 (0.3)	3 (2)
Aspartate aminotransferase increased	n (%)	0	0	0	1 (1)
Creatinine renal clearance decreased	n (%)	0	0	0	1 (1)
Hepatic enzyme increased	n (%)	1 (1)	0	1 (0.3)	0
SARS-CoV-2 test positive	n (%)	0	0	0	1 (1)
Metabolism and nutrition disorders	n (%)	0	1 (1)	1 (0.3)	0
Diabetes mellitus	n (%)	0	1 (1)	1 (0.3)	0
Musculoskeletal and connective tissue disorders	n (%)	2 (1)	10 (6)	12 (3)	4 (2)
Arthralgia	n (%)	1 (1)	4 (2)	5 (1)	0
Back pain	n (%)	1 (1)	3 (2)	4 (1)	2 (1)
Myalgia	n (%)	0	1 (1)	1 (0.3)	1 (1)
Neck pain	n (%)	0	1 (1)	1 (0.3)	0
Pain in extremity	n (%)	0	1 (1)	1 (0.3)	1 (1)
Nervous system disorders	n (%)	6 (3)	5 (3)	11 (3)	1 (1)
Headache	n (%)	6 (3)	4 (2)	10 (3)	1 (1)
Lethargy	n (%)	0	1 (1)	1 (0.3)	0
Reproductive system and breast disorders	n (%)	1 (1)	1 (1)	2 (1)	0
Ovarian cyst ruptured	n (%)	0	1 (1)	1 (0.3)	0
Pelvic pain	n (%)	1 (1)	0	1 (0.3)	0
Respiratory, thoracic and mediastinal disorders	n (%)	1 (1)	1 (1)	2 (1)	0
Cough	n (%)	1 (1)	0	1 (0.3)	0
Epistaxis	n (%)	0	1 (1)	1 (0.3)	0
Skin and subcutaneous tissue disorders	n (%)	3 (2)	4 (2)	7 (2)	0
Acne	n (%)	0	1 (1)	1 (0.3)	0
Blister	n (%)	1 (1)	0	1 (0.3)	0
Dermatitis allergic	n (%)	1 (1)	0	1 (0.3)	0
Dermatitis atopic	n (%)	0	2 (1)	2 (1)	0
Pruritus	n (%)	1 (1)	1 (1)	2 (1)	0
Surgical and medical procedures	n (%)	0	0	0	1 (1)
Cataract operation	n (%)	0	0	0	1 (1)
Vascular disorders	n (%)	1 (1)	0	1 (0.3)	0
Hypertension	n (%)	1 (1)	0	1 (0.3)	0

TEAE, Treatment-emergent adverse event. TESAE, Treatment-emergent severe adverse event. OD, Optical density.

A summary of TEAEs by system organ class and preferred term is provided in Table 3. Treatment-emergent adverse events at least possibly related to IP were reported in five (3%) of the patients in the OD 5.0 group, six (3%) of the patients in the OD 20.0 group, and in three (2%) of the patients in the vehicle group. Overall, there was one (1%) event of application site dryness in the vehicle group (probably related) and none in the B244 groups. There were two (1%) events of application site erythema in the OD 5.0 group: one (1%) event was possibly related and one (1%) event was probably related to the IP. There were two (1%) events of application site pain: one (1%) in the OD 5.0 group (possibly related) and one (1%) in the OD 20.0 group (probably related); there were two (1%) such events in the vehicle group (probably related). There were three (2%) events of application site pruritus in the OD 5.0 group: two (1%) possibly related and one (1%) probably related. There were two (1%) events of application site rash: one (1%) in the OD 5.0 group (possibly related) and one (1%) in the OD 20.0 group (probably related).

TEAEs leading to discontinuation from the study were reported in both the OD 5.0 and OD 20.0 treatment groups. Two (1%) of 180 patients in the OD 5.0 group discontinued from the study due to a COVID-19 infection and two (1%) of 180 patients in the OD 20.0 discontinued due to an event of atopic dermatitis. None of these TEAEs were considered related to the IP. No serious TEAEs were reported in the study. No deaths were reported in the study. Laboratory values, vital signs, and physical examinations were similar between treatment groups and any observed values and change from baseline were not determined to be clinically significant (data not shown). The mean patient-reported local tolerability rating for all symptoms at all timepoints ranged between none and mild (0.3–0.7 range), indicating that the IP is well-tolerated.

## Discussion

Our results indicate that treatment with B244 provides significant improvement of clinical signs and symptoms in adult patients with mild-to-moderate AD. The consistency of these findings was supported by substantial improvements from baseline with B244 treatment compared with vehicle across the board in all measures of AD signs, including IGA and EASI scores, and patient-reported outcomes, including WI-NRS, POEM, and 5-D Pruritus Scale. Patients using B244 OD 5.0 or OD 20.0 twice a day for 4 weeks had similar outcomes with no dose response observed between OD 5.0 and OD 20.0 in efficacy outcomes and no important differences in safety parameters, although there was a trend toward improvement (p = 0.051) in the OD 20.0 group for the proportion of patients with IGA of clear or almost clear at Week 4. Also, similar efficacy outcomes in the 3 baseline BSA strata (10–20%, >20–30%, >30–40%) suggests that 10 pumps of B244 twice a day is sufficient to elicit a treatment response across the range of affected body surface areas evaluated.

The goal of therapy for AD is to restore the epidermal barrier function and reduce skin inflammation, thereby reducing the severity of AD lesions and associated pruritus. To this end, B244 may have multiple mechanisms of action, including the anti-inflammatory actions of nitric oxide and the anti-microbial nature of nitrite. B244 showed immunomodulatory effects and reduced Th2-associated gene expression of IL-4 and cytokine levels of IL-5 and IL-13 *in vitro* which are implicated in AD pathology.^10^ B244 may reduce *Staphylococcus aureus* colonization and decrease Th2 activation, leading to improvements in both signs and symptoms of AD.

Results from the primary analysis on the mITT patient population were consistent with the PP population. Additional analyses were performed to evaluate the effectiveness of the B244 topical spray and the robustness of those results compared to the primary analysis. These included sensitivity analysis (requires at least 4 NRS ratings to be reported in a given week as opposed to only 1 rating required for the primary analysis), sub-analysis, NRI, worst case analysis (parallel to the NRI analysis but for continuous data, patients who discontinued early due to TEAE or who took on-treatment rescue medications had their worst score imputed for assessments that occurred on/after date of discontinuation/on-treatment rescue medication use through the end of the study), and ITT (LOCF was used to impute missing data at Week 4). There were no significant differences among these analyses suggesting robustness and consistency of data.

Significant improvements of pruritus occurred as early as 1 week after treatment with numerical separation from the vehicle group occurring as early as 2 days after treatment. Itch relief was maintained up to Week 8 (after 4 weeks on-treatment and 4 weeks off-treatment) suggesting durability of treatment effect. Furthermore, significant improvement in clinical signs of AD occurred after 4 weeks of treatment which continued to improve at Week 8 suggesting durability. These potential remittive effects will be studied further in future studies which may further suggest that microbiome rebalancing may persist after drug withdrawal.

Currently available topical treatment options for mild to moderate AD may have limited efficacy or carry safety or tolerability concerns. These include topical therapies such as topical corticosteroids (TCS), topical calcineurin inhibitors (TCI), topical phosphodiesterase-4 (PDE-4) inhibitors, or topical Janus kinase (JAK) inhibitors which carry varying safety concerns ranging from skin atrophy, stinging/burning to black box warnings. There is a growing unmet need to safely and effectively treat mild to moderate AD and associated pruritus.

As a topically applied live biotherapeutic, B244 represents a novel class of AD treatment. Multiple approaches for live biotherapeutics to treat AD are in development, including both topical application and oral delivery.^16^ While not all of these approaches have yielded positive clinical results, the success of this study demonstrates that the application of live biotherapeutics is a promising new modality for drug development.

These results must be evaluated in the context of several limitations, including 4 week treatment duration. A future long term extension study will provide up to 1 year of efficacy and safety data for B244. The increasing treatment effects with time for itch and AD clinical signs outcomes observed during the study suggest that longer treatment periods with B244 beyond 4 weeks would continue to improve treatment effects. There was no active comparator arm. The study assessed B244 only in adult patients with AD. Effects of B244 in the pediatric population will be addressed in future studies.

Due to its unique mechanism of action, fast itch relief, excellent safety, broad target patient profile, and patient-friendly administration as a topical spray (including safe use in face, groin, and other steroid sensitive areas), B244 may be an effective treatment for patients with mild-to-moderate atopic dermatitis, including those with severe itch. The fast-acting itch reduction targeting moderate to severe pruritus, sustained effects on eczema lesions, and a track record of safety will justify the use of this treatment once approved. We intend for this product to be used as an early-stage intervention for patients who are unable or unwilling to use other first- or second-line treatment options, and before stepping up to the more expensive treatments that may have undesirable or intolerable side effects.

This was the largest study to date using B244 providing additional important safety information. Overall, treatment with B244 was safe and well tolerated. There were very few adverse events reported and those were of mild or moderate severity. In conclusion, this study demonstrated the efficacy and safety of B244 in adult patients with mild-to-moderate AD and associated pruritus with a favorable benefit-risk profile, and the findings support further development into Phase 3 in adults and children.

## Contributors

All authors conceptualized the study. CL, VS, RB, and HK acquired the data. JS, CL, VS, RB, and HK accessed and verified the data. JS, CL, DB, and HK prepared the original draft. All authors contributed to the review and editing of the draft. All authors provided approval for the submission of the manuscript for publication.

## Data sharing statement

The individual participant data from this study is not currently available for sharing.

## Declaration of interests

JS has received funding from Galderma, Incyte, and Pfizer, has received consulting fees from Abbvie, Alamar, Aldena, Amgen, AObiome, Arcutis, Arena, Asana, Aslan, BioMX, Biosion, Bodewell, Boehringer-Ingelheim, Cara, Castle Biosciences, Celgene, Connect Biopharma, Dermavant, Dermira, Dermtech, Eli Lilly, Galderma, GlaxoSmithKline, Incyte, Kiniksa, Leo Pharma, Menlo, Novartis, Optum, Pfizer, RAPT, Regeneron, Sanofi-Genzyme, Shaperon, Union, UpToDate, has received honoraria from Abbvie, Eli Lilly, Leo Pharma, Pfizer, Regeneron, Sanofi-Genzyme, and Participated on a Data Safety Monitoring Board or Advisory Board for Kymab. PL has received funding from AbbVie, AOBiome, Eczema Foundation, received royalties from Theraflex AIM, has received consulting fees from AbbVie, Almirall, Amyris, ASLAN, Bristol-Myers Squibb, Burt's Bees, Castle Biosciences, Codex Labs, Concerto Biosci, Dermavant, Dermira, DermVeda, Eli Lilly, Galderma, IntraDerm, Janssen, Johnson & Johnson, Kaleido Biosci, Kimberly Clark, LEO Pharma, Lipidor, L'Oreal, Menlo Therapeutics, Merck, Micreos, MyOR Diagnostics, Regeneron/Sanofi Genzyme, Sibel Health, Skinfix, Sonica, Theraplex, UCB, Unilever, has received honoraria from AbbVie, Eli Lilly, Galderma, Hyphens Pharma, Incyte, La Roche- Posay/L'Oreal, MyOR Diagnostics, ParentMD, Pfizer, Pierre-Fabre Dermatologie, Regeneron/Sanofi Genzyme, has received travel support from AbbVie, Eli Lilly, Galderma, Hyphens Pharma, Incyte, La Roche- Posay/L'Oreal, MyOR Diagnostics, ParentMD, Pfizer, Pierre-Fabre Dermatologie, Regeneron/Sanofi Genzyme, has patents pending from Theraflex AIM, is an unpaid board member of the National Eczema Association, and holds stock or stock options from Codex Labs, LearnSkin/Learn Health, MaskSense, Medable, Micreos, Modernizing Medicine, Yobee Care. ES has received grants from AbbVie, Acrotech Biopharma Inc, Amgen, Arcutis, Aslan, Castle Biosciences, CorEvitas, Dermavant, Dermira, Eli Lilly, Incyte, Kymab, Kyowa Kirin, 10.13039/100012308National Jewish Health, Leo, Pfizer, Regeneron, Sanofi, and Target RWE, has received consulting fees from Advances in Cosmetic Medical Derm Hawaii LLC, AbbVie, Amgen, AOBiome LLC, Arcutis Biotherapeutics, Arena Pharmaceuticals, Aslan Pharma, Boehringer Ingelheim USA, Inc., Boston Consulting Group, 10.13039/100002491Bristol Myers Squibb—BMS, Collective Acumen LLC (CA), CorEvitas, Dermira, Eli Lilly, Evelo Biosciences, Evidera, ExcerptaMedica, FIDE, Forte Bio RX, Galderma, Gesellschaft Z, GlaxoSmithKline, Incyte, Janssen, 10.13039/100004331Johnson & Johnson, 10.13039/100016348Kyowa Kirin Pharmaceutical Development, Leo Pharm, Medscape LLC, Merck, MauiDerm, MLG Operating, MJH holding, Pfizer, Physicians World LLC, PRImE, Regeneron, Revolutionizing Atopic Dermatitis Inc, Roivant, Sanofi-Genzyme, Trevi therapeutics, Valeant, Vindico Medical education, WebMD, has received honoraria from AbbVie, Eli Lilly, GlaxoSmithKline, Incyte, Janssen, Kyowa Kirin, Leo, Pfizer, Regeneron, Sanofi, Medscape, has received meeting support from FIDE, MauiDerm, and Sanofi-Regeneron, has served on advisory boards for Arena Eli Lilly, GlaxoSmithKline, Incyte, Janssen, Kyowa Kirin, Leo, Pfizer, Regeneron, Sanofi, and has received payments for serving in a leadership or fiduciary role as Chair of Sanofi-Genzyme and Regeneron US Medical Advisory Board, Chair of Research, Scientific Advisory Committee of the 10.13039/100003980National Eczema Association, Chair of Atopic Dermatitis Expert Resource Group for the AAD, Board Member, International Society for Atopic Dermatitis (ISAD), and Executive Member for the international Harmonizing Outcome Measures in Eczema (HOME) Working Group. CL, DB, IG, JNC, TK, and HK are or were employees of AOBiome. CL has received funding for travel from AOBiome, has patents or patents pending as an employee of AOBiome, and holds stock or stock options in AOBiome. DB has received funding for travel from AOBiome, has patents or patents pending as an employee of AOBiome, and holds stock or stock options in AOBiome. IG has patents or patents pending as an employee of AOBiome and holds stock or stock options in AOBiome. TK has received funding for travel from AOBiome, has patents or patents pending as an employee of AOBiome, and holds stock or stock options in AOBiome. VS is an employee of Biorasi, which received payments associated with the execution and design of the reported study. RB is an employee of Veristat. HK has received funding for travel from AOBiome and holds stock or stock options in AOBiome.
