# Supplementary material for: Efficacy and safety of topically applied therapeutic ammonia oxidising bacteria in adults with mild-to-moderate atopic dermatitis and moderate-to-severe pruritus: a randomised, double-blind, placebo-controlled, dose-ranging, phase 2b trial
Source: eClinicalMedicine. 2023 May 16;60:102002. doi: 10.1016/j.eclinm.2023.102002 (PMC10314159; doi:10.1016/j.eclinm.2023.102002)
Supplement: Appendix [file mmc1.pdf]

CLINICAL STUDY PROTOCOL

TITLE:	A Phase II, Randomized, Double-Blind, Vehicle Controlled Study of the Efficacy, Safety, and Tolerability of B244 Topical Spray for the Treatment of Pruritus in Adults with a History of Atopic Dermatitis
IND Number:	17485
Protocol Number:	PRB244-01
Protocol Version/Date:	Amendment 3 / 28-FEB-2020
Development Phase:	Phase 2
Sponsor:	AOBiome Therapeutics<br>125 Cambridgepark Drive<br>Cambridge, MA 02140<br>USA

Any and all information presented in this document shall be treated as confidential and shall remain the exclusive property of AOBiome Therapeutics (or any of its affiliated companies). The use of such confidential information must be restricted to the recipient for the agreed purpose and must not be disclosed, published or otherwise communicated to any unauthorized persons, for any reasons, in any form whatsoever without the prior written consent of AOBiome Therapeutics (or the concerned affiliated company).

Revision Chronology

Original	September 25, 2019
Amendment 1	November 07, 2019
Amendment 2	January 06, 2020
Amendment 3	February 28, 2020

SIGNATURE PAGE FOR INVESTIGATOR(S)

TITLE:	A Phase II, Randomized, Double-Blind, Vehicle Controlled Study of the Efficacy, Safety, and Tolerability of B244 Topical Spray for the Treatment of Pruritus in Adults with a History of Atopic Dermatitis
IND Number:	17485
Protocol Number:	PRB244-01
Protocol Version/Date:	Amendment 3 / 28-FEB-2020
Development Phase:	Phase 2
Sponsor:	AOBiome Therapeutics<br>125 Cambridgepark Drive<br>Cambridge, MA 02140<br>USA

I have read the protocol and agree to conduct this study in accordance with the protocol, all relevant laws and regulations in force at the time, International Conference on Harmonisation Guidelines for Good Clinical Practices, and the Declaration of Helsinki.

____

Principal Investigator’s printed name

__________________________________ _______________________

Principal Investigator’s signature Date (DD-MMM-YYYY)

SPONSOR PROTOCOL APPROVAL SIGNATURE(S)

TITLE:	A Phase II, Randomized, Double-Blind, Vehicle Controlled Study of the Efficacy, Safety, and Tolerability of B244 Topical Spray for the Treatment of Pruritus in Adults with a History of Atopic Dermatitis
IND Number:	17485
Protocol Number:	PRB244-01
Protocol Version/Date:	Amendment 3 / 28-FEB-2020
Development Phase:	Phase 2
Sponsor:	AOBiome Therapeutics<br>125 Cambridgepark Drive<br>Cambridge, MA 02140<br>USA

I have read the protocol and agree to conduct this study in accordance with the protocol, all relevant laws and regulations in force at the time, International Conference on Harmonisation Guidelines for Good Clinical Practices, and the Declaration of Helsinki.

Approved by:

[redacted] ______________________ Date (DD-MMM-YYYY)

[redacted]

[redacted] ______________________ Date (DD-MMM-YYYY)

[redacted]

PROTOCOL SYNOPSIS

Title	A Phase II, Randomized, Double-Blind, Vehicle Controlled Study of the Efficacy, Safety, and Tolerability of B244 Topical Spray for the Treatment of Pruritus in Adults with a History of Atopic Dermatitis
Protocol Number	PRB244-01
Sponsor	AOBiome Therapeutics
Development Phase	II
Study Objectives	Primary objective:<br>● To assess the efficacy of B244 in the treatment of pruritus in adults with a history of atopic dermatitis.<br>Secondary objectives:<br>● To assess the safety and tolerability of B244 in adults with a history of atopic dermatitis.
Study Design	This is a double-blind, randomized, vehicle-controlled study to assess the efficacy, safety, and tolerability of 2 doses of B244 for the treatment of pruritus in adults with a history of atopic dermatitis. Subjects who meet the study entry criteria will be randomized in a 1:1:1 ratio to receive twice daily topical doses of B244 at $1x10^{10}$ cells/ml (O.D. 5.0), 4 $x10^{10}$ cells/ml (O.D. 20.0) or vehicle (control) for 4 weeks. The total duration of the study will be approximately 11 weeks. The study will be conducted at approximately 50 study sites.<br>At Screening and Baseline, all subjects must have atopic dermatitis, which involves a minimum of 10% and a maximum of 40% body surface area, score of ≥ 7 points on the NRS scale and static IGA of 2-3.<br>The total duration of the study will be approximately 11 weeks. Participants will report for a Screening visit and if all inclusion/exclusion criteria are met, subjects will go through a two-week washout phase before reporting for a Baseline visit. At the baseline visit, eligible subjects will be randomly assigned to receive B244 $1x10^{10}$ cells/ml, B244 $4x10^{10}$ cells/ml or vehicle. Subjects will come in for Week 2 and Week 4 visits. After completion of the 4-week treatment period, all subjects will enter a 4-week follow-up period. Participants

	who have discontinued the study early will be evaluated by the Investigator at the Early Termination Visit within 7 days after their last dose of study drug.<br><br>The primary efficacy endpoint will be assessed after Week 4 of treatment.
Number of patients planned for randomization	Approximately 576 subjects (192 per treatment group) will be enrolled and randomized in the study.
Study Population	Male and female adults 18 to 65 years of age with pruritus and a history of atopic dermatitis.
Inclusion Criteria:	1. Male and female subjects 18 to 65 years of age.<br>2. Pruritus of at least 4 weeks duration prior to the initial Screening visit and during the 2 week washout period.<br>a. Subjects using stable doses of oral H1 antihistamines at the initial Screening visit must be willing to continue these at the same doses and frequencies throughout the study inclusive of the follow-up period.<br>3. Worst Itch Numeric Rating Scale (WI-NRS) score ≥ 7 in the 24-hour period prior to the initial Screening as well as Baseline visits.<br>4. Average weekly WI-NRS score ≥6 for each week of the washout period, as recorded in the eDiary.<br>5. A history of atopic dermatitis for greater than 12 months consistent with a diagnosis of atopic dermatitis, as defined by the 2014 American Academy of Dermatology (AAD) Guidelines of Care for the Management of Atopic Dermatitis.[1]<br>a. Subjects using bland emollients at the initial Screening visit will be allowed to continue to use their emollient of choice at the same dose and frequency throughout the study.<br>b. Subjects using low- to mid-potency topical corticosteroids at the initial Screening visit will be allowed to use their topical corticosteroid of choice at the same dose and frequency no more than 7 days per month throughout the study as rescue medication.<br>6. A minimum of 10% and not more than 40% of the subjects' BSA affected by atopic dermatitis (*affected* is defined by physical examination findings: erythema, edema, scaling, lichenification,

	excoriation, with the excoriation serving as the physical examination correlate of pruritus) at Screening and Baseline.<br>a. Subjects' BSA can include face and body OR body alone BUT NOT face alone.<br>7. An Investigator Global Assessment (IGA) score of 2-3 at Screening and Baseline.<br>8. Willing and able to complete once-daily eDiary entries within a consistent timeframe for the duration of the study and have ≥80% eDiary compliance rate during the washout period.<br>9. Judged to be in good health in the investigator's opinion.
Exclusion Criteria:	1. Clearly defined etiology for pruritus other than atopic dermatitis. These include but are not limited to urticaria, psoriasis or other non-atopic dermatologic conditions, hepatic or renal disease, psychogenic pruritus, drug reaction, untreated hyperthyroidism, parasite presence and presence of acute infection either systemically or in the AD lesions.<br>2. Presence of any acute condition which may risk inducing an atopic dermatitis flare during the course of the study, such as impetigo or active herpes simplex infection.<br>3. Treatment with systemic corticosteroids within 4 weeks prior to randomization.<br>4. Treatment with Class III or higher potency topical corticosteroids or any topical anti-pruritic therapies (other than stable doses of low- or mid-potency topical corticosteroids or bland emollients) within 4 weeks prior to randomization.<br>5. Treatment with systemic therapies with recognized anti-pruritic (e.g. tricyclic antidepressants, sedatives, tranquilizers, gabapentin, marijuana or other cannabinoids, opioid receptor agonists/antagonists) or pruritic (e.g. opioids, angiotensin-converting enzyme inhibitors, cocaine, antimalarials) properties within 4 weeks prior to randomization.<br>a. Stable doses of H1 antihistamines will be permitted. Subjects must be willing to continue these at the same doses and frequencies throughout the study inclusive of the follow-up period.<br>6. Any clinically significant changes in type, dose, or frequency of bland emollients, low- or mid-potency corticosteroids, and/or oral H1 antihistamines throughout the study from screening to follow-up.<br>7. Treatment with systemic immunosuppressive/ immunomodulatory therapies within 4 weeks prior to randomization (including but not

	limited to phosphodiesterase-4 inhibitors, cyclosporine, mycophenolate-mofetil, methotrexate, azathioprine, interferon-gamma, or phototherapy).<br>8. Treatment with biologic therapies within 12 weeks or 5 half-lives prior to randomization, whichever is longer.<br>9. Use of an indoor tanning facility within 4 weeks prior to randomization.<br>10. Treatment with any investigational therapy within 4 weeks prior to randomization.<br>11. Allergen immunotherapy within 6 months prior to randomization.<br>12. Prior use of AO+ Mist.<br>13. History of malignancy within 5 years prior to randomization, with the exception of completely treated and non-metastatic basal cell carcinoma or squamous cell carcinoma of the skin.<br>14. History of a major psychiatric condition (including major depressive disorder, bipolar disorder, or schizophrenia), suicidal ideation, or suicide attempt.<br>15. Known active hepatitis infection.<br>16. Known history of human immunodeficiency virus (HIV) infection.<br>17. Presence of any medical condition or disability that, in the investigator's opinion, could interfere with the assessment of safety or efficacy in this trial or compromise the safety of the subject.<br>18. Currently pregnant or breastfeeding, or male subject with a pregnant or breastfeeding partner.<br>19. Females of childbearing potential who are unable or unwilling to practice highly effective contraception (pregnancy prevention); fertile males who are unable or unwilling to use condoms with female partners of childbearing potential.
Study Drug	1) B244 $1x10^{10}$ cells/ml (O.D. 5.0)<br>2) B244 $4x10^{10}$ cells/ml (O.D. 20.0)<br>3) Vehicle
Dose Regimen	Subjects will apply 10 pumps of IP per application to all affected areas twice-a-day (i.e. 10 pumps in the morning and 10 pumps again at night) for 4 Weeks.
Efficacy Endpoints	The key primary efficacy endpoint is as follows:<br>● Mean change in WI-NRS from baseline to Week 4

	Additional secondary efficacy endpoints include the following:<br>● Proportion of subjects with ≥4 point improvement in WI-NRS from baseline to Week 4<br>● Proportion of subjects with any improvement in WI-NRS from baseline to Week 4<br>● Mean change in AI-NRS from baseline to Week 4<br>● Proportion of subjects with ≥4 point improvement in AI-NRS from baseline to Week 4<br>● Proportion of subjects with any improvement in AI-NRS from baseline to Week 4<br>● Mean change in WI-NRS from baseline to Week 2<br>● Proportion of subjects with ≥4 point improvement in WI-NRS from baseline to Week 2<br>● Mean change in POEM from baseline to Week 4<br>● Mean change in 5-D Pruritus Scale from baseline to Week 4<br><br>Exploratory endpoints include the following:<br>● Mean change in IGA from baseline to Week 4<br>● Mean change in EASI from baseline to Week 4
Safety Endpoints	Safety and tolerability endpoints include the following:<br>● Incidence of treatment-emergent adverse events (TEAEs) and serious adverse events (SAEs)<br>● Changes in vital signs and clinical laboratory parameters following study drug exposure<br>● Changes in local skin tolerability following application of study drug
Assessment Schedule:	All subjects will attend a screening visit not more than 21 days prior to Baseline (Day 0). Subjects will be required to return to the clinic at Baseline, Day 14 (Week 2) and Day 28 (Week 4). All subjects will be asked to attend a Week 8 follow-up visit 4 weeks (28 days (±3) days) after the last dose of study medication.
Statistical Considerations:	<u>Sample Size:</u><br>Approximately 576 subjects may be enrolled to account for 16.7% drop out rate prior to completing the study.<br>A total of 160 evaluable subjects per group (480 total) are required to achieve at least 80% to detect a pairwise difference of 0.65 in mean WI-NRS change from baseline to Week 4 between one of two active doses of B244 and vehicle control when assuming a standard deviation of 2.5 and applying a Dunnett

	Testing Method at a one-sided familywise error rate of 0.10.<br><br><u>Populations:</u><br><br>● ITT population: All subjects who are enrolled and apply at least 1 dose of study treatment will be included in the Intent to Treat (ITT) population. The ITT population will be the primary population for safety and efficacy assessments.<br><br>● Per Protocol (PP) population: All subjects in the ITT population without any protocol deviations that may have an impact on the efficacy assessments, who complete their Week 4 visit, and who administer at least 50% of investigational product (IP), will be included in the Per Protocol (PP) population.<br><br>● Safety Population: All subjects treated with at least one dose of IP.<br><br><u>Efficacy Analysis:</u><br><br>Efficacy analysis will be performed on the ITT population with some supportive analysis on the PP population. Regression models will include all eligible subjects for the respective analysis, from the 3 treatment arms.<br><br>Hypothesis tests for the primary efficacy endpoint will be performed using a Dunnett Testing Method, applying pairwise comparisons of each respective B244 dose group to vehicle using a one-sided familywise error rate of 0.10. No additional adjustments will be made for multiple testing.<br><br>Additional supportive analyses may be performed combining the B244 dose groups in comparison against vehicle and comparison of the B244 groups to one another.<br><br>The frequency and rate of NRS responders at Week 4 (≥4 point change from baseline in NRS) will be reported and compared between treatment groups using a logistic regression model.<br><br>Continuous measures of change from baseline to Week 1, Week 2, Week 3, Week 4, Week 8 for AI-NRS and WI-NRS; and change from baseline to Week 2, Week 4, Week 8 for POEM and 5-D Pruritus Scale will be analyzed using mixed models with repeated measures (MMRM) to account for within-subject variability over time. Comparisons at each time point will be performed within the longitudinal model.<br><br><u>Safety Analysis:</u>

	Analysis of safety measures will be performed on the Safety Population, including all treated subjects.<br><br>The incidence of all adverse events (AEs) and treatment-related AEs will be tabulated by treatment received. These AEs will be classified by system organ class and preferred term using the Medical Dictionary for Regulatory Activities (MedDRA). The incidence and severity of changes in local tolerability and adverse reactions will be collected as a solicited AE of special interest.<br><br>For incidence reporting, if a subject reported more than one AE that was coded to the same system organ class or preferred term, the subject will be counted only once for that specific system organ class or preferred term. An overview of AEs, which includes subject incidence of AEs, treatment-related AEs, AEs by severity, SAEs, deaths, and AEs leading to discontinuation, will be presented.<br><br>Summaries of IP exposure, clinical laboratory measures, vital signs, rescue medications, and concomitant medications will be presented.
Study Sites	Approximately 50 study sites
Expected Duration of Subject's Participation	Approximately 11 weeks: Up to 3 weeks of screening, 4 weeks of treatment, and a follow-up period of 4 weeks.

This study will be conducted in accordance with the Guidelines of Good Clinical Practices (GCPs).

Table of Contents

1 STUDY SCHEMA *17*

2 INTRODUCTION *18*

2.1 Background **18**

2.2 Clinical Experience **18**

2.3 Safety Profile **20**

2.4 Rationale for the Study **20**

3 STUDY OBJECTIVES *21*

3.1 Primary Objective **21**

3.2 Secondary Objectives **21**

4 ENDPOINTS *21*

4.1 Efficacy **21**

4.2 Safety & Tolerability **22**

5 STUDY DESIGN *22*

6 SELECTION OF STUDY PARTICIPANTS *23*

6.1 Number of Participants Planned **23**

6.2 Inclusion Criteria **23**

6.3 Exclusion Criteria **23**

7 PARTICIPANT ENROLLMENT *25*

7.1 Consenting Participants **25**

7.2 Screening for Eligibility **25**

7.3 Study Withdrawal and Withdrawal from Investigational Product and Stopping Criteria 25

7.4 Early Termination **26**

7.5 Lifestyle Considerations **27**

7.5.1 Use of Bland Emollients During the Study 27

8 STUDY TREATMENT *27*

8.1 Investigational Product **27**

8.2 Dose Changes **28**

8.3 Storage conditions **28**

8.4 Description of Blinding Method **29**

8.5 Treatment Assignments: ... 29

8.6 Treatment Compliance ... 29

8.7 Treatment Application ... 30

8.8 Treatment of Investigational Product Overdose ... 31

8.9 Study Drug Discontinuation ... 31

8.10 Product Accountability ... 32

8.11 Unblinding Procedures ... 32

8.12 Retrieval and Destruction of Investigational Product ... 32

8.13 Concomitant and Excluded Therapies ... 32

8.13.1 Permitted Medications ... 33

8.13.2 Excluded Therapies ... 34

8.14 Rescue Medications ... 34

8.14.1 Rescue Medication Permitted During the Study ... 34

8.14.2 Use of Excluded Therapies During the Treatment Period ... 35

8.15 Handling of Investigational Product ... 36

9 CONTRACEPTION REQUIREMENTS ... 36

10 STUDY PROCEDURES ... 37

10.1 Pre-screening procedures ... 37

10.2 Informed Consent Procedures ... 37

10.3 Study Assessments ... 37

10.4 Inclusion procedures ... 37

10.5 Timing of patient's visits to the clinic ... 38

10.6 Description by type of visit ... 38

10.6.1 Screening Visit (-21 to -14) ... 38

10.6.2 Washout Phase (-14 to -1) ... 38

10.6.3 Study Day 0-Baseline visit (-1 to 0) ... 39

10.6.4 Day 5- Phone call to patient ... 39

10.6.5 Day 14 and Day 28 study visits ... 40

10.6.6 Day 56-End of Study Visit ... 40

10.6.7 Unscheduled/Unanticipated Study visit ... 40

10.6.8 Early Termination Visit ... 41

11 STUDY ASSESSMENTS ... 41

11.1.1 Vital Signs ... 41

11.1.2 Physical Examinations ... 41

11.1.3 Laboratory Assessments ... 42

11.2 Efficacy Assessments ... 42

11.2.2 Patient Oriented Eczema Measure ... 43

11.2.3 Itch Numeric Rating Scale ... 43

11.2.4 Investigator Global Assessment Score (IGA) 44
11.2.5 5-D Pruritus Scale 44
11.3 eDiary and Efficacy Assessments 44
11.4 Patient Reported Local Tolerability 45
11.5 Pregnancy Reporting 45
11.6 Study Completion 45
11.7 Subject Withdrawal Criteria 45
12 STATISTICAL CONSIDERATIONS 46
12.1 Sample Size 46
12.2 Populations for Analysis 46
12.3 Data Analysis 46
12.3.1 Disposition 47
12.3.2 Demographic and Baseline 47
12.4 Safety Analyses 47
12.4.1 Definitions 47
12.4.2 Adverse Events 47
12.4.3 Deaths and Serious Adverse Events 48
12.4.4 Adverse Events leading to treatment discontinuation 48
12.5 Efficacy Analyses 48
12.6 Handling of dropouts or missing data 49
12.7 Clinical Trial Protocol deviations 49
13 ADVERSE EVENTS (AE) AND SERIOUS ADVERSE EVENTS (SAE) 49
13.1 Definition of an AE 49
13.2 Definition of a SAE 49
13.3 Time-period, Frequency, and Method of Detecting AEs and SAEs 50
13.4 Recording of AEs and SAEs 51
13.5 Evaluating AEs and SAEs 51
13.5.1 Severity Rating 51
13.5.2 Relationship to Investigational product (IP) 51
13.6 Pregnancy 52
13.7 Prompt Reporting of SAEs to the Sponsor 52
14 ETHICAL AND REGULATORY STANDARDS 53
14.1 Ethical Conduct of Study 53
14.2 Laws and Regulations 54
14.3 Informed Consent 54
14.4 Institutional Review Board/Independent Ethics Committee (IRB/EC) 55

14.5 Clinical Monitoring/Record Keeping **55**

15 ADMINISTRATIVE RULES ***56***

15.1 Curriculum Vitae **56**

15.2 Archiving of Study Documentation **56**

15.3 Internal Safety Review Committee **56**

16 STUDY MONITORING ***56***

16.1 Responsibilities of the Investigator(s) **56**

16.2 Responsibilities of the Sponsor **57**

16.3 Source document requirements **57**

16.4 Use and completion of Case Report Forms (CRFs) and additional requests **57**

17 PUBLICATIONS ***58***

18 PROTOCOL ADHERENCE ***59***

19 CLINICAL TRIAL PROTOCOL AMENDMENTS ***59***

20 CONFIDENTIALITY ***59***

21 PROPERTY RIGHTS ***59***

22 DATA PROTECTION ***60***

23 SPONSOR AUDITS AND INSPECTIONS BY REGULATORY AGENCIES ***60***

24 PREMATURE DISCONTINUATION OF THE STUDY OR PREMATURE CLOSE-OUT OF A SITE ***61***

24.1 Decided by the Sponsor in the following cases: **61**

24.2 Decided by the Investigator **61**

25 CLINICAL TRIAL PROTOCOL AMENDMENTS ***61***

26 REFERENCES ***62***

APPENDIX A: SCHEDULE OF EVENTS ***63***

APPENDIX B: EXCLUDED MEDICATIONS/THERAPY ***68***

APPENDIX C: EASI SCORE ***70***

APPENDIX D: PATIENT ORIENTED ECZEMA MEASURE (POEM) ***78***

APPENDIX E: IGA ***80***

APPENDIX F: 5-D PRURITUS SCALE ***81***

APPENDIX G: PATIENT REPORTED LOCAL TOLERABILITY ***82***

LIST OF ABBREVIATIONS AND DEFINITION OF TERMS

AD	Atopic Dermatitis
AE	Adverse Event
AI	Average Itch
AMO	Ammonia Monooxygenase
AOB	Ammonia Oxidizing Bacteria
BID	Twice-Daily
CRF	Case Report Form
EASI	Eczema Area and Severity Index
E/T	Early Termination
FDA	Food and Drug Administration
HAO	NH_2OH oxidoreductase
HbsAg	Hepatitis B Virus Surface Antigen
HCV Ab	Hepatitis C Virus Antibody
HIV	Human Immunodeficiency Virus
ICH	International Conference on Harmonization
IEC	Independent Ethics Committee
IGA	Investigator Global Assessment
IP	Investigational Product
IRB	Institutional Review Board
NH_2OH	Hydroxylamine
NH_3	Ammonia
NO	Nitric oxide
NO_2-	Nitrite
NRS	Itch Numeric Rating Scale
PCP	Primary Care Physician
POEM	Patient Oriented Eczema Measure
SAE	Serious Adverse Event
SPM	Study Procedures Manual
VAS	Visual Analog Scale
WI	Worst Itch

1 STUDY SCHEMA

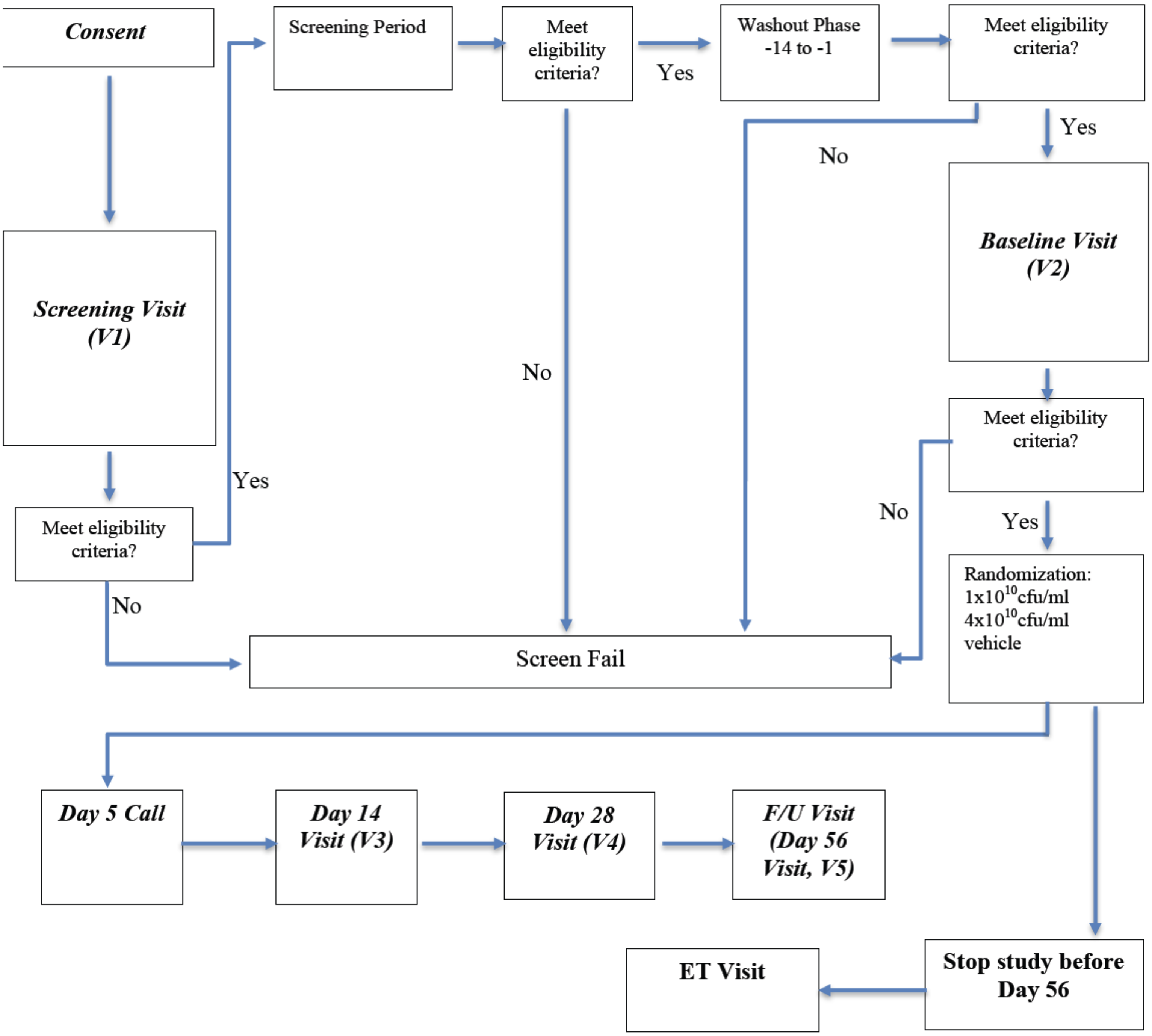

2 INTRODUCTION

2.1 Background

Ammonia oxidizing bacteria (AOB) are essential for the initial step in environmental nitrification processes, specifically the oxidation of ammonia (NH_3) to nitrite (NO_2-). *Nitrosomonas* are Gram-negative chemolithoautotrophic betaproteobacteria that obtain energy solely from NH_3 oxidation, while fixing CO_2 for their carbon needs[2]. Oxidation of NH_3 proceeds in two steps (Figure 1) leading to sequential generation of hydroxylamine (NH_2OH) and NO_2- that require two enzyme complexes: the membrane-bound ammonia monooxygenase (AMO) comprised of subunits AmoA, AmoB and AmoC; and the periplasmic NH_2OH oxidoreductase (HAO). In addition to high NO_2- levels, NH_3 oxidation leads to nitric oxide (NO) and N_2O production through two independent pathways downstream of NH_2OH production: nitrifier denitrification and NH_2OH oxidation[3].

Figure 1 Nitrifier Denitrification Pathway

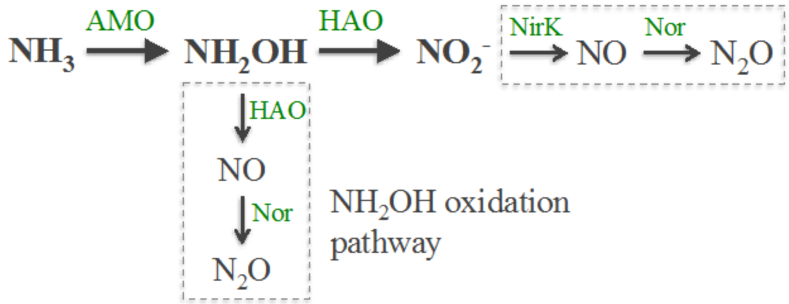

B244 is a purified a strain of *Nitrosomonas eutropha* originally isolated from soil samples. Sequencing of the B244 genome revealed a distinct genetic profile from that of other published *Nitrosomonas* strains and AOB genomes. Based on *in vitro* co-culture studies, B244 was able to reduce survival of pathogenic bacteria. Nitrite generation from ammonia concurrently with medium acidification by B244 led to strong antibacterial effects and a marked reduction (~100-fold) in viable counts of methicillin-resistant *Staphylococcus aureus* and *Pseudomonas aeruginosa*, two pathogens frequently isolated from infected skin and wound sites. In contrast, control cultures with B244 in the absence of ammonium or with heat-killed B244 supplemented with ammonium, had no antibacterial effects. The unique metabolic and antimicrobial activity of *Nitrosomonas*, in combination with their lack of virulence render these bacteria as attractive candidates for topical delivery of nitrite and nitric oxide on human skin with potential to improve health in both normal and abnormal skin conditions or wound sites. NO-releasing drugs or NO donors have also shown activity against *Propionibacterium acnes* and other pathogenic bacteria, anti-inflammatory activity, and inhibition of lipogenesis by insulin-stimulated immortal sebocytes[4-6].

2.2 Clinical Experience

B244 is being developed as a 'live topical' to provide a natural source of AOB and NO/NO_2 to the human skin.

AOBiome's first Phase 1b/2a clinical trial titled, "*A Double Blind, Vehicle-Controlled, Single Center, Randomized, Sequential, Ascending 14-Day Multiple Dose Study in Subjects with Acne Vulgaris to Evaluate the Safety, Tolerability and Preliminary Efficacy of B244 Delivered as a Topical Spray*" was completed in 2016 where 36 participants with clinical diagnosis of facial acne vulgaris were randomized to receive ascending doses of investigational product (IP) over 14 days. Safety analyses have been completed and there has been no attributable drug related SAEs reported. In addition, a Phase 2b/3 clinical trial titled, "*A Randomized, Double Blinded, Phase IIb/III, Decentralized Study of B244 Delivered as a Topical Spray to Determine Safety and Efficacy in Participants with Mild to Moderate Acne Vulgaris in 372 patients with clinical diagnosis of facial acne*" has been completed. B244 was safe and well tolerated with no attributable drug related SAEs. Efficacy was supported by statistically significant 2-point reduction in IGA with B244 and a trend in the reduction of the number of inflammatory lesions compared to vehicle.

A study titled, "*A Prospective, Vehicle Controlled, Double Blind, Multicenter, Randomized, Phase II Study of B244 Delivered as a Topical Spray to Determine Safety and Efficacy in Subjects with Mild to Moderate Atopic Dermatitis"* has recently been completed. A total of 122 subjects were enrolled and randomized in a 1:1 ratio to receive B244 or vehicle in the study. Overall, there were no unexpected safety signals observed following treatment with B244. A total of 30 (25%) subjects experienced at least 1 treatment-emergent adverse events (TEAE) during the study, including 16 (26%) subjects in the B244 treatment group and 14 (23%) subjects in the vehicle group.

AOBiome completed a Phase Ib study in pediatric and adolescent subjects aged 2-17 years of age. A study titled, *"An Open-label, Multicenter, Phase Ib Study of B244 Delivered as a Topical Spray to Assess Safety in Pediatric Subjects aged 2 to 17 years with Atopic Dermatitis"* enrolled a total of 28 subjects across 3 cohorts. Overall, there were no unexpected safety signals observed following treatment with B244 in pediatric subjects with atopic dermatitis. The B244 spray was well tolerated and not associated with any increased pain or redness at the application site.

"*A Vehicle-Controlled, Double-Blind, Randomized Phase II Study of B244 Delivered as a Topical Spray to Determine Safety and Efficacy in Subjects with Mild to Moderate Rosacea"* has been completed with a total of 140 subjects randomized. Generally, there were no unexpected safety signals observed following treatment with B244. Overall, 31 (22.1) subjects had experienced at least one treatment emergent adverse event of which 19 (26.0%) subjects in B244 spray group and 12 (17.9%) in the Vehicle group.

In addition, other topical development programs with B244 include hypertension. A study titled, "A Prospective, Controlled, Double Blinded, Multicenter, Randomized, Vehicle Controlled, Phase II Study of B244 Delivered as a Topical Spray to Determine Safety and Efficacy in Subjects with Elevated Blood Pressure" has been completed. Safety data indicated that B244 was safe and well tolerated. Overall, 16% of subjects experienced at least 1 TEAE during the study with comparable incidence in the B244 and Vehicle groups. Overall, there were no unexpected safety signals observed following treatment with B244.

Moreover, intranasal application of B244 is being developed for the treatment of allergic rhinitis and migraines. A rat toxicology study performed by intranasal administration of B244 twice daily for 28 days found that B244 was safe and well tolerated in rats at levels of up to the maximum dose $8x10^9$ cell/mL. A study titled, "*A Prospective, Controlled, Double Blinded, Single Center, Randomized, 3 arm, Phase 1b/2a Study to Assess the Safety, Tolerability, and Preliminary Efficacy of B244 Delivered as an Intranasal Spray in Healthy Volunteers and Subjects with Seasonal Allergic Rhinitis*" has been completed. In addition, a study titled "*A Prospective, Randomized, Vehicle-Controlled, Double-Blind, Phase 2 Study to Assess the Safety, Tolerability, and Efficacy of B244 Delivered as an Intranasal Spray for Preventive Treatment in Subjects with Episodic Migraine*" is in progress.

2.3 Safety Profile

To date, there have been no reported infections or health risks associated with topical application or ingestion of *Nitrosomonas* species. The absence of any illnesses attributed to these bacteria despite our widespread exposure indicates that they pose a minimal health risk, if any at all. Infection or tissue damage by *Nitrosomonas* is unlikely, because the sequenced genomes of several *Nitrosomonas* and other AOB lack genes encoding cytotoxins, or other known bacterial virulence factors. Further, AOB are slow growing, as compared to most heterotrophic bacteria, with optimum doubling times of 8 hours or higher. In particular, *Nitrosomonas* growth is rate limited by the availability of ammonia requiring the oxidation of 27 moles NH_3/mole CO_2 fixed. Due to their dependence on ammonia for their growth, the numbers of *Nitrosomonas* on the skin will be necessarily limited and naturally regulated by the amount of ammonia produced in sweat. This would ensure that the amount of nitrite and NO generated would be relatively low, without any adverse effects.

2.4 Rationale for the Study

Eczema or Atopic dermatitis is an inflammatory skin condition that affects nearly 20% of children and between 2-10% of adults[7]. Disease prevalence has steadily grown in the last 30 years, resulting in a growing field of atopic dermatitis research[8].

Pruritus is a common morbidity associated with Atopic Dermatitis, and its management remains a challenge for physicians. Pruritus is defined as an urge or unpleasant sensation that produces the urge to scratch[9]. Modern treatment no longer consists of antihistamines alone. Anti-inflammatory, antiseptic, as well as antipruritic therapies to address the epidermal barrier as well as immunomodulation or infection are now used in clinical practice[9]. Mild pruritus may be controlled with topical therapies, while moderate to severe pruritus requires combination treatment, consisting of antipruritic and immunosuppressive drugs, phototherapy, and topical compounds[9].

While the complete pathophysiology of pruritus remains unclear, histamines are one of the most potent mediators in AD related pruritus. The itch cycle exacerbates damage to the epidermal barrier leading to water loss and dryness, thereby creating a hospitable environment for skin

pathogens, which cause infections and flare up symptoms. *Staphylococcus aureus* is consistently found in eczematous skin lesions in patients with AD. Correlation between the severity of the disease and presence of *Staphylococcus aureus* has been well established and it has been shown that the presence of bacteria is an important factor in skin aggravation[10]. The goal of therapy for AD is to restore the epidermal barrier function and reduce skin inflammation, thereby alleviating symptoms and intensity of AD associated pruritus. However, systemic antibiotic use is controversial.

B244 has been developed as a topical application of a natural source of AOB and NO/NOx to the human skin. We hypothesize that application of AOB to eczematous skin may reduce *Staphylococcus aureus* skin load and normalize the inflammatory response by reducing Th2 activation.

The primary purpose of this study is to evaluate the efficacy and safety of B244 in the treatment of pruritus in adults with a history of atopic dermatitis.

3 STUDY OBJECTIVES

3.1 Primary Objective

- To assess the efficacy of B244 in the treatment of pruritus in adults with a history of atopic dermatitis.

3.2 Secondary Objectives

- To assess the safety and tolerability of B244 in adults with a history of atopic dermatitis.

4 ENDPOINTS

4.1 Efficacy

4.1.1 Primary

- Mean change in WI-NRS from baseline to Week 4

4.1.2 Secondary

- Proportion of patients with ≥4 point improvement in WI-NRS from baseline to Week 4
- Proportion of subjects with any improvement in WI-NRS from baseline to Week 4
- Mean change in AI-NRS from baseline to Week 4
- Proportion of subjects with ≥4 point improvement in AI-NRS from baseline to Week 4
- Proportion of subjects with any improvement in AI-NRS from baseline to Week 4
- Mean change in WI-NRS from baseline to Week 2
- Proportion of subjects with ≥4 point improvement in WI-NRS from baseline to Week 2
- Mean change in POEM from baseline to Week 4
- Mean change in 5-D Pruritus Scale from baseline to Week 4

4.1.3 Exploratory

- Mean change in IGA from baseline to Week 4
- Mean change in EASI from baseline to Week 4

4.2 Safety & Tolerability

Safety and tolerability endpoints include the following:

- Incidence of TEAEs and SAEs
- Changes in vital signs and clinical laboratory parameters following study drug exposure
- Changes in local skin tolerability following application of study drug

5 STUDY DESIGN

- This is a Prospective, Vehicle Controlled, Double-Blind, Multicenter, Randomized Phase II Trial, comparing the effect of twice daily B244 applications for 4 weeks vs vehicle applications on treatment of mild to moderate pruritus associated with atopic dermatitis.
- Approximately 576 subjects may be enrolled.
- The total duration of the study will be approximately 11 weeks. Participants will report for a Screening visit and if all inclusion/exclusion criteria are met, subjects will go through a two-week washout phase (see Section 10.6.2) before reporting for a Baseline visit.
- After screening and baseline, participants will be randomized to one of two doses of B244 or vehicle application for 4 weeks.
- Randomization will be 1:1:1 so that an equal number of patients will be treated in each Arm of the study.
- All B244 randomized subjects will be treated at the dose of $1x10^{10}$ cells/ml (O.D. 5.0) or $4x10^{10}$ cells/ml (O.D. 20.0).
- Subjects must be willing and able to complete diary within a consistent time frame on a daily basis and to comply with restrictions on allowable therapies for the duration of the study.
- All subjects will attend a screening visit not more than 21 days prior to Baseline (Day 0). Subjects will be required to return to the clinic at Baseline, Day 14 (Week 2) and Day 28 (Week 4) visits. All subjects will be asked to attend a Week 8 follow-up visit 4 weeks (28 (±3) days) after the last dose of study medication.
- Subjects will apply a total of 10 pumps of IP per application across all affected areas twice-a-day (i.e. 10 pumps in the morning and 10 pumps again at night) for 4 weeks.
- Safety evaluations will consist of review of participant's medical history at screening and on-going assessment of adverse events reported throughout the study duration.

6 SELECTION OF STUDY PARTICIPANTS

6.1 Number of Participants Planned

Approximately 576 subjects (192 per treatment group) with pruritus and a history of atopic dermatitis will be randomized into this study.

6.2 Inclusion Criteria

Deviations from inclusion criteria are not allowed because they can potentially jeopardize the scientific integrity of the study, regulatory acceptability or participant safety. Therefore, adherence to the criteria as specified in the protocol is essential.

Participants eligible for enrollment in the study must meet all the following criteria:

1. Male and female subjects 18 to 65 years of age.
2. Pruritus of at least 4 weeks duration prior to the initial Screening visit and during the 2 week washout period.
    a. Subjects using stable doses of oral H1 antihistamines at the initial Screening visit must be willing to continue these at the same doses and frequencies throughout the study inclusive of the follow-up period.
3. Worst Itch Numeric Rating Scale (WI-NRS) score ≥ 7 in the 24-hour period prior to the initial Screening as well as Baseline visits.
4. Average weekly WI-NRS score ≥6 for each week of the washout period, as recorded in the eDiary.
5. A history of atopic dermatitis for greater than 12 months consistent with a diagnosis of atopic dermatitis, as defined by the 2014 American Academy of Dermatology (AAD) Guidelines of Care for the Management of Atopic Dermatitis[1].
    a. Subjects using bland emollients at the initial Screening visit will be allowed to continue to use their emollient of choice at a similar dose and frequency throughout the study, if used.
    b. Subjects using low- to mid-potency topical corticosteroids at the initial Screening visit will be allowed to use their topical corticosteroid of choice at the same dose and frequency no more than 7 days per month throughout the study as rescue medication.
6. A minimum of 10% and not more than 40% of the subjects' BSA affected by atopic dermatitis (*affected* is defined by physical examination findings: erythema, edema, scaling, lichenification, excoriation, with the excoriation serving as the physical examination correlate of pruritus) at Screening and Baseline.
    a. Subjects' BSA can include face and body OR body alone BUT NOT face alone.
7. An Investigator Global Assessment (IGA) score of 2-3 at Screening and Baseline.
8. Willing and able to complete once-daily eDiary entries within a consistent timeframe for the duration of the study and have ≥80% eDiary compliance rate during the washout period.
9. Judged to be in good health in the investigator's opinion.

6.3 Exclusion Criteria

Deviations from exclusion criteria are not allowed because they can potentially jeopardize the scientific integrity of the study, regulatory acceptability or participant safety. Therefore, adherence to the criteria as specified in the protocol is essential.

Participants will be excluded from the study if any of the following criteria are met:

1. Clearly defined etiology for pruritus other than atopic dermatitis. These include but are not limited to urticaria, psoriasis or other non-atopic dermatologic conditions, hepatic or renal disease, psychogenic pruritus, drug reaction, untreated hyperthyroidism, parasite presence and presence of acute infection either systemically or in the AD lesions.
2. Presence of any acute condition which may risk inducing an atopic dermatitis flare during the course of the study, such as impetigo or active herpes simplex infection.
3. Treatment with systemic corticosteroids within 4 weeks prior to randomization.
4. Treatment with Class III or higher potency topical corticosteroids or any topical anti-pruritic therapies (other than stable doses of low- or mid-potency topical corticosteroids or bland emollients) within 4 weeks prior to randomization.
5. Treatment with systemic therapies with recognized anti-pruritic (e.g. tricyclic antidepressants, sedatives, tranquilizers, gabapentin, marijuana or other cannabinoids, opioid receptor agonists/antagonists) or pruritic (e.g. opioids, angiotensin-converting enzyme inhibitors, cocaine, antimalarials) properties within 4 weeks prior to randomization.
    a. Stable doses of H1 antihistamines will be permitted. Subjects must be willing to continue these at the same doses and frequencies throughout the study inclusive of the follow-up period.
6. Any clinically significant changes in type, dose, or frequency of bland emollients, low- or mid-potency corticosteroids, and/or oral H1 antihistamines throughout the study from screening to follow-up.
7. Treatment with systemic immunosuppressive/ immunomodulatory therapies within 4 weeks prior to randomization (including but not limited to phosphodiesterase-4 inhibitors, cyclosporine, mycophenolate-mofetil, methotrexate, azathioprine, interferon-gamma, or phototherapy).
8. Treatment with biologic therapies within 12 weeks or 5 half-lives prior to randomization, whichever is longer.
9. Use of an indoor tanning facility within 4 weeks prior to randomization.
10. Treatment with any investigational therapy within 4 weeks prior to randomization.
11. Allergen immunotherapy within 6 months prior to randomization.
12. Prior use of AO+ Mist.
13. History of malignancy within 5 years prior to randomization, with the exception of completely treated and non-metastatic basal cell carcinoma or squamous cell carcinoma of the skin.
14. History of a major psychiatric condition (including major depressive disorder, bipolar disorder, or schizophrenia), suicidal ideation, or suicide attempt.
15. Known active hepatitis infection.
16. Known history of human immunodeficiency virus (HIV) infection.

17. Presence of any medical condition or disability that, in the investigator's opinion, could interfere with the assessment of safety or efficacy in this trial or compromise the safety of the subject.
18. Currently pregnant or breastfeeding, or male subject with a pregnant or breastfeeding partner.
19. Females of childbearing potential who are unable or unwilling to practice highly effective contraception (pregnancy prevention); fertile males who are unable or unwilling to use condoms with female partners of childbearing potential.

7 PARTICIPANT ENROLLMENT

7.1 Consenting Participants

Informed consent for participation in the study must be obtained before performing any study-specific procedures.

Informed Consent Forms for enrolled participants and for participants who are not subsequently enrolled will be maintained at the study site in secure study files. Consent will be obtained by trained research study staff trained in taking informed consent. The study will be explained with the opportunity for the participant to ask questions. If a participant wishes to enter the study, a consent form will be completed and signed.

7.2 Screening for Eligibility

After informed consent has been obtained, to determine participant eligibility for enrollment in the study, screening assessments will be performed within 1 week (-21 to -14 days) prior to starting the Washout period (-14 to -1). All screening evaluations must be completed and reviewed to confirm that participants meet all eligibility criteria before Washout period and subsequent randomization on Day 0. Subjects will be asked to undergo a 2 week (-14 to -1) washout period prior to the Baseline procedure. During the screening, washout, treatment and follow-up periods, subjects will be asked to stop utilizing bleach or vinegar as a pruritus or atopic dermatitis therapy, as well as any other treatments described in the exclusion criteria and the Excluded Medications/Therapy (Appendix B).

All screening assessments are listed in the Time and Events Table (Appendix A). A participant must meet all inclusion criteria, and none of the exclusion criteria, to be enrolled and randomized in this study. The Investigator and team will maintain a screening log to record details of all persons screened and to confirm eligibility or record reasons for screening failure, as applicable.

7.3 Study Withdrawal and Withdrawal from Investigational Product and Stopping Criteria

A participant may withdraw from the study at any time at his/her own request, or may be withdrawn at any time at the discretion of the Investigator.
Reasons for withdrawal (participants who refuse to complete any remaining study visits) or discontinuation (participants who prematurely stop the application) at any time during the study may include, but are not limited, to the following:

- For safety reasons, either at the discretion of the Investigator or at the participant's request
- For protocol violations at the discretion of AOBiome
- Withdrawal of consent by the Subject
- Lost to follow-up
- Due to use of concomitant therapy that could interfere with the results of the study (the Investigator will report all such information through the CRF and decide, in accordance with AOBiome, whether the participant is to be withdrawn).

The reason for participant study withdrawal will be recorded in the electronic Case Report Form (eCRF). Data from participants withdrawing from the study will be considered evaluable up to the point at which they are withdrawn using the same criteria for evaluability as for participants who complete the study.

Study stopping rules will be implemented to stop the study for safety review in the event that:

- 1 subject reports death, or
- >2 subjects report an SAE, or
- >4 subjects experience grade 3 AEs of a similar type, or
- >6 subjects experience grade 2 AEs of a similar type

when the reported SAEs or AEs are considered possibly, probably, definitely or related to the investigational product.

Atopic Dermatitis is an active disease with known seasonal and personal histories of flares and exacerbations of the patient's underlying disease state. This includes personal histories of worsening itch and itch-scratch cycles, which can lead to localized infection and localized cellulitis. A subject's known and collected clinical history should be considered by the principal investigator when determining relationship of event to the investigational product.

7.4 Early Termination

Early termination from the study may occur due to loss to follow-up or withdrawal of consent by the subject. Participants who have discontinued the study early will be evaluated by the Investigator at the Early Termination Visit. See the list of assessments to be performed at the Early Termination Visit in the Time and Events Table (Appendix A). Participants with ongoing AEs or SAEs believed to be possibly related to investigational product (IP) will continue to be followed until resolution or for 30 days as warranted by the nature of the AE.

7.5 Lifestyle Considerations

There are no dietary or activity related restrictions for this study.

7.5.1 Use of Bland Emollients During the Study

Subjects will be allowed to use their usual choice of bland emollients (e.g., lotion, moisturizer) from screening to follow-up, on an as needed basis, using specific time of use guidelines in relation to study medication application. Subjects will be allowed to use their emollient of choice at a similar dose and frequency throughout the study if used. Any clinically significant changes in type, dose, or frequency of bland emollients will not be allowed during the study. A detailed guideline will be shared with the sites and subjects to ensure consistency in study medication treatment application and relation to body washing, emollient use, and other chemical exposure to skin.

Subjects will be encouraged not to use sunscreen on the treated lesions during participation in the study. Sunscreen should be used minimally or used sparingly while in the active treatment phase (Baseline to Week 4) of the study. Alternatively, sunscreen should be washed off prior to study medication use.

Subjects who do not have atopic dermatitis on their face may apply makeup as needed. However, those subjects who have atopic dermatitis on their face and require to use makeup will be advised to use it minimally.

8 STUDY TREATMENT

8.1 Investigational Product

Under normal conditions of handling and administration, IP is not expected to pose significant safety risks to site staff. A Material Safety Data Sheet describing the occupational hazards and recommended handling precautions will be provided to site staff if required by local laws or will otherwise be available from the Sponsor upon request.

Investigational product must be stored in a secure area under the appropriate physical conditions for the product (2-8°C). Access to and administration of the IP will be limited to the Investigator and authorized site staff. Investigational product must be dispensed or administered only to participants enrolled in the study and in accordance with this protocol (see Section 8.6).

The Investigator is responsible for study treatment accountability, reconciliation, and record maintenance. The Investigator or designated site personnel must maintain study treatment accountability records throughout the course of the study. The responsible person(s) will document the amount of study treatment received from and returned to the sponsor and the amount administered to participants. The required accountability unit for this study will be the

bottle. Discrepancies are to be reconciled or resolved.

**Product name:**	B244, 30ml/bottle	B244, 30ml/bottle	Vehicle, 30ml/bottle
**Dosage form:**	B244 suspension	B244 suspension	Vehicle solution
**Unit dose strength:**	1 x 10^{10} cells/ml (O.D. 5.0)	4x10^{10} cells/ml (O.D. 20.0)	50nM Na_2HPO_4-2mM $MgCl_2$ (pH 7.6)
**Route/administration/duration:**	Topical application BID for 28 days	Topical application BID for 28 days	Topical application BID for 28 days
**Dosing instructions:**	Subjects will apply a total of 10 pumps of IP per application to all affected areas twice-a-day (i.e., 10 pumps in the morning and 10 pumps again at night).<br><br>Applications should occur in the morning and at night for 4 weeks.	Subjects will apply a total of 10 pumps of IP per application to all affected areas twice-a-day (i.e., 10 pumps in the morning and 10 pumps again at night).<br><br>Applications should occur in the morning and at night for 4 weeks.	Subjects will apply a total of 10 pumps of IP per application to all affected areas twice-a-day (i.e., 10 pumps in the morning and 10 pumps again at night).<br><br>Applications should occur in the morning and at night for 4 weeks.
**Physical description:**	Odorless, cloudy, light pink suspension	Odorless, cloudy, light pink suspension	Odorless, clear, and colorless suspension
**Manufacturer/source of procurement:**	AOBiome, LLC	AOBiome, LLC	AOBiome, LLC

The contents of the label will be in accordance with all applicable regulatory requirements. B244 and matching vehicle will be packaged in identical 30 ml white bottles.

8.2 Dose Changes

No dose changes are anticipated.

8.3 Storage conditions

All investigational drug supplies in the study will be stored in a secure, refrigerated (2-8°C) safe place, under the responsibility of the Investigator or other authorized individual.

8.4 Description of Blinding Method

This study will be double-blinded: neither Investigator(s), nor study participants, nor those involved in the conduct of the trial (including sponsor staff) will be aware of the treatment the participants are receiving. The appearance of B244 and vehicle dispensing containers will be identical.

8.5 Treatment Assignments:

This is a double-blind study. Participants will be assigned to study treatment in accordance with the randomization schedule generated for the allocation of B244 or vehicle prior to the initiation of the trial. Randomization will be centrally-based and performed using an appropriate IWRS (an automated randomization system).

Each participant scheduled to receive investigational product (IP) will receive a randomization number at the time of randomization. The randomization number will be used to identify the study medication kit assigned to the participant and indicate the treatment to be administered to that participant.

8.6 Treatment Compliance

Study treatment must be dispensed or administered according to procedures described herein. Only participants enrolled in the study may receive study treatment. Only authorized site personnel may supply study treatment. Participants will record use of the study medication utilizing the study diary at the time of use each day. Participants will review study medication compliance with the Investigator or designee. Any missed doses, timing, and reason for missed dose will be recorded in the eCRF. There should be no doubling of doses to make up for missed doses. If a dose is missed, the next dose of study medication should be taken as scheduled.

Each site participating in the trial will be instructed to assess subject's compliance by weighing the investigational product at the Baseline visit, Week 2 visit, and Week 4 visit and followed every time study medication is dispensed and returned per instructions below. Sites will be provided scales, which will be calibrated prior to each use. Study personnel will be instructed to record measurements into the eCRF.

All weight measurements will be performed without the safety cap. Subjects will be dispensed 2 bottles upon randomization, and each bottle will be labeled 1 and 2 with a permanent marker at the site. Unused Bottle 1 will be vortexed, primed, and weighed without the cap by the site per treatment application instructions and dispensed to the subject who will mix and apply the first dose at the site under study staff supervision. Bottle 2 will also be vortexed, primed, and weighed at the site at the time of dispense without the cap, the cap inserted back on the bottle,

and the bottle returned to its carton unused. After the first treatment application of Bottle 1, subjects will take home both bottles where they will place Bottle 1 on the counter at room temperature for in-home application twice a day until empty. Bottle 2 will be stored in the refrigerator until Bottle 1 is empty, at which point Bottle 2 will be taken out of the refrigerator, mixed per treatment application instructions, and used. Should Bottle 1 run out during a treatment application, the subject will count the number of pumps that were made with Bottle 1 and complete the remaining number of pumps needed to achieve a total of 10 pumps using Bottle 2.

At the Week 2 visit, subjects will return any empty bottle (likely Bottle 1 since each bottle should last approximately 10 days and the subject should be on Bottle 2 at the time of visit) to the site for weight measurement (without the safety cap) to confirm usage. At this visit, a third bottle of the same treatment group will be labeled as Bottle 3 and will be vortexed, primed, and weighed at the site at the time of dispense without the cap, the cap inserted back on the bottle, and the bottle returned to its carton unused. The subject will be dispensed Bottle 3 to take home and store in the refrigerator until Bottle 2 is empty, at which point Bottle 3 is taken out of the refrigerator and used on the counter until the Week 4 visit. All empty bottles returned to the site will be collected and stored by the site in ambient temperature until return to the drug depot. If the subject is still on Bottle 1 at the Week 2 visit, the site will confirm daily dosing application and retrain the subject on the appropriate use of IP application, and the subject will continue using Bottle 1 until empty, at which point Bottle 2 is taken out of the refrigerator and used on the counter until Bottle 2 is empty, after which Bottle 3 is taken out of the refrigerator and used on the counter until the Week 4 visit. Any bottle currently in use at the time of the Week 2 visit should not be returned to the site.

At the Week 4 visit, all used and unused bottles will be returned to the site for weight measurements and storage at the site in ambient temperature until return to the drug depot.

8.7 Treatment Application

Subjects will receive study drug for application throughout the study. Subjects will be instructed in the use of the spray bottle and asked to self-administer the Investigational Product following a detailed instruction on treatment application that will be shared with the sites and subjects to ensure consistency in treatment application and relation to body washing, emollient use, and other chemical exposure to skin. Key guidelines include:

- The subject should wash his/her hands before applying study treatment.
- Subjects will apply a total of 10 pumps of IP per application to all affected. areas twice-a-day (i.e., 10 pumps in the morning and 10 pumps again at night).
- The subject will apply the IP to the affected area twice daily (approximately 12 hours apart) for 28 consecutive days.
- Subject should mix the bottle thoroughly per instructions provided before each application and then saturate the application area well, holding the bottle approximately 6 inches away from the skin.

- Subjects will be asked to let the product air dry after each application.
- Subjects will follow instructions provided for body washing, emollient use, and other chemical exposure to skin in relation to timing of IP application.
- The spray bottle in use may be stored at ambient temperature until used up, while additional unused bottles will be stored in the refrigerator until use. DO NOT FREEZE.
- The subject will apply the IP to the worst and largest lesions first and then apply the IP to the smaller lesions. If after covering all lesions, the total number of pumps per application has not been exhausted, subject should go back and cover the worst lesions with the remaining number of pumps. However, the total number of 10 pumps allowed should not be exceeded per application.
- Subjects will be asked not to expose the treatment kit to conditions which are unnatural or harmful to the product, such as excessive heat (temperatures over 77°F [25°C] and freezing temperatures below 32°F [0°C]). Subjects may travel with their study medication but should not leave it in a hot car, outside in the cold temperatures, etc. Subjects will also be asked not to tamper or cause damage to the IP bottle.

8.8 Treatment of Investigational Product Overdose

The sponsor does not recommend specific treatment for an overdose. Washing with conventional cleanser and water will remove the product. The Investigator will use clinical judgment to treat any overdose.

8.9 Study Drug Discontinuation

Subjects will be discontinued from study drug treatment in the following events:

- The subject experiences a Grade 2 or higher treatment emergent AE that is assessed as likely related to study drug
- The subject receives treatment with an excluded therapy or has a clinically significant change in dose or frequency of allowed adjunctive therapies during the treatment period
- The female subject becomes pregnant or female partner of a male subject becomes pregnant or is breastfeeding

Discontinuation from study drug treatment may also occur for any of the following reasons:

- Subject decision to discontinue study drug treatment, or subject decision to withdraw consent from the study
- Any medical condition that may jeopardize the subject's safety if study drug is continued, in the investigator's and/or Sponsor's opinion
- Discontinuation is deemed to be in the best interest of the subject, in the investigator's and/or Sponsor's opinion

Subjects who discontinue treatment with study drug prior to completing the treatment period will have an ETD visit within 7 days after their last dose of study drug in addition to a follow-up visit.

8.10 Product Accountability

In accordance with federal and local regulatory requirements, the Investigator and designated site personnel must document the amount of investigational product dispensed to study participants, the amount returned by study participants, and amount received and returned to the sponsor, when applicable. Product accountability records must be maintained throughout the course of the trial. Any quality issue noticed with the receipt or use of an IP (deficient IP in condition, appearance, pertaining documentation, labeling, expiry date, etc.) should be promptly reported to the Sponsor, who will initiate a complaint procedure. All investigational product must be stored in a secure locked room with access limited to the Investigator and designated site personnel. Study product is to be stored in a refrigerator between 2-8°C. Maintenance of a temperature log is required. Under no circumstances will the Investigator allow IP to be used other than as directed by this Clinical Trial Protocol, or dispose of IP in any other manner.

8.11 Unblinding Procedures

The Investigator may unblind a participant's treatment assignment only in the case of emergency or in the event of a serious medical condition when knowledge of the study treatment is essential for the appropriate clinical management or welfare of the participant, as determined by the Investigator. It is preferred (but not required) that the Investigator first contacts the Medical Monitor to discuss options before unblinding the participant's treatment assignment. The Investigator must notify the Sponsor as soon as possible when a participant's treatment assignment is unblinded without revealing the treatment assignment of the unblinded participant unless that information is deemed important for the safety of participants currently in the study. The date and reason for the unblinding must be documented in the participant's study record. The Medical Monitor may unblind the treatment assignment for any participant with an SAE. If the SAE requires that an expedited regulatory report be sent to one or more regulatory agencies, a copy of the report, identifying the participant's treatment assignment, may be sent to clinical Investigators in accordance with local regulations and/or sponsor policy.

8.12 Retrieval and Destruction of Investigational Product

All partially used or unused treatments will be returned to the site as brought by study participants. A detailed IP log of the returned IP shall be established.

The site will not destroy unused IP unless the Sponsor provides written authorization to the contrary. All used and unused bottles will be shipped to the authorized drug depot at the end of the study.

8.13 Concomitant and Excluded Therapies

Any medication or vaccine (including over-the-counter or prescription medicines, vitamins, and/or herbal supplements) that the subject is receiving within 30 days prior to screening and through the final study visit will be recorded on the appropriate CRF) along with:

- Reason for use.
- Dates of administration including start and end dates.
- Dosage information including dose and frequency.

Any medications started during the study (including "as needed" medications) will be recorded in the concomitant medication list as soon as the Investigational Site will become aware of the medication being added.

Details of any medication taken by the subject outside of the study center will be reviewed by the Investigator or designee on each study center visit. The Medical Monitor should be contacted if there are any questions regarding concomitant or prior therapy.

A list of excluded medications/therapy is provided in Appendix B.

Previous treatment of atopic dermatitis must be recorded irrespective of the term it was given. Acetaminophen is permitted for use any time during the study. Other concomitant medication may be considered on a case-by-case basis by the Investigator in consultation with the Medical Monitor.

Concomitant therapies include any therapies (including over-the-counter medications) used by a subject from initiation of study drug treatment through the follow-up period. A record of all medications used will be maintained for each subject throughout the study. Reported information will include a description of the type of drug, treatment period, dosing regimen, the route of administration, and drug indication.

8.13.1 Permitted Medications

Subjects using oral contraceptives, hormone-replacement therapy, or other maintenance therapies that are not Excluded Therapies (Appendix B) may continue their use during the study.

Stable doses of the following therapies for pruritus and/or atopic dermatitis will be allowed as adjunctive therapies during the study:

- Oral H1 antihistamines
- Bland emollients

A detailed guideline will be shared with the sites and subjects to ensure consistency in study medication treatment application and relation to body washing, emollient use, and other chemical exposure to skin.

Low- or mid-potency topical corticosteroids will be allowed per subjects' usual medication, frequency, and dose throughout the study from screening to washout, treatment, and follow-up periods as rescue medication up to 7 days per month.

Subjects using any of these adjunctive therapies should maintain their regimen without clinically significant changes in type, dose or frequency throughout the screening, treatment, and follow-up periods.

All participants will be screened for concomitant medications prior to inclusion into the study. Any concomitant medication to treat adverse events will be recorded in the Concomitant Medication section of the eCRF.

8.13.2 Excluded Therapies

Therapies and medications that are excluded from the initial Screening visit through the follow-up period are listed in Appendix B.

8.14 Rescue Medications

8.14.1 Rescue Medication Permitted During the Study

Over the course of the study, the Investigator will monitor and evaluate subject's condition and determine whether rescue therapy may be necessary. The use of medications to treat a subject's atopic dermatitis during the study will be permitted if a medical professional determines that it becomes medically necessary. Such rescue treatment should not exceed 7 days per month for the duration of the trial (screening, washout, treatment, and follow-up), e.g., no more than 3 days total during the 2 week washout period, no more than 7 days total during the 4 week treatment period, and no more than 7 days total during the 4 week follow-up period. If a subject requires treatment for more than 7 days per month or requires systemic therapy, they may be discontinued from the study. Medications permitted are listed in Table 8.1.

In the event of an atopic dermatitis or pruritus exacerbation in between visits, subjects should contact study staff as soon as they are able. Subjects should seek approval on the use of rescue medications. In the event that the subject did not notify the study staff, every effort should be made by the subject to contact the PI or study staff as soon as feasible. The details of the medication should be recorded in the eDiary and transferred to the CRF. Details recorded should include medication name, date of administration, dosage, and number of applications.

Table 8.1 Permitted Rescue Medication

**Class 4 – Mid-strength**	
Clocortolone pivalate (0.1%)	Cloderm® Cream
Mometasone furoate (0.1%)	Elocon® Cream
Triamcinolone acetonide (0.1%)	Aristocort® A Cream, Kenalog® Ointment
Betamethasone valerate (0.1%)	Valisone Ointment
Fluocinolone acetonide (0.025%)	Synalar® Ointment
**Class 5 – Lower Mid-strength**	
Fluticasone propionate (0.05%)	Cutivate® Cream/Cutivate Lotion
Prednicarbate (0.1%)	Dermatop® Cream
Hydrocortisone probutate (0.1%)	Pandel® Cream
Triamcinolone acetonide (0.1%)	Aristocort A Cream, Kenalog Lotion
Fluocinolone acetonide (0.025%)	Synalar Cream
**Class 6 – Mild**	
Alclometasone dipropionate (0.05%)	Aclovate® Cream/Ointment
Desonide (0.05%)	Verdeso™ Foam, Desonate Gel™
Triamcinolone acetonide (0.025%)	Aristocort A Cream, Kenalog Lotion
Hydrocortisone butyrate (0.1%)	Locoid Cream/Ointment
Fluocinolone acetonide (0.01%)	Derma-Smoothe/FS® Scalp Oil, Synalar Topical Solution
**Class 7 – Least potent**	
Hydrocortisone (2%/2.5%)	Nutracort® Lotion, Synacort Cream
Hydrocortisone (0.5 to 0.1%)	Cortaid® Cream/Spray/Ointment and many other over-the-counter products

8.14.2 Use of Excluded Therapies During the Treatment Period

Subjects who use an excluded therapy during the treatment period for treatment of atopic dermatitis or pruritus outside of the rescue medication allowance will be discontinued from study drug treatment, with the following exception:

- Subjects who require short treatment for indications other than atopic dermatitis or pruritus (up to a total of 3 days over the treatment period) with sedatives, tranquilizers, opioids, and/or topical therapies will not be required to discontinue from study drug treatment.

Use of any excluded therapies will be recorded for subjects who receive them.

8.15 Handling of Investigational Product

Subjects will receive study IP in 30 ml white bottles. Each bottle will be anticipated for use over 10 days and brought to all study appointments. The IP bottle can be kept at room temperature while in use by the subject.

Subjects will be asked not to subject the treatment kit to conditions which are unnatural or harmful to the product, such as excessive heat (temperatures over 77°F (25°C)) and freezing temperatures (below 32°F (0°C)). Subjects may travel with their study medication but should not leave it in a hot car, outside in the cold temperatures, etc. Subjects will also be asked not to tamper or cause damage to IP.

9 CONTRACEPTION REQUIREMENTS

Effective contraception is required for all women physiologically capable of becoming pregnant during study participation. Women of child-bearing potential must agree to use an acceptable form of contraception for up to 2 weeks after the study completion as detailed. Women of childbearing potential are defined as any female who has experienced menarche and who is NOT permanently sterile (e.g., by tubal ligation) or postmenopausal. Postmenopausal is defined as 12 consecutive months with no menses without an alternative medical cause.

Effective contraception methods include:

- Use of oral, injected or implanted hormonal methods of contraception or placement of an IUD or IUS or other forms of hormonal contraception that have comparable efficacy, for example hormone vaginal ring or transdermal hormone contraception
- Use of barrier methods (i.e., condom, diaphragm) used with a spermicide (i.e., foam, cream, or gel that kills sperm)
- Total abstinence (when this is in line with the preferred and usual lifestyle of the study participant).

Periodic abstinence (i.e., calendar, ovulation, symptothermal, postovulation methods) and withdrawal are not acceptable methods of contraception.

Male participants of the study who are having sexual intercourse with a woman who can become pregnant must use an acceptable form of birth control while participating in the study. Additionally, male participants are expected to inform their female partners of their participation in a research study of an investigational drug, the effects of which on a fetus and on a pregnant woman are unknown. Male participants will also be expected to provide their female partners with the contraception requirements information previously described and the study doctor’s contact information for questions.

Payment for all aspects of obstetrical care, child-or related care will be the study participant's responsibility.

In case of pregnancy, Investigational Product should be discontinued and the Sponsor should be informed immediately. Follow-up of the pregnancy will be mandatory until the outcome is available.

10 STUDY PROCEDURES

10.1 Pre-screening procedures

Study subjects will be recruited from among participating hospitals, clinics, and diagnostic centers or from general population under the responsibility of a participating Investigator. Prior to initiation of the recruitment phase, participating Investigators will identify a pool of potential study subjects. Each of these centers will identify potentially eligible patients in advance, by either reviewing past medical records and diagnoses, screening in clinics, referral from other physicians, or other sources of recruitment, to identify those aged 18 to 65 with clinical diagnosis of mild or moderate Atopic Dermatitis. Medical records from patient's dermatologist or primary care physician to confirm the diagnosis are optional. Verbal confirmation of the diagnosis present for > 12 months is sufficient to fulfill this criterion.

10.2 Informed Consent Procedures

Eligible participants may only be included in the study after providing a consent using the IRB-approved informed consent form. Informed consent must be obtained before conducting any study-specific procedures (i.e., all of the procedures described in the protocol). The process of obtaining informed consent should be documented in the participant's source documents. The date of signing of informed consent (and withdrawal, if later withdrawn) should be documented in the eCRF.

10.3 Study Assessments

Study activities will take place according to the Time and Events table (Appendix A).

Demographics and a complete medical history will be recorded during the Screening period. The medical history will include a complete review of all current diseases and their respective durations and treatments.

10.4 Inclusion procedures

Once all inclusion/exclusion criteria are fulfilled, the patient becomes eligible for randomization and inclusion into the treatment period. Treatment allocation will be performed as stated above

in Section 8.5. Study medication will be delivered as stated in Section 8.6. Patients will be counseled on product application and eDiary completion.

10.5 Timing of patient's visits to the clinic

Patients will be asked to report to the clinic for their scheduled appointments. If a subject is unable to schedule an appointment within the required time frame, study staff will be asked to reschedule the patient to a day when they are able to come in within predetermined time frame.

10.6 Description by type of visit

10.6.1 Screening Visit (-21 to -14) – Visit 1

- informed consent completed and signed
- inclusion and exclusion criteria
- demographic data
- medical/surgical history
- concomitant medications
- physical exam
- vital signs
- blood for clinical chemistry, serology and hematology
- urine pregnancy test (for women of childbearing potential)
- NRS assessment
- POEM questionnaire
- Pruritus 5-D questionnaire
- IGA scoring assessment
- EASI scoring assessment
- study counseling
- start eDiary
- start AE monitoring

10.6.2 Washout Phase (-14 to -1)

For those subjects who meet the eligibility criteria at the Screening visit and is determined by the Principal Investigator to be eligible for the study after the safety labs testing results are available, subjects will be notified by telephone to start the Washout period phase. Subjects will be allowed to use their regular emollients if needed but will be asked to stop using bleach or vinegar baths.

Stable doses of the following therapies for pruritus and/or atopic dermatitis will be allowed as adjunctive therapies during the study:

- Oral H1 antihistamines
- Bland emollients

A detailed guideline will be shared with the sites and subjects to ensure consistency in study medication treatment application and relation to body washing, emollient use, and other chemical exposure to skin.

Low- or mid-potency topical corticosteroids will be allowed per subjects' usual medication, frequency, and dose during the washout period but no more than 7 days per month as rescue medication.

Subjects using any of these adjunctive therapies should maintain their regimen without clinically significant changes in type, dose or frequency throughout the screening, treatment, and follow-up periods.

Subjects will be prohibited from using Excluded Medications/Therapy listed in Appendix B.

- phone call to subjects
- study counseling

10.6.3 Study Day 0-Baseline visit (0) – Visit 2

- inclusion and exclusion criteria
- concomitant medications
- medical history
- vital signs
- urine pregnancy test (for women of childbearing potential)
- NRS assessment
- POEM questionnaire
- Pruritus 5-D questionnaire
- IGA scoring assessment
- EASI scoring assessment
- allocation of a randomized treatment kit number via IWRS
- delivery of the corresponding Investigational Product
- obtain study medication weight for Investigational Product compliance
- first application of Investigational Product (under medical supervision)
- study counseling
- eDiary review
- recording of AEs if any

10.6.4 Day 5- Phone call to patient

The Day 5 visit is a telephone visit that occurs 5 days (±2 days) after the Baseline visit. Study staff will discuss IP application with the subject, answer any questions and counsel the subject on any other matters related to the study.

- phone call to subjects
- application of study drug
- study counseling

10.6.5 Day 14 and Day 28 study visits – Visit 3 and Visit 4

- concomitant medications
- vital signs
- blood for clinical chemistry and hematology (Day 28)
- urine pregnancy test (for women of childbearing potential) (Day 28)
- NRS assessment
- POEM questionnaire
- Pruritus 5-D questionnaire
- IGA scoring assessment
- EASI scoring assessment
- obtain study medication weight for Investigational Product compliance
- dispense investigational product to patient (Day 14)
- collect study medication
- application of study drug
- eDiary
- study counseling
- recording of AEs if any

10.6.6 Day 56-End of Study Visit – Visit 5

- concomitant medications
- vital signs
- physical exam
- urine pregnancy test (for women of childbearing potential)
- NRS assessment
- POEM questionnaire
- Pruritus 5-D questionnaire
- IGA scoring assessment
- EASI scoring assessment
- eDiary
- recording of AEs if any

10.6.7 Unscheduled/Unanticipated Study visit

If an event arises that requires patient to come in to the research center, subjects should be scheduled for the Unscheduled visit and assessments are performed based on investigator discretion.

10.6.8 Early Termination Visit

Every attempt should be made to complete all visits during the defined window periods. Subjects who do not complete all required study visits and withdraw from the study before the Day 56 final visit will be asked to complete the Early Termination Visit.

During the visit, the following will be obtained:

- concomitant medications
- vital signs
- physical exam
- urine pregnancy test (for women of childbearing potential)
- NRS assessment
- POEM questionnaire
- Pruritus 5-D questionnaire
- IGA scoring assessment
- EASI scoring assessment
- blood for clinical chemistry and hematology (if visit occurs before Day 28)
- eDiary
- collect study medication (if visit occurs before Day 28)
- obtain study medication weight for Investigational Product compliance
- recording of AEs if any

11 STUDY ASSESSMENTS

11.1 Safety Assessments

11.1.1 Vital Signs

Vital signs will include measurements of heart rate, sitting blood pressure, respiration rate, and temperature. Vital signs will be assessed as outlined in Appendix A and at unscheduled study visits when clinically indicated. The subjects' height and weight will be measured as outlined in Appendix A.

11.1.2 Physical Examinations

The physical examination will be performed at Screening, Day 56, and at any Unanticipated visit should one occur as outlined in Appendix A. The physical examination includes an assessment of general appearance and a review of systems (dermatologic, head, eyes, ears, nose, mouth/throat/neck, thyroid, lymph nodes, respiratory, cardiovascular, gastrointestinal, extremities, musculoskeletal, and neurologic systems).

11.1.3 Laboratory Assessments

Blood samples should be taken using standard venipuncture techniques. Blood sampling will be performed according to the site SOPs.

The following laboratory variables will be determined as outlined below:

The following routine clinical chemistry, hematology and Lipid Panel will be performed according to Time and Events table (Appendix A): Albumin, Alkaline Phosphatase, ALT, AST, Total Bilirubin, BUN, BUN:Creatinine ratio, Calcium, Chloride, Creatinine, eGFR, Glucose, Potassium, Sodium, Uric Acid, Total Protein, Bicarbonate

Lipid Panel: HDL Cholesterol, LDL cholesterol, Total Cholesterol, Triglycerides, VLDL Cholesterol, LDL/HDL Cholesterol Ratio, Non-HDL Cholesterol.

Urinalysis: Color, clarity, pH, specific gravity, bilirubin, glucose, ketones, leukocytes, nitrite, blood, protein, urobilinogen, microscopic analysis.

Hematology: WBC, RBC, Hemoglobin, Hematocrit, Platelets, WBC Differential (Neutrophils, Lymphocytes, Monocytes, Eosinophils, Basophils)

Serology will only be done at Screening: HIV Ab, HCV Ab, and HBsAg.

Pregnancy testing: All females of childbearing potential will have a local urine pregnancy test performed at specified visits.

Patients will be asked to fast for at least 8 hours before all blood tests are done. An exception will be made on the laboratory testing for the screening visit such that either fasting or non-fasting blood samples can be collected. This is to avoid requesting participants to fast prior to the informed consent.

The total blood volume collected for clinical labs for Screening visit will be approximately 20 ml of whole blood. Volume collected for subsequent visits would be approximately 10 ml of whole blood.

Any value outside the normal range will be flagged for the attention of the Investigator or designee at the site. The Investigator or designee will indicate whether or not the value is of clinical significance. If the result of the clinical chemistry test from the samples taken during the screening phase is indicated as clinically significant, the study subject will NOT be allowed into the study.

11.2 Efficacy Assessments

Efficacy endpoints will be descriptively summarized and will include the number of observations, mean, median, standard deviation, minimum, and maximum of scores/values at all applicable time points and for all treatments in the ITT Population.

11.2.1 EASI Assessment

An EASI score is used to measure the extent (area) and severity of atopic eczema (Eczema Area and Severity Index). The score will be calculated at the times indicated in the Time and Events Table (Appendix A). The investigator will assess improvement of eczema based on intensity and severity of the disease. EASI score sheet can be found in Appendix C.

The severity strata for the EASI are as follows:
0 = clear
0·1-1·0 = almost clear
1·1-7·0 = mild
7·1-21·0 = moderate
21·1-50·0 = severe
50·1-72·0 = very severe

11.2.2 Patient Oriented Eczema Measure

The POEM is a tool developed by the University of Nottingham, United Kingdom, for monitoring atopic dermatitis severity (Appendix D). The subject will complete the questionnaire at each of the assessment timepoints as outlined in Appendix A.

Each of the 7 questions in the POEM questionnaire carries equal weight and is scored from 0 to 4:

- No days = 0.
- 1 to 2 days = 1.
- 3 to 4 days = 2.
- 5 to 6 days = 3.
- Every day = 4.

Scores are then added to yield a total score of 0 to 28; higher scores mean the greater the severity of atopic dermatitis.

11.2.3 Itch Numeric Rating Scale

The Itch NRS is a validated, self-reported instrument for measurement of itch intensity. It uses a 24-hour recall period and asks subjects to rate the intensity of their itch on an 11-point scale ranging from 0 (no itch) to 10 (worst itch imaginable). Higher scores indicate greater itch intensity. In this study, both worst itch intensity (WI-NRS) and average itch intensity (AI-NRS) during a 24-hour recall period will be captured. Subjects will record their Itch NRS scores once

daily via eDiary throughout the screening, treatment, and follow-up periods, as outlined in Appendix A.

The question for WI-NRS would be, “Please rate the itching severity due to your atopic dermatitis by circling the number that best describes your worst level of itching in the past 24 hours.”

The question for AI-NRS would be, “Please rate the itching severity due to your atopic dermatitis by circling the number that best describes your average level of itching in the past 24 hours.”

0 1 2 3 4 5 6 7 8 9 10

0 = No itching 10 = Worst itch imaginable

11.2.4 Investigator Global Assessment Score (IGA)

IGA will be performed at the times indicated (Screening, Baseline, Day 14, Day 28, and Day 56) in the Time and Events Table of the Protocol (Appendix A) as static IGA. The Investigator will assess improvement of Atopic Dermatitis based on the 5-point severity scale summarized below and in Appendix E.

Score	Category	Definition
0	Clear	Minor residual discoloration, no erythema or induration/papulation, no oozing/crusting
1	Almost Clear	Trace faint pink erythema with almost no induration/papulation and no oozing/crusting
2	Mild disease	Faint pink erythema with mild induration/papulation and no oozing/crusting
3	Moderate disease	Pink-red erythema with moderate induration/papulation and there may be some oozing/crusting
4	Severe disease	Deep/bright red erythema with severe induration/papulation with oozing/crusting

11.2.5 5-D Pruritus Scale

The 5-D Pruritus Scale is a validated, multi-dimensional measure of itching that assesses the five domains of degree, duration, direction, disability and distribution (Appendix F). Subjects rate their symptoms over the preceding 2-week period on a 1 to 5 scale, with 5 being the most affected. Subjects will complete the 5-D Pruritus Scale during study visits as outlined in Appendix A.

11.3 eDiary and Efficacy Assessments

All patient reported outcomes and clinical outcome assessments (ePRO/eCOA) will be obtained electronically using either a smartphone application or a tablet. Participants will be provided with eDiary at screening to initiate daily diary entries from screening to follow-up and will include responses to WI-NRS, AI-NRS, daily dose confirmation (during the treatment period),

rescue medication (if any), and local skin tolerability (Day 1 to Day 7 of treatment). In addition, participants' usual adjunctive therapies (e.g., bland emollient, oral H-1 antihistamine) may be collected using eDiary or CRF at screening and optionally throughout the study. POEM and 5-D Pruritus Scale will be reported by the participants at the designated site visits using a site provided tablet. IGA and EASI will also be assessed at the designated site visits by the clinician.

11.4 Patient Reported Local Tolerability

Solicited local adverse reactions (e.g., itching, new rash, pain, tenderness, stinging, skin color change, etc.) and severity from subjects will be collected during the first week of treatment using eDiary with the questions outlined in Appendix G to inform local skin tolerability.

11.5 Pregnancy Reporting

Any pregnancy will be reported by study participants during their study participation. Participants who report pregnancy or lactation during the review of inclusion/exclusion criteria prior to randomization will not be enrolled in the trial. In case of pregnancy, Investigational Product should be discontinued and the Sponsor informed. Follow-up of the pregnancy will be mandatory until the outcome is available.

11.6 Study Completion

A completed participant is one who has completed all study visits. Day 56 study visit is defined as the participant's last visit.

11.7 Subject Withdrawal Criteria

A subject may withdraw from the study at any time at his/her own request, or may be withdrawn at any time at the discretion of the Investigator for safety, behavioral, or administrative reasons.

Reasons for withdrawal (subjects who refuse to return for any remaining study visits) or discontinuation (subjects who prematurely stop the application) at any time during the study may include, but are not limited, to the following:

- For safety reasons, either at the discretion of the Investigator or at the subject's request
- For protocol violations at the discretion of AOBiome
- Due to concomitant therapy that could interfere with the results of the study (the Investigator will report all such information on the CRFs and decide, in accordance with AOBiome, whether the subject is to be withdrawn).

All premature discontinuations and their causes must be carefully documented by the Investigator on the CRF or if needed on the AE form.

If, for any reason, a subject is withdrawn before completing the final visit, the reason for termination will be entered on the CRF. All data gathered on the subject prior to termination will

be made available to AOBiome. Subjects not completing the entire study should be fully evaluated when possible. The appropriate CRFs should be completed.

If the subject chooses to withdraw before completing the study, the subject should notify the study coordinator who will instruct the subject on completion of assessments for End of Study (EOS) visit. For subjects who refuse to complete the assessments for their early termination, every attempt must be made to check on their status, using any mode of communication such as telephone, email, fax, or text.

12 STATISTICAL CONSIDERATIONS

12.1 Sample Size

Approximately 576 subjects may be enrolled to account for 16.7% drop out rate prior to completing the study. A total of 160 evaluable subjects per group (480 total) are required to achieve at least 80% power to detect a pairwise difference of 0.65 in mean WI-NRS change from baseline to Week 4 between one of two active doses of B244 and vehicle control when assuming a standard deviation of 2.5 and applying a Dunnett Testing Method at a one-sided familywise error rate of 0.10.

12.2 Populations for Analysis

Intent to Treat (ITT): All randomized participants who apply at least 1 dose of study medication. Subjects will be grouped as randomized.

Per Protocol: All subjects in the ITT population without any major protocol deviations that may have an impact on the efficacy assessments, who complete their Week 4 visit, and who administer at least 50% of investigational product (IP). Subjects will be grouped as treated.

Safety: All subjects who apply at least 1 dose of study medication. Subjects will be grouped as treated.

12.3 Data Analysis

A separate statistical analysis plan will be developed. All data collected will be documented using summary tables, figures, and/or patient data listings. For continuous variables, descriptive statistics (number (n), mean, median, standard deviation, minimum and maximum) will be presented. For categorical variables, frequencies and percentages will be presented. As appropriate, a 95% CI will be presented. Graphical displays will be presented, as appropriate. Data will be presented by treatment group and overall.

Descriptive statistics for each treatment group will be provided for clinical laboratory values (e.g., hematology, serum chemistry, and urinalysis) and vital signs data, presented as both actual values and changes from baseline relative to each on-study evaluation. Abnormal clinical laboratory values will be listed.

12.3.1 Disposition

A tabulation of the disposition of subjects will be presented, including the number enrolled, the number randomized, the number treated, and the reasons for study discontinuation. Summaries of the number in each analysis set will be presented. Entry criteria violations and protocol deviations will be listed.

12.3.2 Demographic and Baseline

Demographic and baseline characteristic data summarization will be performed in order to descriptively assess the comparability of treatment groups. Data to be tabulated will include age, race, ethnicity, height, weight, and BMI, as well as baseline characteristics related to medical history.

12.4 Safety Analyses

12.4.1 Definitions

All adverse events recorded during the study will be coded according to MedDRA.

12.4.2 Adverse Events

All adverse events (AEs) recorded during the study through the date of randomization through 28 days after the last dose of study drug will be analyzed.

Adverse events will be summarized by treatment group using the Medical Dictionary for Regulatory Activities (MedDRA) system organ class and preferred term. Separate tabulations will be produced for all treatment-emergent AEs (TEAEs), treatment-related AEs (those considered by the Investigator as at least possibly drug related), SAEs, discontinuations due to AEs, and AEs ≥ Grade 3 severity. By-subject listings will be provided for any deaths, SAEs, and AEs leading to discontinuation.

AE's will be summarized using incidence rates. Therefore, each subject will only contribute once for a given adverse event SOC or PT.

Any treatment emergent AEs related to local safety (e.g., erythema, edema, induration, vesiculation, etc.) will be collected by the investigator during scheduled clinic visits. In addition, solicited local adverse reactions (e.g., itching, new rash, pain, tenderness, stinging, skin color change, etc.) and severity from subjects will be collected during the first week of treatment using eDiary to inform local skin tolerability.

A summary of the incidence of any adverse event, SAE, and adverse events leading to discontinuation will be presented. Summaries will display, by treatment group, the incidence of patients with events, the frequency of patients with events within each primary system organ class and by preferred terms. For each preferred term and each system organ class a patient will

be counted only once. For summaries on severe or drug-related AE, for a given patient, the highest severity or relationship for a specific preferred term will be considered.

12.4.3 Deaths and Serious Adverse Events

Serious adverse events and events leading to death will be summarized overall and by primary system organ class and preferred term.

12.4.4 Adverse Events leading to treatment discontinuation

Adverse events leading to treatment discontinuation will be summarized overall and by primary system organ class and preferred term.

12.5 Efficacy Analyses

All efficacy analyses will be performed on the ITT population and PP population. Regression models will include all eligible subjects for the respective analysis, from the 3 treatment arms.

Hypothesis tests for the primary efficacy endpoint will be performed using a Dunnett Testing Method, applying pairwise comparisons of each respective B244 dose group to vehicle using a one-sided familywise error rate of 0.10. No additional adjustments will be made for multiple testing. As such, p-values from analysis of secondary and exploratory efficacy analyses must be interpreted in an exploratory fashion.

The primary endpoint of mean change from baseline to Week 4 in WI-NRS will be analyzed using analysis of covariance (ANCOVA) models.

In addition, continuous efficacy endpoints will be summarized using descriptive statistics at Baseline, Week 2, Week 4 and Week 8 for actual values and change-from-baseline values. The difference in treatment groups in change-from-baseline values at post-baseline visits will be analyzed using a mixed model with repeated measures to account for within subject variability and including visit (Baseline vs. Week 2, Week 4, Week 8), treatment group, visit-by-treatment interaction, and baseline value as explanatory variables.

Categorical variables will be summarized using descriptive statistics and analyzed using a logistic regression model at each respective timepoints. Generalized estimating equations to account for repeated measures and within-subject variability may also be applied.

Supportive analyses may be performed combining the B244 dose groups in comparison against vehicle and comparison of the B244 groups to one another.

12.6 Handling of dropouts or missing data

All available data will be analyzed. The details for any imputations for missing data will be documented in the trial's Statistical Analysis Plan. Subjects who dropout after enrollment but prior to randomization will be replaced.

12.7 Clinical Trial Protocol deviations

At minimum, the following deviations will be summarized on the ITT patient population:

- Inclusion or exclusion criteria not satisfied.
- Deviations related to the Investigational Product administration
- Not permitted concomitant medications.

13 ADVERSE EVENTS (AE) AND SERIOUS ADVERSE EVENTS (SAE)

The Investigator and study staff are responsible for detecting and recording AEs and SAEs during scheduled safety evaluations and whenever such information is brought to their attention. This section of the protocol provides definitions and detailed procedures to be followed.

13.1 Definition of an AE

An AE is any untoward medical occurrence in a study participant which is temporally associated with the use of a medicinal product, regardless of its potential relationship to the medicinal product. An AE, therefore, can be any unfavorable or unintended sign, including an abnormal symptom, or disease (new or exacerbated), whether or not related to the investigational product (IP).

Examples of an AE include:

- Exacerbation of a pre-existing condition including either an increase in frequency and/or intensity of the condition.
- New conditions detected or diagnosed after investigational product administration even though it may have been present prior to the start of the study.
- Signs, symptoms of a drug interaction.
- Signs, symptoms of a suspected overdose of either investigational product or a concurrent medication (overdose per se should not be reported as an AE/SAE).

AEs may include pre- or post-treatment events that occur as a result of protocol-mandated procedures (i.e., modification of participant's previous therapeutic regimen).

13.2 Definition of a SAE

A serious adverse event is any untoward medical occurrence that, at any dose:

a) results in death.
b) is life-threatening.

NOTE: The term 'life-threatening' in the definition of 'serious' refers to an event in which the participant was at risk of death at the time of the event. It does not refer to an event, which hypothetically might have caused death, if it were more severe.

c) requires hospitalization or prolongation of existing hospitalization.

NOTE: In general, hospitalization signifies that the participant has been detained (usually involving at least an overnight stay) at the hospital or emergency ward for observation and/or treatment that would not have been appropriate in the physician's office or out -patient setting. Complications that occur during hospitalization are AEs. If a complication prolongs hospitalization or fulfills any other serious criteria, the event is serious. When in doubt as to whether "hospitalization" occurred or was necessary, the AE should be considered serious. Routine hospitalizations or elective surgeries are generally not regarded as SAEs.

d) results in disability/incapacity

NOTE: The term disability means a substantial disruption of a person's ability to conduct normal life functions. This definition is not intended to include experiences of relatively minor medical significance such as uncomplicated headache, nausea, vomiting, diarrhea, influenza, and accidental trauma (e.g. sprained ankle) which may interfere or prevent everyday life functions but do not constitute a substantial disruption.

e) is a congenital anomaly/birth defect
f) Medical or scientific judgment should be exercised in deciding whether reporting is appropriate in other situations, such as important medical events that may not be immediately life-threatening or result in death or hospitalization but may jeopardize the participant or may require medical or surgical intervention to prevent one of the other outcomes listed in the above definition. These should also be considered serious. Examples of such events are invasive or malignant cancers, intensive treatment in an emergency room or at home for allergic bronchospasm, blood dyscrasias or convulsions that do not result in hospitalization, or development of drug dependency or drug abuse.

13.3 Time-period, Frequency, and Method of Detecting AEs and SAEs

All AEs occurring after administration of the first dose of study medication and on or before the final assessment must be reported as AEs. All AEs must be recorded irrespective of whether they are considered drug-related.

At each assessment in the period defined above, AEs will be evaluated by the Investigator and recorded.

Any AEs already documented at a previous assessment and designated as ongoing, should be reviewed at subsequent assessments as necessary. If these have resolved, this should be documented. Changes in intensity or frequency of AEs should be recorded as separate events (i.e., a new record started).

The recording of AEs and SAEs are described in Section 13.4 ("Recording of AEs and SAEs").

13.4 Recording of AEs and SAEs

All clinical events, including either observed or volunteered problems, complaints or symptoms are to be recorded on the Adverse Events page(s) of the CRF. The need to capture this information is not dependent upon whether the clinical event is associated with study treatment. Adverse clinical events resulting from concurrent illnesses or reactions to concurrent medications are also to be recorded. In order to avoid vague, ambiguous, or colloquial expressions, the AE should be recorded in standard medical terminology rather than the participant's own words.

Each adverse clinical event is to be evaluated for duration, intensity, and whether the event may be associated with the investigational product (IP) or other causes. Start and stop dates, relationship to investigational product (IP), medical management, and alternative causality of event must be recorded in the Adverse Events section of the CRF. AEs believed to be possibly related to investigational product (IP) must be followed until resolution.

13.5 Evaluating AEs and SAEs

13.5.1 Severity Rating

The severity of an adverse event (AE and SAE) is to be scored according to the following scale:

**Mild**	Awareness of symptoms, but easily tolerated
**Moderate**	Discomfort enough to cause interference with usual activity
**Severe**	Incapacitating with inability to work or perform usual activity

An AE that is assessed as severe should not be confused with a SAE. An event is defined as 'serious' when it meets one of the pre-defined outcomes as described in Section 13.2 "Definition of a SAE".

13.5.2 Relationship to Investigational product (IP)

SAEs will be classified as "**not related**" or "**related**" (including unknown).

For AEs, the relationship to study treatment is to be assessed according to the following definitions:

Definitely not related: The AE is definitely not related to the drug. This designation should be reserved for those events which occur prior to study treatment or for those events which cannot be even remotely related to study participation (e.g., injuries sustained in an automobile accident).

Unlikely related: There is no reasonable association between the study treatment and the suspected event and the event could have been produced by the participant 's clinical state or other modes of therapy administered to the participant.

Possibly related: The suspected adverse event may or may not follow a reasonable temporal sequence from study treatment administration but seems to be the type of reaction that cannot be dismissed as unlikely. The event could have been produced or mimicked by the participant 's clinical state or by other modes of therapy concomitantly administered to the participant.

Probably related: The suspected adverse event follows a reasonable temporal sequence from study treatment administration, abates upon discontinuation of the treatment, and cannot be reasonably explained by the known characteristics of the participant 's clinical state.

Definitely related: This designation should be reserved for those events which have no uncertainty in their relationship to treatment administration.

13.6 Pregnancy

Any pregnancy that occurs in a female participating in the study must be reported to the Safety Team or to a designated Safety email or fax number provided by the Safety Team within 24 hours of learning of the pregnancy. Follow-up must occur to determine the outcome of the pregnancy (including premature termination) and the status of mother and child. Pregnancy complications and elective terminations for medical reasons must be reported as AEs or SAEs. Spontaneous abortions must be reported as an SAE.

Any SAE occurring in association with a pregnancy and considered by the Investigator as related to the investigational product must be promptly reported to the Sponsor, even if the event occurred after the participant completed the study.

The Investigator must attempt to collect pregnancy information on any female partners of male participants who become pregnant while the male participant is enrolled in the study. Pregnancy information must be reported to the Sponsor as described above.

13.7 Prompt Reporting of SAEs to the Sponsor

In the case of a Serious Adverse Event the Investigator must immediately:

- **SEND** (within 1 working day) the signed and dated SAE Report Form to the Safety Team or to a designated Safety email or fax number provided by the Safety Team;
- **ATTACH** a photocopy of all examinations carried out and the dates on which these examinations were performed. Care should be taken to ensure that the patient's identity is protected and the patient's identifiers in the Clinical Trial are properly documented on all copies of source documents provided to the Sponsor. For laboratory results, include the laboratory normal ranges;
- **Follow-up of any Serious Adverse Event** that is fatal or life threatening should be provided within one additional calendar week. The treatment code will be unblinded for reporting of Serious Adverse Events that are unexpected and determined to be related to the use of the Investigational Product.

Follow-up

- The Investigator should take all appropriate measures to ensure the safety of the patients, including referral to a specialist if indicated. Notably the Investigator should follow up the outcome of any adverse events (clinical signs, laboratory values or other, etc.) until the return to normal or stabilization of the patient's condition;
- In the case of any Serious Adverse Event, the patient must be followed up until clinical recovery is complete and laboratory results have returned to normal, or until progression has been stabilized. This implies that follow-up may continue after the patient has left the Clinical Trial and that additional investigations may be requested by the Safety Team;
- In case of any Serious Adverse Event brought to the attention of the Investigator at any time after cessation of Investigational Product and considered by the Investigator to be related to the Investigational Product, this should be reported to the Safety Team.

**AOBiome Reportable Events Hotline**<br>**Email:** [redacted]

14 ETHICAL AND REGULATORY STANDARDS

14.1 Ethical Conduct of Study

This clinical trial was designed and shall be implemented, executed, and reported in accordance with the ICH Harmonized Tripartite Guidelines for Good Clinical Practice, with applicable local

regulations (including US Code of Federal Regulations Title 21), and with the ethical principles laid down in the Declaration of Helsinki.

The Investigator(s) should conduct the study in accordance with this protocol, the Declaration of Helsinki and ICH GCP guidelines and FDA regulations. The Investigator(s) and the Sponsor will sign the protocol and study contract, to confirm agreement. The Investigator(s) will not implement any amendment (deviation or changes of the protocol) without agreement by the Sponsor and IRB approval, except where necessary to eliminate immediate hazard(s) to study participants, or when change(s) involve only logistical or administrative aspects of the study. Records that may reveal the identities of participants must be well protected, with consideration given to confidentiality and the right to privacy of participants.

14.2 Laws and Regulations

This Clinical Trial will be conducted in compliance with all international laws and regulations, and national laws and regulations of the countries in which the clinical trial is performed, as well as any applicable guidelines. The trial will be registered and updated on www.clintrials.gov and on other sites, as deemed appropriate.

14.3 Informed Consent

Each participant must be provided with a statement that the investigation involves research and that the IRB has approved solicitation of participants to participate; a fair explanation of the procedures to be followed and their purposes, including identification of any procedures which are experimental; a description in lay language of any possible side effects; a description of any attendant discomforts and risks reasonably to be expected; a description of any benefits reasonably to be expected; a disclosure of any appropriate alternative procedures that might be advantageous for the participant; an offer to answer any inquiries concerning the procedures, and instruction that the person is free to withdraw consent and discontinue participation in the project or activity at any time without prejudice to the participant. Payment to research participants for taking part in the study is based on time and inconvenience. All information concerning payment, including the schedule of payments, must be set forth in the informed consent, including a disclosure that the Investigator is being paid to perform the stated research.

A participant must give consent to take part in the study. Participants below the age of majority in the municipality must give written assent to participate in this study. This consent must be dated and retained by the Principal Investigator as part of the study records. A downloadable digital copy shall be given to the person signing the form. The informed consent process must be documented in the participant’s source documents.

The Investigator agrees that the Sponsor, its employees or agents will have the right from time to time during the course of this study to audit and review pertinent medical records relating to this clinical trial. A statement will be obtained from each person participating in the study permitting the release of his/her medical records as necessary for inspection by authorized personnel of the Sponsor, FDA, and the staff managing the clinical study.

The release of medical records and the review of the contents will be in compliance with the Health Insurance Portability and Accountability Act of 1996 (HIPAA).

14.4 Institutional Review Board/Independent Ethics Committee (IRB/EC)

The protocol and informed consent form and the electronic version of the consent for this study must be approved by the IRB. A copy of the Letter of Approval from the Board, which contains specific identification of the documents approved, must be received by the Sponsor prior to shipment of drug supplies to the Principal Investigator.

All changes to the protocol, as well as a change of Investigator, must also be approved by the IRB and documentation of this approval provided to the study monitor. Records of the IRB's review and approval of all documents pertaining to the study must be kept on file by the Principal Investigator and are subject to FDA inspection at any time. IRB renewal for approval is required each year. The Investigator is to inform AOBiome, in writing, of the approval to continue the study.

14.5 Clinical Monitoring/Record Keeping

There shall be no alterations in the protocol design without the written consent and approval of the Sponsor and the approval of the IRB, except in the case that participants are at immediate risk without immediate implementation of such alterations. In the aforementioned situation, the site should notify the Sponsor and IRB of the deviation as soon as possible, and should seek the written consent and approval of the Sponsor and the approval of the IRB.

All results of this trial must be recorded on eCRFs. Each participant who has been randomized must have a completed eCRF. Reasons for termination must be stated in the early termination section. Study participants are not to be identified by name on eCRFs, but rather by coded identifiers and participant initials.

The study monitor will verify the accuracy of the data by reviewing pertinent source documents such as office records or hospital charts of the participants.

Study records include eCRFs, signed FDA Form 1572, original reports of test results, and signed electronic informed consent forms. IRB approval letters and other documents pertaining to the conduct of the study are to be kept on file by the Investigator. If the study files are assigned to someone else or removed to another location, the Investigator is to notify the study monitor or Sponsor in writing of the change. All study records are subject to FDA inspection at any time. All information supplied to the Investigator by the Sponsor before, during, and after the study is confidential. Such information is to be used solely in connection with the clinical study. The study protocol, IB, and any other pertinent study related materials or records provided are to be maintained in a confidential manner, reviewed carefully with attention to admonitions and

returned to the Sponsor upon request. No part of these materials may be reproduced or transmitted in any form without prior written permission from the Sponsor.

15 ADMINISTRATIVE RULES

15.1 Curriculum Vitae

An updated, signed, and dated copy of the curriculum vitae with the experience, qualifications and training for each Investigator and/or Sub-Investigator(s) will be provided to the Sponsor prior to the beginning of the Clinical Trial.

15.2 Archiving of Study Documentation

The Investigator must maintain confidentiality for all study documentation, and take measures to prevent accidental or premature destruction of these documents.

It is recommended that the Investigator retain the study documents at least fifteen (15) years after the completion or discontinuation of the Clinical Trial. However, applicable regulatory requirements should be taken into account in the event that a longer period is required.

The Investigator must notify the Sponsor prior to destroying any study essential documents within the fifteen (15) year period following the Clinical Trial completion or discontinuation. If the Investigator's personal situation is such that archiving can no longer be ensured by him/her, the Investigator shall inform the Sponsor and the relevant records shall be transferred to a mutually agreed upon designee.

15.3 Internal Safety Review Committee

An internal safety review committee may be set up as needed to protect the ethical and safety interests of participants and to protect the scientific validity of the study. Ad-hoc safety interim analyses might be performed by an independent statistician if the safety review committee identifies potential safety signals during its routine blinded safety review. The details for the analysis plan may be documented in the trial's Statistical Analysis Plan.

16 STUDY MONITORING

16.1 Responsibilities of the Investigator(s)

The Investigator(s) undertake(s) to perform the Clinical Trial in accordance with this Clinical Trial Protocol, ICH guidelines for Good Clinical Practice and the applicable regulatory requirements.

The Investigator is required to ensure compliance with all procedures required by the Clinical Trial Protocol and by study procedures provided by the Sponsor (including security rules). The Investigator agrees to provide reliable data and all information requested by the Clinical Trial Protocol (with the help of the Case Report Form [CRF], Discrepancy Resolution Form [DRF] or

other appropriate instrument) in an accurate and legible manner according to the instructions provided and to ensure direct access to source documents to Sponsor representatives. If any particular circuits have to be defined (e.g., e-CRF, Fax), particular attention should be paid to the confidentiality of the patient's data to be transferred. The Investigator may appoint such other individuals as he/she may deem appropriate as Sub-Investigators to assist in the conduct of the Clinical Trial in accordance with the Clinical Trial Protocol. All Sub-Investigators shall be timely appointed and listed. The Sub-Investigators will be supervised by and under the responsibility of the Investigator. The Investigator will provide them with a Clinical Trial Protocol and all necessary information.

16.2 Responsibilities of the Sponsor

The Sponsor of this Clinical Trial is responsible to Health Authorities for taking all reasonable steps to ensure the proper conduct of the Clinical Trial Protocol with regard to ethics, Clinical Trial Protocol compliance, integrity and validity of the data recorded on the Case Report Forms. Thus, the main duty of the Monitoring Team is to help the Investigator and the Sponsor maintain a high level of ethical, scientific, technical and regulatory quality in all aspects of the Clinical Trial. At regular intervals during the Clinical Trial, the site will be contacted, through monitoring visits, letters or telephone calls, by a representative of the Monitoring Team to review study progress, Investigator and patient compliance with Clinical Trial Protocol requirements, and any emergent problems. During these monitoring visits, the following but not exhaustive list of points will be scrutinized with the Investigator: patient informed consent, patient recruitment and follow-up, Serious Adverse Event documentation and reporting, outcome events documentation and reporting, Investigational Product allocation, patient compliance with the Investigational Product regimen, Investigational Product accountability, concomitant therapy use and quality of data.

16.3 Source document requirements

According to the ICH guidelines for Good Clinical Practice, the Monitoring Team must check the Case Report Form entries against the source documents. The Informed Consent Form will include a statement by which the patient allows the Sponsor’s duly authorized personnel, the Ethics Committee (IRB/EC), and the regulatory authorities to have direct access to source data which supports the data on the Case Report Forms (e.g., patient's medical file, appointment books, original laboratory records, etc.). These personnel, bound by professional secrecy, must keep confidential all personal identity or personal medical information (according to confidentiality rules).

16.4 Use and completion of Case Report Forms (CRFs) and additional requests

The sponsor or CRO will be responsible for activities associated with the data management of this study. This will include setting up a relevant database and data transfer mechanisms, along with appropriate validation of data and resolution of queries. Data generated within this

clinical study will be handled according to the relevant SOPs of the data management and biostatistics departments of sponsor or CRO.

For Electronic Data Capture (EDC):
Study sites will enter data directly into an EDC system by completing the eCRF via a secure internet connection. Data entered into the eCRF must be verifiable against source documents at the study site. Any changes to the data entered into the EDC system will be recorded in an automated, secure audit trail and is Food and Drug Administration (FDA) Code of Federal Regulations (CFR) Title 21 Part 11 compliant.

Data entered into the eCRF will be validated as defined in the Data Validation Specifications (DVS). Validation includes, but is not limited to, validity checks (for example, missing data, range checks) and consistency checks (logical checks between variables) to ensure that study data are accurately reported. Additionally, CRO Data Management will perform aggregate data review as defined in the DVS to ensure that the data are complete, consistent and reasonable. The electronic edit checks will run continually throughout the course of the study and queries reviewed by CRO personnel to assure validity as compared to source records. Manual queries may also be entered into EDC by Monitoring or Data Management personnel to address identified discrepancies.

Medical conditions/procedures will be coded using MedDRA and prior and concomitant medications will be coded using WHODrug.

At the conclusion of the study, each site will be provided with their subject CRFs in Portable Document Format (PDF) for archival. The CRF PDFs will contain subject data, audit trail information, queries including responses, and comments.

17 PUBLICATIONS

All data generated from this study are the property of AOBiome LLC and shall be held in strict confidence along with all information furnished by AOBiome. Independent analyses and/or publication of these data by the Investigator or any member of his/her staff is not permitted without prior written consent of AOBiome.

Any formal presentation or publication of data from this trial will be considered as a joint publication by the Investigator(s) and appropriate Sponsor personnel. Authorship will be determined by mutual agreement. Written permission to the Investigator will be contingent on the review by the Sponsor of the methodology and statistical analysis and any publication or presentation will provide for nondisclosure of AOBiome confidential or proprietary information. In all cases, the parties agree to submit all manuscripts or abstracts to all other parties at least 30 days prior to submission. This will enable all parties to protect proprietary information and to provide comments based on information that may not yet be available to other parties.

18 PROTOCOL ADHERENCE

This protocol defines the study objectives, the study procedures and the data to be collected on study participants. Additional assessments required to ensure the safety of participants should be administered as deemed necessary on a case by case basis. Under no circumstances is an Investigator allowed to collect additional data or conduct any additional procedures for any research related purpose involving any investigational drugs under the protocol.
Investigators ascertain they will apply due diligence to avoid protocol deviations. If an Investigator feels a protocol deviation would improve the conduct of the study this must be considered a protocol amendment, and unless such an amendment is agreed upon by the sponsor and approved by the IRB/IEC and health authorities, where required, it cannot be implemented.

19 CLINICAL TRIAL PROTOCOL AMENDMENTS

Any protocol amendments will be added as stand-alone documents. In addition, any and all revisions dictated by the amendments will be made in the protocol. Each time a protocol is amended, a new amended version date will be added to the cover page.

The Investigator should not implement any deviation from, or changes of the Clinical Trial Protocol without agreement by the sponsor and prior review and documented approval/favorable opinion from the IRB/EC of an amendment. The Investigator is expected to take any immediate action required for the safety of any participant included in this study, even if this action represents a deviation from the protocol. In such cases, the reporting requirements identified in Section 13 must be followed and the Study Lead.

20 CONFIDENTIALITY

All information disclosed or provided by the Sponsor (or any company/institution acting on their behalf), or produced during the Clinical Trial, including, but not limited to, the Clinical Trial Protocol, the CRFs, the Investigator's Brochure and the results obtained during the course of the Clinical Trial, is confidential. The Investigator or any person under his/her authority agrees to undertake to keep confidential and not to disclose the information to any third party without the prior written approval of the Sponsor. However, the submission of this Clinical Trial Protocol and other necessary documentation to the Ethics Committee (IRB/EC) is expressly permitted, the IRB/EC members having the same obligation of confidentiality. The Sub-Investigators shall be bound by the same obligation as the Investigator. The Investigator shall inform the Sub-Investigators of the confidential nature of the Clinical Trial. The Investigator and the Sub-Investigators shall use the information solely for the purposes of the Clinical Trial, to the exclusion of any use for their own or for a third party's account.

21 PROPERTY RIGHTS

All information, documents and Investigational Product provided by the Sponsor or its designee are and remain the sole property of the Sponsor. The Investigator shall not mention any information or the Product in any application for a patent or for any other intellectual property rights. All the results, data, documents and inventions, which arise directly or indirectly from the

Clinical Trial in any form, shall be the immediate and exclusive property of the Sponsor. The complete verified database will be shared with the Operations Committee, which shall have full access to all data. The Sponsor may use or exploit all the results at its own discretion, without any limitation to its property right (territory, field, continuance). The Sponsor shall be under no obligation to patent, develop, market or otherwise use the results of the Clinical Trial. As the case may be, the Investigator and/or the Sub-Investigators shall provide all assistance required by the Sponsor, at the Sponsor's expense, for obtaining and defending any patent, including signature of legal documents.

22 DATA PROTECTION

The patient's personal data and Investigator's personal data which may be included in the Sponsor database shall be treated in compliance with all applicable laws and regulations;

When archiving or processing personal data pertaining to the Investigator and/or to the patients, the Sponsor shall take all appropriate measures to safeguard and prevent access to this data by any unauthorized third party.

23 SPONSOR AUDITS AND INSPECTIONS BY REGULATORY AGENCIES

For the purpose of ensuring compliance with the Clinical Trial Protocol, Good Clinical Practice and applicable regulatory requirements, the Investigator should permit auditing by or on behalf of the Sponsor and inspection by applicable regulatory authorities.

The Investigator agrees to allow the auditors/inspectors to have direct access to his/her study records for review, being understood that these personnel are bound by professional secrecy, and as such will not disclose any personal identity or personal medical information.

The Investigator will make every effort to help with the performance of the audits and inspections, giving access to all necessary facilities, data, and documents. As soon as the Investigator is notified of a future inspection by the authorities, he will inform the Sponsor and authorize the Sponsor to participate in this inspection.

The confidentiality of the data verified and the protection of the patients should be respected during these inspections.

Any result and information arising from the inspections by the regulatory authorities will be immediately communicated by the Investigator to the Sponsor.

The Investigator shall take appropriate measures required by the Sponsor to take corrective actions for all problems found during the audit or inspections.

24 PREMATURE DISCONTINUATION OF THE STUDY OR PREMATURE CLOSE-OUT OF A SITE

24.1 Decided by the Sponsor in the following cases:

1. In the event the results of the Clinical Trial do not appear to be scientifically convincing to the Sponsor;
2. If the aim of the Clinical Trial has become outdated or is no longer of interest;
3. If the information on the product leads to doubt as to the benefit/risk ratio;
4. If the Investigator has received from the Sponsor all Investigational Product, means and information necessary to perform the Clinical Trial and has not included any patient after a reasonable period of time mutually agreed upon;
5. In the event of breach by the Investigator of a fundamental obligation under this agreement, including but not limited to breach of the Clinical Trial Protocol, breach of the applicable laws and regulations or breach of the ICH guidelines for GCP;
6. If the total number of patients are included earlier than expected; In any case the Sponsor will notify the Investigator of its decision by written notice.

24.2 Decided by the Investigator

The Investigator must notify (30 days' prior notice) the Sponsor of his/her decision and give the reason in writing. In all cases (decided by the sponsor or by the Investigator), the appropriate Ethics Committee(s) (IRB/EC) and Health Authorities should be informed.

25 CLINICAL TRIAL PROTOCOL AMENDMENTS

All appendices attached hereto and referred to herein are made part of this Clinical Trial Protocol. The Investigator should not implement any deviation from, or changes of the Clinical Trial Protocol without agreement by the sponsor and prior review and documented approval/favorable opinion from the IRB/EC of an amendment, except where necessary to eliminate an immediate hazard(s) to clinical trial patients, or when the change(s) involves only logistical or administrative aspects of the trial. Any change agreed upon will be recorded in writing, the written amendment will be signed by the Investigator and by the Sponsor and the signed amendment will be filed with this Clinical Trial Protocol. Any amendment to the Clinical Trial Protocol requires written approval/favorable opinion by the IRB/EC prior to its implementation, unless there are overriding safety reasons. In some instances, an amendment may require a change to the Informed Consent Form. The Investigator must receive an IRB/EC approval/favorable opinion concerning the revised Informed Consent Form prior to implementation of the change.

26 REFERENCES

1. Eichenfield LF, Tom WL, Chamlin SL, et al. Guidelines of care for the management of atopic dermatitis: Section 1: Diagnosis and assessment of atopic dermatitis. *J Am Acad Dermatol*. 2014; 70(2):338-351.
2. Arp DJ, Sayavedra-Soto LA, Hommes NG. Molecular biology and biochemistry of ammonia oxidation by Nitrosomonas europaea. *Arch Microbiol* 2002; 178: 250-255.
3. Chandran K, Stein LY, Klotz MG, van Loosdrecht MCM. Nitrous oxide production by lithotrophic ammonia-oxidizing bacteria and implications for engineered nitrogen-removal systems. *Biochem Soc Trans* 2011; 39: 1832-1837.
4. Williams HC, Dellavalle RP, Garner S. Acne vulgaris. *The Lancet* 2012; 379(9813): 361-372. doi:10.1016/S0140-6736(11)60321-8.
5. Weller R, Price RJ, Ormerod AD, Benjamin N, & Leifert C. Antimicrobial effect of acidified nitrite on dermatophyte fungi, Candida and bacterial skin pathogens. *Journal of Applied Microbiology* 2001; *90*(4): 648-652.
6. Rico J, Quiring J, Hollenbach S, Enloe C, & Stasko N. Phase 2 study of efficacy and safety of SB204 in the treatment of acne vulgaris. *J Invest Dermatology* 2014; 134(8): S8.
7. Williams H, Robertson C, Stewart A, et al. Worldwide variations in the prevalence of symptoms of atopic eczema in the international study of asthma and allergies in childhood. *J Allergy Clin Immunol* 1999; 103: 125–138.
8. Diepgen, TL. Is the prevalence of atopic dermatitis increasing? In: Williams HC ed. Atopic Dermatitis: the Epidemiology, Causes and Prevention of Atopic Eczema. 2000 Cambridge: Cambridge University Press.
9. Roekevisch E, Spuls PI, Kuester D, Limpens J, Schmitt J. Efficacy and safety of systemic treatments for moderate-to-severe atopic dermatitis: a systematic review. *J Allergy Clin Immunol* 2014; 133(2):429-38.
10. Kobayashi T, Glatz M, Horiuchi K, Kawasaki H, Akiyama H, Kaplan DH, Kong HH, Amagai M, Nagao K. Dysbiosis and Staphylococcus aureus Colonization Drives Inflammation in Atopic Dermatitis. *Immunity* 2015; 42(4):756-66.

APPENDIX A: SCHEDULE OF EVENTS

Visit Name	Screening	Washout phase	Baseline Day 0	Day 5	Day 14 (Week 2)	Day 28 (Week 4)	Day 56 (Week 8)	Early Termination Visit	Comments/Clarification
Visit Number	V1		V2		V3	V4	V5		
Visit Window in days	-21 to -14	-14 to -1	0	+/-2	+/-2	+/-2	+/-3		
Informed Consent	X								Informed consent will occur prior to any protocol-mandated procedures, including the stopping of any excluded therapies.
Inclusion / Exclusion Criteria	X		X						
Demographics	X								
Medical History	X		X						
Concomitant Medications	X		X		X	X	X	X	
Physical Exam	X						X	X	
Vital Signs	X		X		X	X	X	X	Height and weight will be assessed at screening; BP, pulse, respiration rate, and

									temperature will be assessed at each visit. Smoking status will be recorded at Screening
eDiary	X	X	X	X	X	X	X	X	Subjects will be provided eDiary to be completed at home daily and in-clinic. At home eDiary will include WI-NRS, AI-NRS, rescue medication, dose administration, and local skin tolerability. In clinic eDiary will include POEM and 5-D Pruritus Scale. eDiary will be reviewed at the baseline visit to determine eligibility.
POEM	X		X		X	X	X	X	Patient reported assessment by ePRO
5-D Pruritus Scale	X		X		X	X	X	X	Patient reported assessment by ePRO
IGA	X		X		X	X	X	X	Clinician assessment by eCOA
EASI	X		X		X	X	X	X	Clinician assessment by eCOA
Itch Numeric Rating Scale (NRS)	X	X	X	X	X	X	X	X	Patient reported assessment by ePRO
Clinical Labs	X					X		X	Patients should fast for at least 8 hours before the test but optional at screening. Blood and urine for clinical

								(if visit occurs before Day 28)	chemistry will be shipped to the central lab for processing. Chemistry, Hematology, Lipid Panel, and Urinalysis will be done at Screening and Day 28. Serology will only be done at Screening. Kits and lab manuals will be provided by a central lab.
Urine pregnancy test for WOCBP	X		X			X	X	X	
IWRS			X						
Investigational Product compliance			X		X	X		X	Weights of dispensed and collected bottles to be recorded following instructions provided to sites.
Dispense study drug			X		X				
Application of study drug			X	X	X	X			First application of study drug will occur in the office under the supervision of the study staff.
Phone call to subjects		X		X					Call to initiate washout phase will be placed once results of screening clinical

									labs are finalized. Call on Day 5 will be made to counsel subjects on the use of IP and eDiary recordings, as well as answer any questions.
Collect study drug					X	X		X	
Study counseling	X	X	X	X	X	X			Subjects will be counseled at each visit on the appropriate use of IP, eDiary entries and use.
AE monitoring	X		X		X	X	X	X	AE will be monitored from screening to follow-up. After informed consent, but prior to study drug administration, only SAEs caused by a protocol-mandated intervention will be collected

APPENDIX A continued:

Schedule of eDiary and Efficacy Assessments

eDiary (ePRO) is provided to subjects at the screening visit for daily entry at home (e.g., application on smartphone). Questionnaires and clinician assessments are recorded via ePRO/eCOA (e.g., tablet) or CRF during the site visits per schedule of events. Additional patient reported information include rescue medication use, dose administration confirmation, and adjunctive therapy use.

Device	Assessment	Frequency and Duration of Assessment
eDiary	WI-NRS	Once daily from Screening visit through the Follow-up visit
eDiary	AI-NRS	Once daily from Screening visit through the Follow-up visit
eDiary	Local Tolerability	Once daily from Day 1 to Day 7 of treatment
eDiary	POEM	Patient reported at site visits
eDiary	5-D Pruritus Scale	Patient reported at site visits
eCOA/CRF	IGA	Clinician assessment at site visits
eCOA/CRF	EASI	Clinician assessment at site visits

APPENDIX B: EXCLUDED MEDICATIONS/THERAPY

Excluded medication/therapy is listed below. The use of an excluded medication/therapy is a protocol violation and must be recorded in the CRF.

Within 4 weeks prior to Baseline through the Follow-up period

- Systemic immunosuppressive/immunomodulating drugs (i.e., methotrexate, cyclosporine, etc.).
- Immunoglobulin or blood products.
- Systemic corticosteroids (oral, IV, injectable)
- NK1-R antagonists
- Class III or higher topical corticosteroids or any topical anti-pruritic therapies (other than stable doses of low- or mid-potency topical corticosteroids or bland emollients)
- Systemic therapies with recognized anti-pruritic (e.g. tricyclic antidepressants, sedatives, tranquilizers, gabapentin, marijuana or other cannabinoids, opioid receptor agonists/antagonists) or pruritic (e.g. opioids, angiotensin-converting enzyme inhibitors, cocaine, antimalarials) properties
    - Stable doses of oral H1 antihistamines will be permitted
- Systemic immunosuppressive/immunomodulatory therapies (including but not limited to PDE4 inhibitors, cyclosporine, mycophenolate-mofetil, methotrexate, azathioprine, interferon-gamma, or phototherapy)
- Allergen immunotherapy
- Any investigational therapy
- Strong CYP3A4 inhibitors, such as
    - boceprevir
    - clarithromycin
    - cobicistat
    - conivaptan
    - danoprevir and ritonavir
    - diltiazem
    - elvitegravir and ritonavir
    - regular grapefruit juice consumption
    - idelalisib
    - indinavir and ritonavir
    - itraconazole
    - ketoconazole
    - lopinavir and ritonavir
    - nefazodone
    - nelfinavir
    - paritaprevir and ritonavir and (ombitasvir and/or dasabuvir)
    - posaconazole
    - ritonavir

- saquinavir and ritonavir
- telaprevir
- tipranavir and ritonavir
- troleandomycin
- voriconazole

Within 2 weeks prior to Baseline through the Follow-up period

- High-potency topical corticosteroids (Class 1-3). The use of low- to mid-potency topical corticosteroids (Class 4-7), inhaled corticosteroids, or intranasal corticosteroids will be allowed.
- Use of crisaborole ointment.
- Systemic antibiotics.
- Bleach baths or topical coal tar.
- Topical calcineurin inhibitor use (e.g., pimecrolimus, tacrolimus).
- New onset use of systemic antihistamines.
- UVA or UVB phototherapy
- Topical and oral antibiotics/antiviral/antifungal/antiseptic agents
- Topical probiotics
- Topical antihistamines.
- The use of intranasal and oral antihistamines will be allowed[a].

a Subjects following stable regimens (≥2 weeks consistent for intranasal and 3 months for oral use before study baseline) with systemic antihistamines are permitted to continue use but should not alter the dose or stop the regimen while in the study within 1 week prior to Baseline.

Biologics

- Cell-depleting agents, including but not limited to rituximab: within 6 months of baseline.
- Infliximab, adalimumab, golimumab, certolizumab pegol, abatacept, etanercept, anakinra, and dupilumab: within 12 weeks of baseline, or 5 half-lives, whichever is longer.
- Other biologics: within 12 weeks of Baseline, or 5 half-lives (if known), whichever is longer.

APPENDIX C: EASI SCORE

How to Use EASI

The EASI scoring system uses a **defined process** to grade the **severity of the signs** of eczema and the **extent affected**:

1. Select a body region

Four body regions are considered separately:

- Head and neck
- Trunk (including the genital area)
- Upper extremities
- Lower Extremities (including the buttocks)

2. Assess the extent of eczema in that body region

Each body region has potentially 100% involvement. Using the table below, give each respective body region a **score of between 0 and 6** based on the percentage involvement. Precise measurements are not required.

% involvement	0	1-9%	10 - 29%	30 - 49%	50 - 69%	70 - 89%	90 - 100%
**Region score**	0	1	2	3	4	5	6

To aid in your body region grading you can use the **diagrams** in Appendix 1.

3. Assess the severity of each of the four signs in that body region:

1. Erythema
2. Edema/papulation
3. Excoriation
4. Lichenification

Further explanations of these terms can be found in FAQ's (Appendix 4)

Grade the severity of each sign on a scale of 0 to 3:

0	None
1	Mild
2	Moderate
3	Severe

- ✓ Take an average of the severity across the involved region.
- ✓ Half points may be used e.g. 2.5.
- ✓ Palpation may be useful in assessing edema/papulation as well as lichenification

To aid your severity grading, a **photographic atlas** of suggested categories is available in **Appendix 2**

Remember: ***Include only inflamed areas*** *in your assessment; do not include xerosis (dryness), ichthyosis, keratosis pilaris, urticaria, infection (unless there is underlying eczema), or post inflammatory pigmentation changes.*

EASI guidance December 14

How to record your scores

The assessed parameters are inserted into a table (example shown below for age≥8 years). The final EASI score ranges from 0-72.

Body region	Erythema	Edema/ papulation	Excoriation	Lichenification	Area score	Multiplier	Score
Head/neck	(+	+	+	)	x	X 0.1	
Trunk	(+	+	+	)	x	x 0.3	
Upper extremities	(+	+	+	)	x	x 0.2	
Lower extremities	(+	+	+	)	x	x 0.4	
The final EASI score is the sum of the 4 region scores							-------- (0-72)

Two forms of the EASI scoring system are available depending on the age of the patients. The multipliers for the region score are different in the under 8's version to reflect the relative proportion of body regions in young children:

- Patients 8 years or above
- Patients under 8 years of age.

The forms can be found in appendix 3.1 and 3.2 and also as word documents on the HOME website (www.homeforeczema.org)

EASI guidance December 14

Appendix 1: Eczema Area and Severity Index (EASI) - Extent of eczema per body region

Score **each region** from 0 to 100%

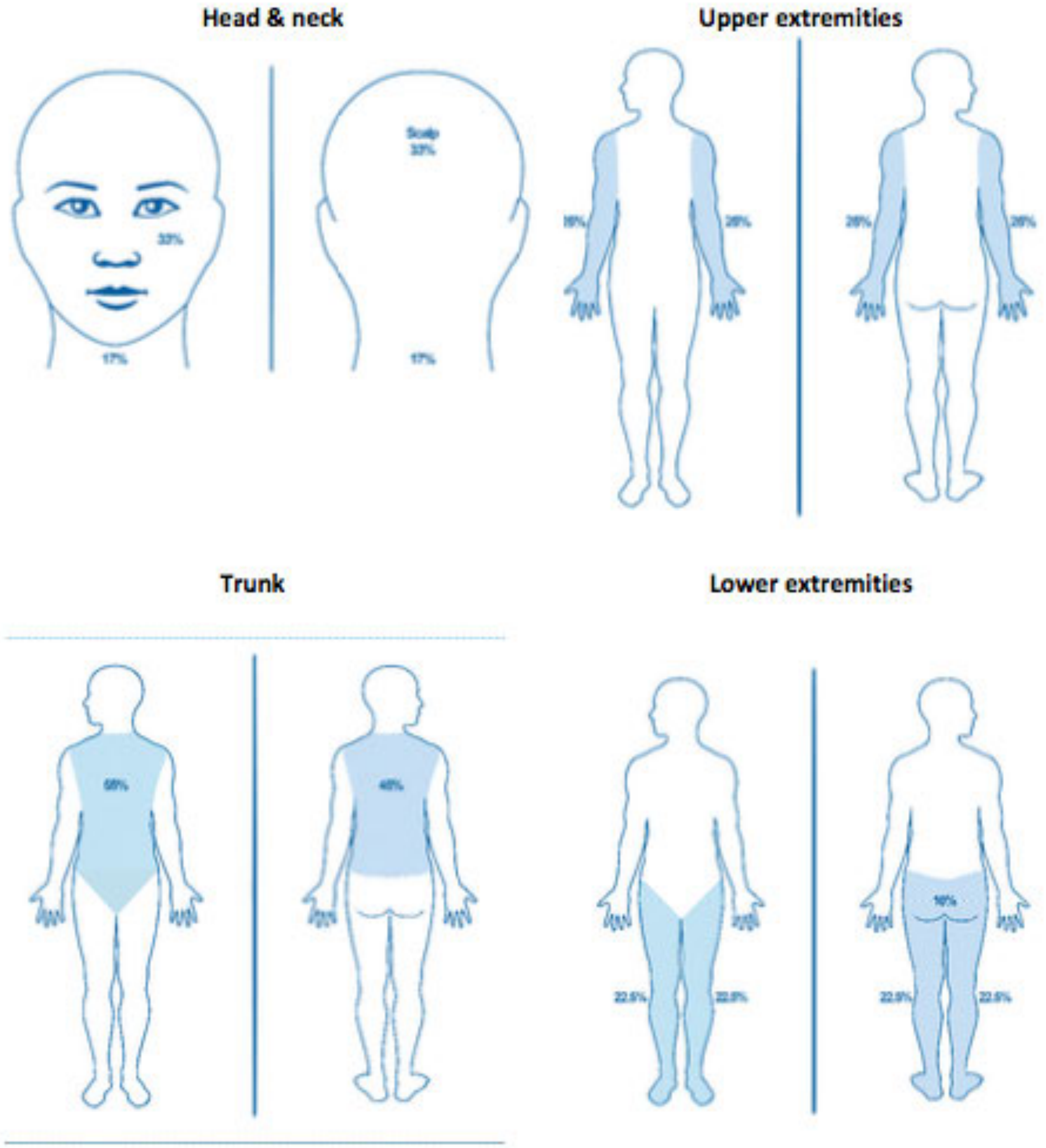

EASI guidance December 14

Appendix 2: Eczema Area and Severity Index (EASI) –lesion severity atlas

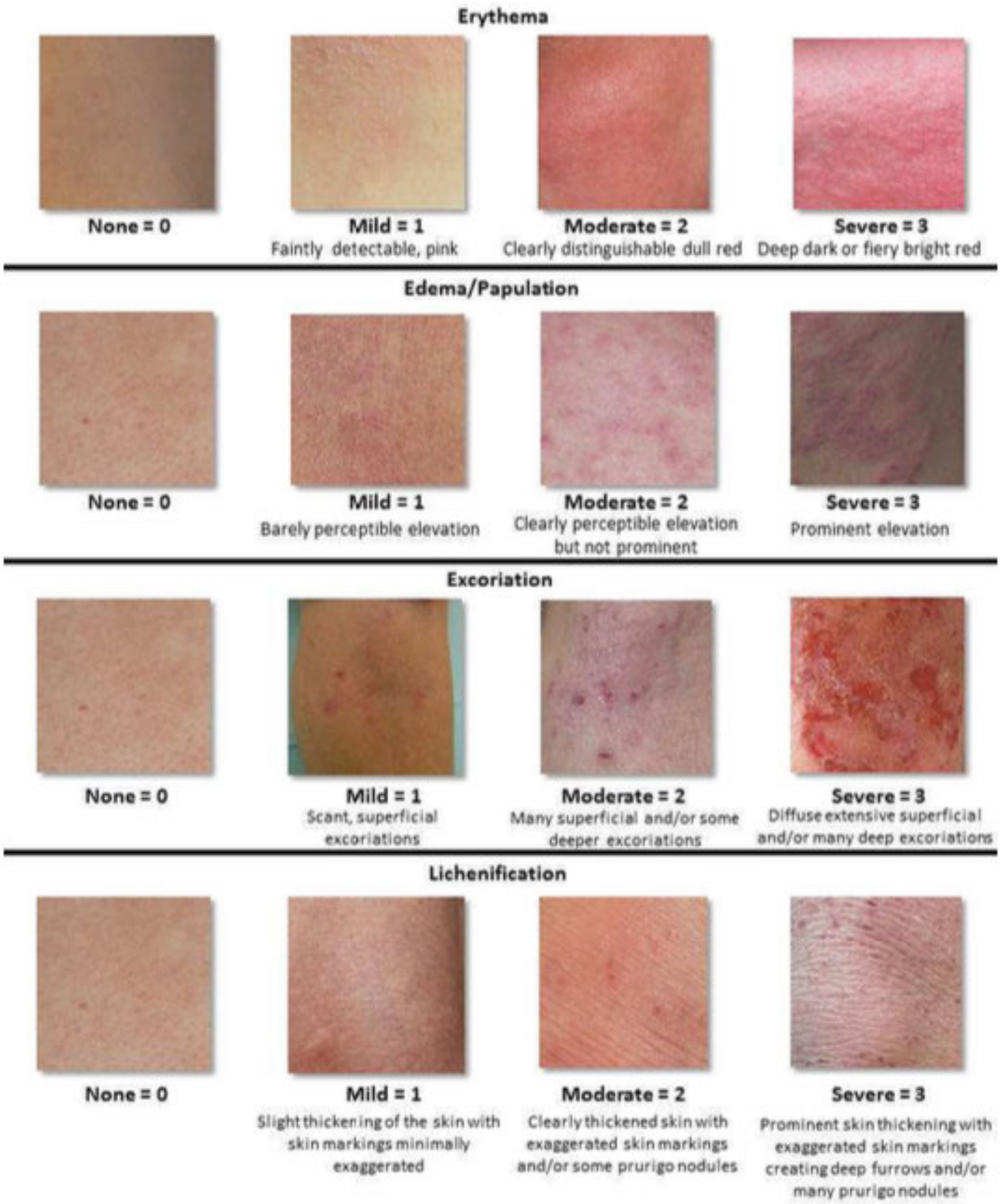

EASI guidance December 14

Appendix 3.1: Eczema Area and Severity Index (EASI) case report form – age <8 years

Area of Involvement: Each body region has potentially 100% involvement. Score **0 to 6** based on the following table:

% involvement	0	1-9%	10 - 29%	30 - 49%	50 - 69%	70 - 89%	90 - 100%
Region score	0	1	2	3	4	5	6

Severity of Signs: Grade the severity of each sign on a scale of **0 to 3**:

0	None
1	Mild
2	Moderate
3	Severe

- ✓ Take an average of the severity across the involved area.
- ✓ Half points may be used e.g. 2.5.

Scoring table:

Body region	Erythema (0-3)	Edema/ Papulation (0-3)	Excoriation (0-3)	Lichenification (0-3)	Region score (0-6)	Multiplier	Score per body region
Head/neck	(+	+	+	)	X	X 0.2	
Trunk	(+	+	+	)	X	X 0.3	
Upper extremities	(+	+	+	)	X	X 0.2	
Lower extremities	(+	+	+	)	X	X 0.3	
The final EASI score is the sum of the 4 region scores:							____________ (0-72)

EASI guidance December 14

Appendix 3.2: Eczema Area and Severity Index (EASI) case report form - age≥8 years

Area of Involvement: Each body area has potentially 100% involvement. Score **0 to 6** based on the following table:

% involvement	0	1-9%	10 - 29%	30 - 49%	50 - 69%	70 - 89%	90 - 100%
Region score	0	1	2	3	4	5	6

Severity of Signs: Grade the severity of each sign on a scale of **0 to 3**:

0	None
1	Mild
2	Moderate
3	Severe

- ✓ Take an average of the severity across the involved area.
- ✓ Half points may be used e.g. 2.5.

Scoring table:

Body region	Erythema (0-3)	Edema/ Papulation (0-3)	Excoriation (0-3)	Lichenification(0-3)	Region score (0-6)	Multiplier	Score per body region
Head/neck	(+	+	+	)	x	X 0.1	
Trunk	(+	+	+	)	x	X 0.3	
Upper extremities	(+	+	+	)	x	X 0.2	
Lower extremities	(+	+	+	)	x	X 0.4	
The final EASI score is the sum of the 4 region scores:							________ (0-72)

EASI guidance December 14

<u>Appendix 4</u> - Frequently Asked Questions

What is the difference between edema/papulation and lichenification?
Consider edema/papulation as corresponding to the acute signs of atopic dermatitis that reflect histological spongiosis. Lichenification are more firm thickened plaques with accentuation of the skin markings that develop as a result of prolonged scratching or rubbing in chronic disease. In darker skin types, follicular lichenification may present as firm flat-topped discrete papules. Grade these chronic lesions as lichenification.

How do I grade prurigo nodules?
Prurigo nodules are larger, deeper lesions as a result of chronic scratching and are graded as areas of lichenification.

How do I grade erythema in darker skin?
To avoid underestimating inflammation in patients with darker skin tones, take into account the underlying skin pigment when grading erythema. Often this means increasing your erythema grade by one level.

Can half-steps be used to assess lesion severity?
The original EASI validation study allowed for half steps. These may be helpful when trying to average the severity of a parameter over a region. For example, if there are some areas with an erythema grading of 2 and some areas more consistent with a severity of 3, 2.5 may be a good choice.

What if most areas in a region are a severity grade of 1, but there are some areas that are a grade 3?
Attempt to average the severity across the involved areas in that region. If these areas are close to equal in size, a score of 2 would be most appropriate. If the majority of involved areas are a grade 1, a score of 1 or 1.5 is more appropriate. Be careful not to score the highest severity in a region but the average one.

How do I grade xerosis (dryness), ichthyosis and hyperlinear palms?
Unless there is active acute or chronic eczema overlying these findings, they are not included in the EASI assessment.

How precise should my assessment of eczema extent be?
The *region scores*, which reflect the extent of eczema, were designed and validated as rough estimates of the percentage of involved skin. Each region is given a *score* ranging from 0 to 6, based on a “ballpark” estimation of extent (see region score table in page 1). If you find it difficult to provide a rough estimate of disease extent, you can use the schematics in Appendix 1 to guide you. More time-consuming methods for evaluating disease extent such as the rule of nines or the ‘palm’ method are generally unnecessary, as the EASI was designed to be…easy.

EASI guidance December 14

My patient has responded well to treatment and significantly improved since the last visit. Should I adjust the grading based on the patient's relative improvement?
No. The EASI is a static score, meaning that it is done independently at each time point to reflect current severity. You should grade the EASI per visit regardless of the previous status. Studies have shown that the EASI score has good responsiveness, meaning that overall it is sensitive to change and the improvement will be reflected in the total score.

Can the EASI be used in children?
Yes. The EASI is performed in the same method in all age groups, but the calculation of the final EASI score differs by age. When calculating the EASI, each of the 4 region scores is multiplied by a constant which reflects the relative contribution of that region to the total body surface area. For patients 8 years and older the multipliers are 0.1 for the head/neck, 0.2 for the upper extremities, 0.3 for the trunk and 0.4 for the lower extremities. Below 8 years of age the head/neck multiplier is increased to 0.2 while the lower extremities multiplier decreases to 0.3, consistent with the proportions of these regions in childhood. Refer to Appendix 3 for EASI forms by age.

What happens if a child turns 8 during the course of the study? Which EASI formula should I use?
There are no clear-cut definitions for this situation. In general, if the study is a short term study such as an RCT lasting a few months – using the same formula throughout the trial is preferred, even if the child turns 8 during these months. Keeping the EASI formula consistent in this scenario can serve to preserve the EASI sensitivity to change (e.g. its change in response to treatment) without compromising the validity of the score.
In long term studies such as cohort studies lasting a year or longer, it is important to update the EASI formula based on the physical changes children go through. Switching to the age 8+ formula once a child is older is preferred in that scenario.

What do the terms erythema, edema/papulation, excoriation and lichenification mean?
These are key signs of atopic dermatitis. Recognizing and grading them properly requires training on the visual and physical exam consistent with these signs. Generally speaking, erythema is skin redness; edema/papulation refers to an elevation or swelling of the skin (that should be differed from lichenification below); excoriations are scratch marks that have broken the skin surface; and lichenification is a leathery thickening of the skin with exaggerated skin markings.

APPENDIX D: PATIENT ORIENTED ECZEMA MEASURE (POEM)

POEM
Patient-Oriented Eczema Measure

The University of Nottingham
UNITED KINGDOM · CHINA · MALAYSIA

POEM for self-completion

Patient Details: ____________________

Date: ____________________

Please circle one response for each of the seven questions below about your eczema. Please leave blank any questions you feel unable to answer.

1. Over the last week, on how many days has your skin been itchy because of your eczema?

   No days   1-2 days   3-4 days   5-6 days   Every day

2. Over the last week, on how many nights has your sleep been disturbed because of your eczema?

   No days   1-2 days   3-4 days   5-6 days   Every day

3. Over the last week, on how many days has your skin been bleeding because of your eczema?

   No days   1-2 days   3-4 days   5-6 days   Every day

4. Over the last week, on how many days has your skin been weeping or oozing clear fluid because of your eczema?

   No days   1-2 days   3-4 days   5-6 days   Every day

5. Over the last week, on how many days has your skin been cracked because of your eczema?

   No days   1-2 days   3-4 days   5-6 days   Every day

6. Over the last week, on how many days has your skin been flaking off because of your eczema?

   No days   1-2 days   3-4 days   5-6 days   Every day

7. Over the last week, on how many days has your skin felt dry or rough because of your eczema?

   No days   1-2 days   3-4 days   5-6 days   Every day

Total POEM Score (Maximum 28): ....

© The University of Nottingham

POEM

Patient-Oriented Eczema Measure

The University of Nottingham
UNITED KINGDOM · CHINA · MALAYSIA

POEM for self-completion

How is the scoring done?

Each of the seven questions carries equal weight and is scored from 0 to 4 as follows:

No days	= 0
1-2 days	= 1
3-4 days	= 2
5-6 days	= 3
Every day	= 4

Note:

- If one question is left unanswered this is scored 0 and the scores are summed and expressed as usual out of a maximum of 28
- If two or more questions are left unanswered the questionnaire is not scored
- If two or more response options are selected, the response option with the highest score should be recorded

What does a poem score mean?

To help patients and clinicians to understand their POEM scores, the following bandings have been established (see references below):

• 0 to 2	= Clear or almost clear
• 3 to 7	= Mild eczema
• 8 to 16	= Moderate eczema
• 17 to 24	= Severe eczema
• 25 to 28	= Very severe eczema

Do I need permission to use the scale?

Whilst the POEM scale is protected by copyright, it is freely available for use and can be downloaded from: www.nottingham.ac.uk/dermatology
We do however ask that you register your use of the POEM by e-mailing cebd@nottingham.ac.uk with details of how you would like to use the scale, and which countries the scale will be used in.

References

Charman CR, Venn AJ, Williams HC. The Patient-Oriented Eczema Measure: Development and Initial Validation of a New Tool for Measuring Atopic Eczema Severity From the Patients' Perspective. Arch Dermatol. 2004;140:1513-1519

Charman CR, Venn AJ, Ravenscroft JC, Williams HC. Translating Patient-Oriented Eczema Measure (POEM) scores into clinical practice by suggesting severity strata derived using anchor-based methods. Br J Dermatol. Dec 2013; 169(6): 1326–1332.

© The University of Nottingham. The Patient Oriented Eczema Measure (POEM) scale is free to use. Permission is granted to reproduce and/or redistribute this material in its entirety without modification. Any use which falls outside this remit requires the express consent of the copyright owner.

APPENDIX E: IGA

Score	Category	Definition
0	Clear	Minor residual discoloration, no erythema or induration/papulation, no oozing/crusting
1	Almost Clear	Trace faint pink erythema with almost no induration/papulation and no oozing/crusting
2	Mild Disease	Faint pink erythema with mild induration/papulation and no oozing/crusting
3	Moderate Disease	Pink-red erythema with moderate induration/papulation and there may be some oozing/crusting
4	Severe Disease	Deep/bright red erythema with severe induration/papulation with oozing/crusting

APPENDIX F: 5-D PRURITUS SCALE

5-D Pruritus Scale

1. **Duration**: During the last 2 weeks, how many hours a day have you been itching?

Less than 6hrs/day	6-12 hrs/day	12-18 hrs/day	18-23 hrs/day	All day
☐ 1	☐ 2	☐ 3	☐ 4	☐ 5

2. **Degree**: Please rate the intensity of your itching over the past 2 weeks

Not present	Mild	Moderate	Severe	Unbearable
☐ 1	☐ 2	☐ 3	☐ 4	☐ 5

3. **Direction**: Over the past 2 weeks has your itching gotten better or worse compared to the previous month?

Completely resolved	Much better, but still present	Little bit better, but still present	Unchanged	Getting worse
☐ 1	☐ 2	☐ 3	☐ 4	☐ 5

4. **Disability**: Rate the impact of your itching on the following activities over the last 2 weeks

	Never affects sleep	Occasionally delays falling asleep	Frequently delays falling asleep	Delays falling asleep and occasionally wakes me up at night	Delays falling asleep and frequently wakes me up at night
**Sleep**	☐ 1	☐ 2	☐ 3	☐ 4	☐ 5

	N/A	Never affects this activity	Rarely affects this activity	Occasionally affects this activity	Frequently affects this activity	Always affects this activity
**Leisure/Social**	☐	☐ 1	☐ 2	☐ 3	☐ 4	☐ 5
**Housework/ Errands**	☐	☐ 1	☐ 2	☐ 3	☐ 4	☐ 5
**Work/School**	☐	☐ 1	☐ 2	☐ 3	☐ 4	☐ 5

5. **Distribution:** Mark whether itching has been present in the following parts of your body over the last 2 weeks. If a body part is not listed, choose the one that is closest anatomically.

	Present		Present
Head/Scalp	☐	Soles	☐
Face	☐	Palms	☐
Chest	☐	Tops of Hands/Fingers	☐
Abdomen	☐	Forearms	☐
Back	☐	Upper Arms	☐
Buttocks	☐	Points of Contact w/ Clothing (e.g waistband, undergarment)	☐
Thighs	☐	Groin	☐
Lower legs	☐		
Tops of Feet/Toes	☐		

APPENDIX G: PATIENT REPORTED LOCAL TOLERABILITY

PATIENT ASSESSMENT OF LOCAL SKIN TOLERABILITY AT APPLICATION SITE(S)

SUBJECT AGE RANGE: 12 years old and above

INSTRUCTIONS:

Please examine all of your skin lesions that are treated with B244 topical solution. Please circle one score for each category that best describes the changes you observed in all skin lesions. For each day of the first 7 days of treatment, please examine all skin lesions at approximately the same time every day.

DAY OF TREATMENT (1-7): _________________

TIME OF DAY: _____________AM or PM

**SKIN REDNESS AND/OR COLOR CHANGE AT APPLICATION SITE**		
Score	Grade	Definition
0	None	No new redness or new color change; no new increase in redness or new increase in color change
1	Mild	Slight increase in redness or slight increase in color change
2	Moderate	Increase in redness or increase in color change
3	Severe	Intense redness or intense color change

ITCHING AT APPLICATION SITE		
Score	Grade	Definition
0	None	No new itching or new scratching
1	Mild	Slight increase in itching or slight increase in scratching
2	Moderate	Increase in itching or increase in scratching that is not disturbing sleep
3	Severe	Increase in itching or increase in scratching that is disturbing sleep

BURNING AND/OR STINGING AT APPLICATION SITE		
Score	Grade	Definition
0	None	No new burning or new stinging
1	Mild	Slight increase in warm or tingling sensation that is not bothersome
2	Moderate	Increase in warm or tingling sensation that is bothersome
3	Severe	Hot or stinging sensation that is causing definite discomfort

PAIN AND/OR TENDERNESS AT APPLICATION SITE		
Score	Grade	Definition
0	None	No new pain or new tenderness
1	Mild	Slight increase in pain or slight increase in tenderness that is not bothersome
2	Moderate	Increase in pain or increase in tenderness that is bothersome
3	Severe	Intense pain or intense tenderness causing definite discomfort or disturbing sleep

NEW OR CHANGING RASH AT APPLICATION SITE		
Score	Grade	Definition
0	None	No new rash or no change in rash (such as swelling, fullness, or blistering)
1	Mild	Slight new rash or slight increase in rash (such as swelling, fullness, or blistering) that is not bothersome
2	Moderate	New rash or increase in rash (such as swelling, fullness, or blistering) that is bothersome
3	Severe	Significant new rash or significant increase in rash (swelling, fullness, or blistering) causing discomfort

STATISTICAL ANALYSIS PLAN
PHASE II

VERSION: 3.0

DATE:

January 12, 2022

BASED ON:

Protocol Version Amendment 3 Date: 28-FEB-2020

Data Management Plan Final Version 2 Date: 10-JUL-2020

Study Drug:

B244 Topical application

Protocol Number:

PRB244-01

Study Title:

A Phase II, Randomized, Double-Blind, Vehicle Controlled Study of the Efficacy, Safety, and Tolerability of B244 Topical Spray for the Treatment of Pruritus in Adults with a History of Atopic Dermatitis

Sponsor:

AOBiome, LLC
125 Cambridgepark Drive
Cambridge, MA 02140

STATISTICAL ANALYSIS PLAN REVIEW AND APPROVAL

This Statistical Analysis Plan has been prepared in accordance with team reviewers' specifications.

Prepared by: [redacted]

12 January 2022

[redacted] Date

Sponsor Review: [redacted]

12 January 2022

[redacted] Date

[redacted]

12 January 2022

[redacted] Date

Table of Contents

STATISTICAL ANALYSIS PLAN REVIEW AND APPROVAL 2
1 LIST OF ABBREVIATIONS 6
2 INTRODUCTION 8
2.1 Study Flowchart 8
2.2 Schedule of Activities 9
2.3 Schedule of eDiary and Efficacy Assessments 12
3 STUDY OBJECTIVE AND ENDPOINTS 13
3.1 Study Objectives 13
3.1.1 Primary Objective 13
3.1.2 Secondary Objectives 13
3.2 Study Endpoints 13
3.2.1 Efficacy - Primary Endpoint 13
3.2.2 Efficacy - Secondary Endpoints 13
3.2.3 Efficacy - Exploratory Endpoints 13
3.2.4 Safety & Tolerability 14
4 STUDY DESIGN 14
4.1 General Study Design and Plan 14
4.1.1 Inclusion-Exclusion Criteria and General Study Population 15
4.2 Study Withdrawal and Withdrawal from Investigational Product and Stopping Criteria 17
4.3 Early Termination 18
5 PLANNED ANALYSIS 18
5.1 Final Analysis 18
6 GENERAL CONSIDERATIONS 18
6.1 General Summary Tables and Individual Subject Data Listings 18
6.2 Data Management 19
6.3 Data Presentation Conventions 19
6.4 Baseline Definition 20
6.5 Derived and Transformed Data 21
6.5.1 Baseline Age 21

6.5.2 Study Day 21
6.5.3 Weekly Average WI-NRS and AI-NRS 22
6.5.4 Derivation of ≥4 Point and Any Improvement in WI-NRS and AI-NRS 23
6.5.5 Percent Change from Baseline 23
6.5.6 Rescue Medication During Treatment Period 24
6.5.7 Missing Start and Stop Dates for Prior and Concomitant Medication 24
6.5.8 Safety Parameters 24
6.5.9 Missing Start and Stop Dates for Adverse Events 25
6.5.10 Visit Windows 26
6.5.11 Categorization of Continuous Variables 26
6.5.12 Site Related Baseline Covariates 26
7 STATISTICAL ANALYSES 27
7.1 Sample Size Determination 27
7.2 Analysis Populations 27
7.2.1 Screen Failures 27
7.2.2 Subjects Enrolled and Subjects Not Assigned 28
7.2.3 Safety Population 28
7.2.4 Modified ITT Population 28
7.2.5 Per-Protocol (PP) Population 28
7.2.6 Role of Populations in Analyses 28
7.3 General Analysis 28
7.3.1 Subjects Disposition 28
7.3.2 Clinical Trial Protocol Deviations 29
7.3.3 Demographic and Baseline Characteristics 29
7.4 Efficacy Analysis 29
7.4.1 Analysis of WI-NRS and AI-NRS 32
7.4.2 Analysis of Primary Endpoint of Change from Baseline to Week 4 in Weekly Average WI-NRS 32
7.4.3 Analysis of IGA, EASI, POEM, 5-D Pruritus Scale 33
7.5 Safety Analysis 33
7.5.1 Study Drug Exposure 33
7.5.2 Adverse Events 34
7.5.3 Prior and Concomitant Medications 35

7.5.4 Rescue Medications ... 35
7.5.5 Patient Reported Local Tolerability ... 36
7.5.6 Laboratory Data ... 36
7.5.7 Vital Signs and Physical Examination ... 37
7.5.8 Medical History ... 37
7.5.9 COVID-19 Issues ... 37
7.6 Handling of Dropouts or Missing Data ... 37
7.7 Changes to Planned Analyses ... 37
8 REFERENCES ... 38

1 LIST OF ABBREVIATIONS

Abbreviation	Full Form
AD	Atopic Dermatitis
ADaM	Analysis Data Model
AE	Adverse Event
AI	Average Itch
AI-NRS	Average Itch – Numeric Rating Scale
AMO	Ammonia Monooxygenase
ANCOVA	Analysis of Covariance
AOB	Ammonia Oxidizing Bacteria
ATC	Anatomical Therapeutic Chemical Classification System
BID	Twice-Daily
BMI	Body Mass Index
BSA	Body Surface Area
CDISC	Clinical Data Interchange Standards Consortium
COA	Clinical Outcome Assessment
CRF	Case Report Form
EASI	Eczema Area and Severity Index
E/T	Early Termination
FDA	Food and Drug Administration
HAO	NH2OH oxidoreductase
HbsAg	Hepatitis B Virus Surface Antigen
HCV Ab	Hepatitis C Virus Antibody
HIV	Human Immunodeficiency Virus
ICH	International Conference on Harmonization
IEC	Independent Ethics Committee
IGA	Investigator Global Assessment
IP	Investigational Product
IRB	Institutional Review Board
ITT	Intention to Treat
IWRS	Interactive Web Response System
MedDRA	Medical Dictionary for Regulatory Activities
mITT	Modified Intention to Treat
NH2OH	Hydroxylamine
NH3	Ammonia
NO	Nitric oxide
NO2-	Nitrite
NRI	Non-Responder Imputation
NRS	Itch Numeric Rating Scale
PCP	Primary Care Physician
POEM	Patient Oriented Eczema Measure
PRO	Patient Reported Outcomes

PP	Per Protocol
PT	Preferred Term
SAE	Serious Adverse Event
SAP	Statistical Analysis Plan
SDTM	Study Data Tabulation Model
SOA	Schedule of Assessments
SOC	System Organ Class
SPM	Study Procedures Manual
TEAE	Treatment Emergent Adverse Event
TESAE	Treatment Emergent Serious Adverse Event
WI-NRS	Worst Itch – Numeric Rating Scale
WHO	World Health Organization
WOCBP	Women of Childbearing Potential

2 INTRODUCTION

This document describes the statistical methods and data presentations to be used in the summary and analysis of data from Protocol PRB244-01. Background information is provided for the overall study design and objectives. The study conduct and study details can be found in the protocol[1] and electronic case report forms (eCRFs).

2.1 Study Flowchart

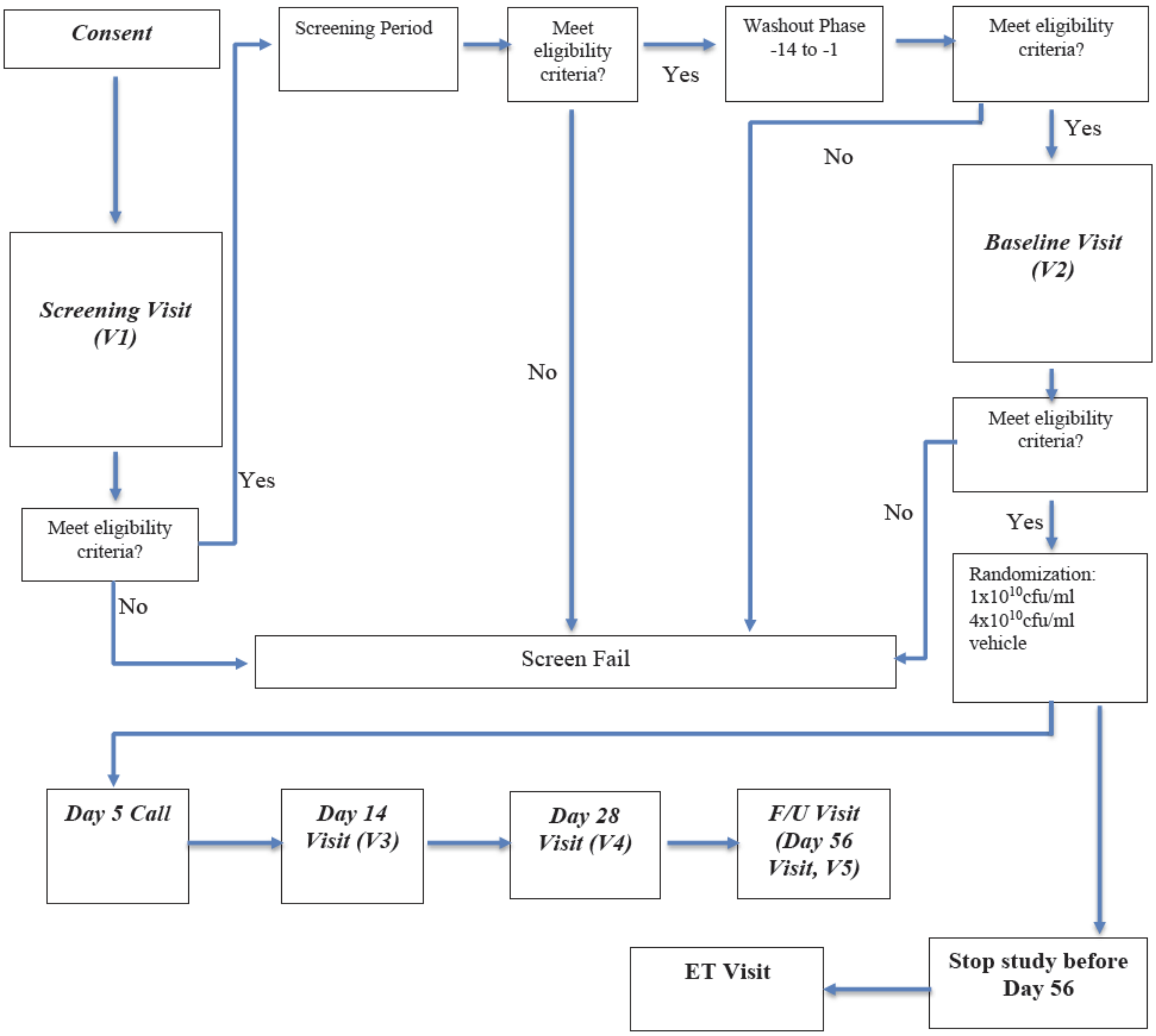

2.2 Schedule of Activities

Visit Name#	Screening	Washout phase	Baseline Day 0	Day 5	Day 14 (Week 2)	Day 28 (Week 4)	Day 56 (Week 8)	Early Termination Visit
Visit Number	V1		V2		V3	V4	V5	
Visit Window in days	-21 to -14	-14 to -1	0	+/-2	+/-2	+/-2	+/-3	
Informed Consent	X							
Inclusion / Exclusion Criteria	X		X					
Demographics	X							
Medical History	X		X					
Concomitant Medications	X		X		X	X	X	X
Physical Exam	X						X	X
Vital Signs	X		X		X	X	X	X
eDiary	X	X	X	X	X	X	X	X
POEM	X		X		X	X	X	X
5-D Pruritus Scale	X		X		X	X	X	X
IGA	X		X		X	X	X	X
EASI	X		X		X	X	X	X
Itch Numeric Rating Scale (NRS)	X	X	X	X	X	X	X	X
Clinical Labs	X					X		X*
Urine pregnancy test for WOCBP	X		X			X	X	X
IWRS			X					
Investigational Product compliance			X		X	X		X
Dispense study drug			X		X			
Application of study drug			X	X	X	X		
Phone call to subjects		X		X				
Collect study drug					X	X		X
Study counseling	X	X	X	X	X	X		

Visit Name#	Screening	Washout phase	Baseline Day 0	Day 5	Day 14 (Week 2)	Day 28 (Week 4)	Day 56 (Week 8)	Early Terminatio n Visit
Visit Number	V1		V2		V3	V4	V5	
Visit Window in days	-21 to -14	-14 to -1	0	+/-2	+/-2	+/-2	+/-3	
AE monitoring	X		X		X	X	X	X

#: Comments/clarification to visits in table below.
*: if visit occurs before Day 28

Visit Name	Comments/Clarification
Informed Consent	Informed consent will occur prior to any protocol-mandated procedures, including the stopping of any excluded therapies.
Vital Signs	Height and weight will be assessed at Screening; BP, pulse, respiration rate, and temperature will be assessed at each visit. Smoking status will be recorded at Screening
eDiary	Subjects will be provided eDiary to be completed at home daily and in-clinic. At home eDiary will include WI-NRS, AI-NRS, rescue medication, dose administration, and local skin tolerability. In clinic eDiary will include POEM and 5-D Pruritus Scale. eDiary will be reviewed at the Baseline visit to determine eligibility.
POEM	Patient reported assessment by ePRO
5-D Pruritus Scale	Patient reported assessment by ePRO
IGA	Clinician assessment by eCOA
EASI	Clinician assessment by eCOA
Itch Numeric Rating Scale (NRS)	Patient reported assessment by ePRO
Clinical Labs	Patients should fast for at least 8 hours before the test but optional at Screening. Blood and urine for clinical chemistry will be shipped to the central lab for processing. Chemistry, Hematology, Lipid Panel, and Urinalysis will be done at Screening and Day 28. Serology will only be done at Screening. Kits and lab manuals will be provided by a central lab.
Investigational Product compliance	Weights of dispensed and collected bottles to be recorded following instructions provided to sites.
Application of study drug	First application of study drug will occur in the office under the supervision of the study staff.
Phone call to subjects	Call to initiate washout phase will be placed once results of screening clinical labs are finalized. Call on Day 5 will be made to counsel subjects on the use of IP and eDiary recordings, as well as answer any questions.
Study counseling	Subjects will be counseled at each visit on the appropriate use of IP, eDiary entries and use.
AE monitoring	AE will be monitored from screening to follow-up. After informed consent, but prior to study drug administration, only SAEs caused by a protocol-mandated intervention will be collected

2.3 Schedule of eDiary and Efficacy Assessments

eDiary (ePRO) is provided to subjects at the screening visit for daily entry at home (e.g., application on smartphone). Questionnaires and clinician assessments are recorded via ePRO/eCOA (e.g., tablet) or CRF during the site visits per schedule of events. Additional patient reported information include rescue medication use, dose administration confirmation, and adjunctive therapy use.

Device	Assessment	Frequency and Duration of Assessment
eDiary	WI-NRS	Once daily from Screening visit through the Follow-up visit
eDiary	AI-NRS	Once daily from Screening visit through the Follow-up visit
eDiary	Patient Reported Local Tolerability	Once daily from Day 1 to Day 7 of treatment
eDiary	POEM	Patient reported at site visits
eDiary	5-D Pruritus Scale	Patient reported at site visits
eCOA/CRF	IGA	Clinician assessment at site visits
eCOA/CRF	EASI	Clinician assessment at site visits

3 STUDY OBJECTIVE AND ENDPOINTS

3.1 Study Objectives

3.1.1 Primary Objective

To assess the efficacy of B244 in the treatment of pruritus in adults with a history of atopic dermatitis.

3.1.2 Secondary Objectives

To assess the safety and tolerability of B244 in adults with a history of atopic dermatitis.

3.2 Study Endpoints

3.2.1 Efficacy - Primary Endpoint

The primary efficacy endpoint is:

- Mean change (absolute) in WI-NRS from Baseline to Week 4

3.2.2 Efficacy - Secondary Endpoints

The secondary efficacy endpoints are:

- Proportion of subjects with ≥4point improvement in WI-NRS from Baseline to Week 4
- Proportion of subjects with any improvement in WI-NRS from Baseline to Week 4
- Mean change (absolute and percent) in AI-NRS from Baseline to Week 4
- Proportion of subjects with ≥4 point improvement in AI-NRS from Baseline to Week 4
- Proportion of subjects with any improvement in AI-NRS from Baseline to Week 4
- Mean change (absolute and percent) in WI-NRS from Baseline to Week 2
- Proportion of subjects with ≥4 point improvement in WI-NRS from Baseline to Week 2
- Mean change (absolute and percent) in POEM from Baseline to Week 4
- Mean change (absolute and percent) in 5-D Pruritus Scale from Baseline to Week 4

The endpoints will be reported for all weeks or all visits. The primary endpoint of mean change in WI-NRS is also presented at all weeks for both absolute and percent change. Absolute change will be considered the primary endpoint with percent change as supportive. Any improvement in WI-NRS and AI-NRS is defined as ≥1 point improvement from Baseline.

3.2.3 Efficacy - Exploratory Endpoints

- Mean change (absolute and percent) in IGA from Baseline to Week 4
- Mean change (absolute and percent) in EASI from Baseline to Week 4

- Proportion of subjects with IGA of Clear or Almost Clear and ≥2 point improvement from Baseline to Week 4
- Proportion of subjects with IGA of Clear or Almost Clear at Week 4
- Proportion of subjects with any improvement in IGA from Baseline to Week 4
- Proportion of subjects with ≥50% Improvement in EASI (EASI-50) from baseline to Week 4
- Proportion of subjects with ≥75% Improvement in EASI (EASI-75) from baseline to Week 4
- Proportion of subjects with ≥90% Improvement in EASI (EASI-90) from baseline to Week 4.

The endpoints will be reported for all visits. Absolute change will be considered the primary endpoint with percent change as supportive. Any improvement in IGA is defined as ≥1 point improvement from Baseline.

3.2.4 Safety & Tolerability

Safety and tolerability endpoints include the following:

- Incidence of TEAEs and SAEs
- TEAEs leading to discontinuation
- Changes in vital signs and clinical laboratory parameters following study drug exposure
- Changes in local skin tolerability following application of study drug

4 STUDY DESIGN

4.1 General Study Design and Plan

This is a prospective, vehicle-controlled, double-blind, multicenter, randomized phase II trial, comparing the effect of twice daily B244 applications for 4 weeks vs vehicle applications on treatment of mild to moderate pruritus associated with atopic dermatitis.

Approximately 576 subjects are planned to be enrolled.

The total duration of the study will be approximately 11 weeks. Participants will report for a Screening visit and if all inclusion/exclusion criteria are met, subjects will go through a two-week washout phase before reporting for a Baseline visit.

After Screening and Baseline, participants will be randomized to one of two doses of B244 or vehicle application for 4 weeks.

Randomization will be 1:1:1 so that an equal number of patients will be treated in each arm of the study.

All B244 randomized subjects will be treated at the dose of 1×10^{10} cells/ml (O.D. 5.0) or 4×10^{10} cells/ml (O.D. 20.0).

Subjects must be willing and able to complete diary within a consistent time frame on a daily basis and to comply with restrictions on allowable therapies for the duration of the study.

All subjects will attend a Screening visit not more than 21 days prior to Baseline (Day 0).

Subjects will be required to return to the clinic at Baseline, Day 14 (Week 2) and Day 28 (Week 4) visits. All subjects will be asked to attend a Week 8 follow-up visit 4 weeks (28 (±3) days) after the last dose of study medication.

Subjects will apply a total of 10 pumps of IP per application across all affected areas twice-a-day (i.e., 10 pumps in the morning and 10 pumps again at night) for 4 weeks.

Safety evaluations will consist of review of participant's medical history at screening and on-going assessment of adverse events reported throughout the study duration.

4.1.1 Inclusion-Exclusion Criteria and General Study Population

4.1.1.1 Inclusion Criteria

Deviations from inclusion criteria are not allowed because they can potentially jeopardize the scientific integrity of the study, regulatory acceptability, or participant safety. Therefore, adherence to the criteria as specified in the protocol is essential.

Participants eligible for enrollment in the study must meet all the following criteria:

1. Male and female subjects 18 to 65 years of age.
2. Pruritus of at least 4 weeks duration prior to the initial Screening visit and during the 2 week washout period.
    a. Subjects using stable doses of oral H1 antihistamines at the initial Screening visit must be willing to continue these at the same doses and frequencies throughout the study inclusive of the follow-up period.
3. Worst Itch Numeric Rating Scale (WI-NRS) score ≥ 7 in the 24-hour period prior to the initial Screening as well as Baseline visits.
4. Average weekly WI-NRS score ≥6 for each week of the washout period, as recorded in the eDiary.
5. A history of atopic dermatitis for greater than 12 months consistent with a diagnosis of atopic dermatitis, as defined by the 2014 American Academy of Dermatology (AAD) Guidelines of Care for the Management of Atopic Dermatitis[2].
    a. Subjects using bland emollients at the initial Screening visit will be allowed to continue to use their emollient of choice at a similar dose and frequency throughout the study, if used.

   b. Subjects using low- to mid-potency topical corticosteroids at the initial Screening visit will be allowed to use their topical corticosteroid of choice at the same dose and frequency no more than 7 days per month throughout the study as rescue medication.
6. A minimum of 10% and not more than 40% of the subjects' BSA affected by atopic dermatitis (*affected* is defined by physical examination findings: erythema, edema, scaling, lichenification, excoriation, with the excoriation serving as the physical examination correlate of pruritus) at Screening and Baseline.
   a. Subjects' BSA can include face and body OR body alone BUT NOT face alone.
7. An Investigator Global Assessment (IGA) score of 2-3 at Screening and Baseline.
8. Willing and able to complete once-daily eDiary entries within a consistent timeframe for the duration of the study and have ≥80% eDiary compliance rate during the washout period.
9. Judged to be in good health in the investigator's opinion.

4.1.1.2 Exclusion Criteria

Deviations from exclusion criteria are not allowed because they can potentially jeopardize the scientific integrity of the study, regulatory acceptability or participant safety. Therefore, adherence to the criteria as specified in the protocol is essential.

Participants will be excluded from the study if any of the following criteria are met:
1. Clearly defined etiology for pruritus other than atopic dermatitis. These include but are not limited to urticaria, psoriasis or other non-atopic dermatologic conditions, hepatic or renal disease, psychogenic pruritus, drug reaction, untreated hyperthyroidism, parasite presence and presence of acute infection either systemically or in the AD lesions.
2. Presence of any acute condition which may risk inducing an atopic dermatitis flare during the course of the study, such as impetigo or active herpes simplex infection.
3. Treatment with systemic corticosteroids within 4 weeks prior to randomization.
4. Treatment with Class III or higher potency topical corticosteroids or any topical anti-pruritic therapies (other than stable doses of low- or mid-potency topical corticosteroids or bland emollients) within 4 weeks prior to randomization.
5. Treatment with systemic therapies with recognized anti-pruritic (e.g. tricyclic antidepressants, sedatives, tranquilizers, gabapentin, marijuana or other cannabinoids, opioid receptor agonists/antagonists) or pruritic (e.g. opioids, angiotensin-converting enzyme inhibitors, cocaine, antimalarials) properties within 4 weeks prior to randomization.
   a. Stable doses of H1 antihistamines will be permitted. Subjects must be willing to continue these at the same doses and frequencies throughout the study inclusive of the follow-up period.
6. Any clinically significant changes in type, dose, or frequency of bland emollients, low- or mid-potency corticosteroids, and/or oral H1 antihistamines throughout the study from screening to follow-up.
7. Treatment with systemic immunosuppressive/ immunomodulatory therapies within 4 weeks prior to randomization (including but not limited to phosphodiesterase-4

inhibitors, cyclosporine, mycophenolate-mofetil, methotrexate, azathioprine, interferon-gamma, or phototherapy).
8. Treatment with biologic therapies within 12 weeks or 5 half-lives prior to randomization, whichever is longer.
9. Use of an indoor tanning facility within 4 weeks prior to randomization.
10. Treatment with any investigational therapy within 4 weeks prior to randomization.
11. Allergen immunotherapy within 6 months prior to randomization.
12. Prior use of AO+ Mist.
13. History of malignancy within 5 years prior to randomization, with the exception of completely treated and non-metastatic basal cell carcinoma or squamous cell carcinoma of the skin.
14. History of a major psychiatric condition (including major depressive disorder, bipolar disorder, or schizophrenia), suicidal ideation, or suicide attempt.
15. Known active hepatitis infection.
16. Known history of human immunodeficiency virus (HIV) infection.
17. Presence of any medical condition or disability that, in the investigator's opinion, could interfere with the assessment of safety or efficacy in this trial or compromise the safety of the subject.
18. Currently pregnant or breastfeeding, or male subject with a pregnant or breastfeeding partner.
19. Females of childbearing potential who are unable or unwilling to practice highly effective contraception (pregnancy prevention); fertile males who are unable or unwilling to use condoms with female partners of childbearing potential.

4.1.1.3 General Study Population

Subjects with pruritus and a history of atopic dermatitis will be randomized into this study.

4.2 Study Withdrawal and Withdrawal from Investigational Product and Stopping Criteria

A participant may withdraw from the study at any time at his/her own request or may be withdrawn at any time at the discretion of the Investigator.

Reasons for withdrawal (participants who refuse to complete any remaining study visits) or discontinuation (participants who prematurely stop the application) at any time during the study may include, but are not limited, to the following:

- For safety reasons, either at the discretion of the Investigator or at the participant's request
- For protocol violations at the discretion of AOBiome
- Withdrawal of consent by the Subject
- Lost to follow-up

- Due to use of concomitant therapy that could interfere with the results of the study (the Investigator will report all such information through the CRF and decide, in accordance with AOBiome, whether the participant is to be withdrawn).

The reason for participant study withdrawal will be recorded in the electronic Case Report Form (eCRF). Data from participants withdrawing from the study will be considered evaluable up to the point at which they are withdrawn using the same criteria for evaluability as for participants who complete the study.

Study stopping rules will be implemented to stop the study for safety review in the event that:

- 1 subject reports death, or
- >2 subjects report an SAE, or
- >4 subjects experience grade 3 AEs of a similar type, or
- >6 subjects experience grade 2 AEs of a similar type

when the reported SAEs or AEs are considered possibly, probably, definitely or related to the investigational product.

Atopic Dermatitis is an active disease with known seasonal and personal histories of flares and exacerbations of the patient's underlying disease state. This includes personal histories of worsening itch and itch-scratch cycles, which can lead to localized infection and localized cellulitis. A subject's known and collected clinical history should be considered by the principal investigator when determining relationship of event to the investigational product.

4.3 Early Termination

Early termination from the study may occur due to loss to follow-up or withdrawal of consent by the subject. Participants who have discontinued the study early will be evaluated by the Investigator at the Early Termination Visit. See the list of assessments to be performed at the Early Termination Visit in the Time and Events Table (Section 2.2). Participants with ongoing AEs or SAEs believed to be possibly related to investigational product (IP) will continue to be followed until resolution or for 30 days as warranted by the nature of the AE.

5 PLANNED ANALYSIS

5.1 Final Analysis

Final analysis is planned for the study after the database lock. The final analysis will follow instructions presented in this SAP. No interim reporting is planned.

6 GENERAL CONSIDERATIONS

6.1 General Summary Tables and Individual Subject Data Listings

Summary tables and listings (e.g., post text tables and individual subject data listings) are prepared according to ICH Guideline E3 and include a "footer" providing explanatory notes that indicate as a minimum the SAS program name and the date of output generation.
Post text tables also include reference(s) to the subject data listing(s) that supports the summary data and the subject data listing(s) reference the input data source(s).

Post text tables will be organized with respect to treatment group and the order of drug presentation will be the low-dose group first, followed by the high-dose group, the pooled IP treatment group, then the vehicle control. The summary tables clearly indicate the number of subjects to which the data apply and unknown or not performed are distinguished from missing data.

Summary tables for medications and medical conditions are coded according to the WHO Drug Dictionary. Adverse event preferred terms and body/organ systems are coded using the MedDRA dictionary. The MedDRA dictionary can be used, as well, in the coding of signs and symptoms, medical history, physical examination abnormalities, and clinical diagnoses.

Supportive individual subject data listings, as a minimum, are sorted and presented by treatment group and subject ID. Listings also include visit number, visit date, and days relative to the initiation of double-blind treatment.

6.2 Data Management

Biorasi will create SDTM and ADaM data sets, perform statistical analyses, and create resulting outputs using (SAS®) software version 9.4 or above.

6.3 Data Presentation Conventions

The data analysis will be conducted on all participant data when the trial ends. Subjects will be pooled across all sites. Data will be presented by treatment group and pooled B244 treatment.

All data collected will be documented using summary tables, figures, and/or patient data listings.

Continuous variables (e.g., age) are summarized using descriptive statistics (the number of subjects with available data, the mean, standard deviation (SD), median, minimum and maximum). Categorical variables (e.g., race) are summarized using counts and percentages. Percentages are calculated using the total subjects per treatment group.

The following conventions are applied to all data presentations and summaries:

- For continuous variables, all mean and median values are formatted to one more decimal place than the measured value. Standard deviation values are formatted to two more decimal places than the measured value. Minimum and maximum values are presented with the same number of decimal places as the measured value.

- For categorical variables, the number and percentage of responses are presented in the form XX (XX.X %) where the percentage is in the parentheses.

- Date variables are formatted as DDMMMYYYY for presentation. Time is formatted in military time as HH:MM for presentation.

- P-values, if applicable, will be presented to 4 decimal places. If the p-value is less than 0.0001 then it will be presented as <0.0001.

The statistical test for the primary endpoint will use a one-sided 90% CI. All other statistical tests performed will use a two-sided 95% CI.

The table and listing shells and table of contents as part of this SAP provide the expected layout and titles of the tables, listings, and figures. Any changes to format, layout, titles, numbering, or any other minor deviation will not necessitate a revision to the SAP nor will it be considered a deviation from planned analyses. Only true differences in the analysis methods or data handling will necessitate such documentation. The appropriate listings supporting the tables will be included and are not specified in the individual sections throughout the document.

6.4 Baseline Definition

The day of first dose of study medication is Day 0 in the Schedule of Assessments (SOA) per protocol but will be considered relative study Day 1 in the SAP in order to construct Clinical Data Interchange Standards Consortium (CDISC) compliant datasets.

The Baseline visit (Day 0 per protocol) will take place once the results of Screening assessments are obtained, suggesting that the subject is eligible for entering the study. During this visit, those subjects who qualify for entering the study will be randomized to one of the study arms in a ratio of 1:1:1.

For all safety analyses, Baseline will be defined as the most recent measurement prior to the first administration of study drug.

The patient reported local tolerability diary for the first 7 days of treatment will be reported as relative study Day 1 to Day 7 in the eDiary, which corresponds to Day 0 to Day 6 per protocol. For the patient reported local tolerability diary, the first measurement occurs at the end of the first day of treatment (Day 0 Baseline visit per protocol, equivalent to relative study Day 1 reported in the patient reported local tolerability diary) and solicits subjects to record any changes in local tolerability symptoms compared to the previous day prior to treatment. Relative study Day 2 to Day 7 patient reported local tolerability diary entries (Day 1 to Day 6 per protocol) solicit subjects to record any changes in local tolerability symptoms compared to the previous day of treatment.

For all efficacy analysis derived from patient reported daily diary entries (e.g., WI-NRS, AI-NRS), Baseline period is defined as the average of the 1-week period before randomization (Day

-7 to -1) where the subject is observed for their medical condition (see also Section 6.5.3). For the primary analysis, at least 1 day of daily diary entry is required to calculate the Baseline WI-NRS and AI-NRS. For the sensitivity analysis, at least 4 days of daily diary entries are required.

Baseline WI-NRS = (Sum of daily WI-NRS scores from Day -7 to Day -1) / (Total number of WI-NRS diaries entered from Day -7 to Day -1)

Baseline AI-NRS = (Sum of daily AI-NRS scores from Day -7 to Day -1) / (Total number of AI-NRS diaries entered from Day -7 to Day -1)

For efficacy analysis derived from on-site visit assessments (e.g., BSA, IGA, EASI, 5-D Pruritus Scale, POEM), Baseline is defined as the most recent measurement prior to the first administration of study drug (i.e., assessment at the Baseline visit on Day 0 per protocol or relative study Day 1).

For the rescue medication diary, the Baseline rescue medication days is the number of rescue medication days recorded during the 2 weeks (Day -14 to Day -1) prior to the Baseline visit.

6.5 Derived and Transformed Data

6.5.1 Baseline Age

Subject's age in years will be calculated based on the date of the Baseline visit date using the following formula:

Age (years) = FLOOR ((INTCK ('month', Date of Birth, Date of Baseline Visit) - (DAY(Date of Baseline Visit) < MIN(DAY(Date of Birth), DAY (INTNX ('month', Date of Baseline Visit, 1) - 1))) /12); where:

- FLOOR () is a SAS function that returns the largest integer that is less than or equal to the argument.
- INTCK () is a SAS function that returns the number of interval boundaries of a given kind that lie between two dates, times, or datetime values.
- DAY () is a SAS function that returns the day of the month from a SAS date value.
- INTNX () is a SAS function that increments a date, time, or datetime value by a given time interval, and returns a date, time, or datetime value.

6.5.2 Study Day

Day 1 is defined as the day of the Baseline visit when the subject will receive the first dose after the subject is randomized. Day 2 is defined as the day after the Baseline visit when the subject is on the second day of treatment.

- For a visit date on or after the date of the first dose:
  Study Day = (date of interest – date of first dose) + 1

- For a visit date before the date of the first dose:
  Study Day = (date of interest – date of first dose)

6.5.3 Weekly Average WI-NRS and AI-NRS

For WI-NRS and AI-NRS the weekly average is calculated from the daily scores. For the primary analysis the weekly average will be calculated when there is at least one recorded daily score in the corresponding week. Values from study day (as defined above) Day 1 to 7 contributes to Week 1 average, Study Day 8 to 14 to Week 2, etc. Baseline is derived as the average of the daily scores for Day -7 to -1. The average will be derived as the mean of all non-missing values during the respective time period.

Week 1 WI-NRS = (Sum of daily WI-NRS scores from Day 1 to Day 7) / (Total number of WI-NRS diaries entered from Day 1 to Day 7)

Week 2 WI-NRS = (Sum of daily WI-NRS scores from Day 8 to Day 14) / (Total number of WI-NRS diaries entered from Day 8 to Day 14)

Week 3 WI-NRS = (Sum of daily WI-NRS scores from Day 15 to Day 21) / (Total number of WI-NRS diaries entered from Day 15 to Day 21)

Week 4 WI-NRS = (Sum of daily WI-NRS scores from Day 22 to Day 28 or Day of Visit 4 – 1, whichever is earlier) / (Total number of WI-NRS diaries entered from Day 22 to Day 28 or Day of Visit 4 – 1, whichever is earlier)

Week 5 WI-NRS = (Sum of daily WI-NRS scores from Day of Visit 4 to Day of Visit 4 + 6) / (Total number of WI-NRS diaries entered from Day of Visit 4 to Day of Visit 4 + 6)

Week 6 WI-NRS = (Sum of daily WI-NRS scores from Day of Visit 4 + 7 to Day of Visit 4 + 13) / (Total number of WI-NRS diaries entered from Day of Visit 4 + 7 to Day of Visit 4 + 13)

Week 7 WI-NRS = (Sum of daily WI-NRS scores from Day of Visit 4 + 14 to Day of Visit 4 + 20) / (Total number of WI-NRS diaries entered from Day of Visit 4 + 14 to Day of Visit 4 + 20)

Week 8 WI-NRS = (Sum of daily WI-NRS scores from Day of Visit 4 + 21 to Day of Visit 4 + 27 or Day of Visit 5 – 1, whichever is earlier) / (Total number of WI-NRS diaries entered from Day of Visit 4 + 21 to Day of Visit 4 + 27 or Day of Visit 5 - 1, whichever is earlier)

The same conventions are used to calculate Week 1 to Week 8 AI-NRS.

Week 1 AI-NRS = (Sum of daily AI-NRS scores from Day 1 to Day 7) / (Total number of AI-NRS diaries entered from Day 1 to Day 7)

Week 2 AI-NRS = (Sum of daily AI-NRS scores from Day 8 to Day 14) / (Total number of AI-NRS diaries entered from Day 8 to Day 14)

Week 3 AI-NRS = (Sum of daily AI-NRS scores from Day 15 to Day 21) / (Total number of AI-NRS diaries entered from Day 15 to Day 21)

Week 4 AI-NRS = (Sum of daily AI-NRS scores from Day 22 to Day 28 or Day of Visit 4 – 1, whichever is earlier) / (Total number of AI-NRS diaries entered from Day 22 to Day 28 or Day of Visit 4 – 1, whichever is earlier)

Week 5 AI-NRS = (Sum of daily AI-NRS scores from Day of Visit 4 to Day of Visit 4 + 6) / (Total number of AI-NRS diaries entered from Day of Visit 4 to Day of Visit 4 + 6)

Week 6 AI-NRS = (Sum of daily AI-NRS scores from Day of Visit 4 + 7 to Day of Visit 4 + 13) / (Total number of AI-NRS diaries entered from Day of Visit 4 + 7 to Day of Visit 4 + 13)

Week 7 AI-NRS = (Sum of daily AI-NRS scores from Day of Visit 4 + 14 to Day of Visit 4 + 20) / (Total number of AI-NRS diaries entered from Day of Visit 4 + 14 to Day of Visit 4 + 20)

Week 8 AI-NRS = (Sum of daily AI-NRS scores from Day of Visit 4 + 21 to Day of Visit 4 + 27 or Day of Visit 5 – 1, whichever is earlier) / (Total number of AI-NRS diaries entered from Day of Visit 4 + 21 to Day of Visit 4 + 27 or Day of Visit 5 – 1, whichever is earlier)

Sensitivity analysis will be performed on a restricted weekly average, requiring at least 4 responses in the 7 days for each respective time period. Subjects with fewer than 4 responses during the 7-day time period(s) will have their weekly average set to missing.

6.5.4 Derivation of ≥4 Point and Any Improvement in WI-NRS and AI-NRS

The proportion of subjects with ≥4 point and any improvement (≥1 point) in WI-NRS and AI-NRS from Baseline will be derived from the weekly averages containing at least one daily score and at least 4 daily scores (as discussed in section 6.5.3 for primary and sensitivity analyses, respectively) and will be presented for each week (Weeks 1 to 8).

6.5.5 Percent Change from Baseline

Percent change from Baseline will be derived for continuous (WI-NRS, AI-NRS) endpoints and for the total score for ordinal endpoints (IGA, EASI, 5-D Pruritus Scale, POEM). Percent change from Baseline will be derived as: [(Post-Baseline value – Baseline value) / Baseline value] * 100.

For EASI, percent change from Baseline will be derived to indicate subjects who have obtained 50%, 75%, and 90% improvement.

6.5.6 Rescue Medication During Treatment Period

The treatment period (also referenced as "on-treatment") begins on date of subject's first dose and ends on Day of Visit 4 – 1, even if the subject has applied IP on Day of Visit 4.

Based on recorded use of rescue medication the following variables will be derived and normalized as described in section 7.5.4 for all subjects:

- Number of days in a month with rescue treatment (for Week 1 to Week 4 and Week 5 to Week 8)
- Number of days every 2 weeks with rescue treatment (for Day -14 to -1 for Baseline, Day 1 to Day of Visit 3 -1 for Week 2, Day of Visit 3 to Day of Visit 4 -1 for Week 4, Day of Visit 4 to Day of Visit 4 + 13 for Week 6, and Day of Visit 4 + 14 to Day of Visit 5 – 1 for Week 8)

Proportion of subjects with rescue treatment, and the above two variables (number of days in a month with rescue treatment and number of days every 2 weeks with rescue treatment) will be derived and normalized for subjects with recorded use of rescue medication.

6.5.7 Missing Start and Stop Dates for Prior and Concomitant Medication

Start date:

- If start date is completely missing, start date will be imputed with the informed consent date.
- If year is present and month and day are missing, set month and day to January 1.
- If year and day are present and month is missing, set month and day to January 1.
- If year and month are present and day is missing, set day to the 1st day of month.

Stop date:

- If end date is completely missing, end date will not be imputed and medication will be assumed to be ongoing.
- If year is present and month and day are missing, set month and day to December 31st.
- If year and day are present and month is missing, set month and day to December 31st.
- If year and month are present and day is missing, set day to the last day of month.

6.5.8 Safety Parameters

Safety and tolerability endpoints will consist of all adverse events (AEs) reported during the study duration from the date of randomization through Week 8 (End of Study Visit).
Specific AEs are defined below.

Treatment-Emergent Adverse Events (TEAE): Any AE with onset after the first dose of study medication through 28 days after the last dose of study medication.

Serious Adverse Event (SAE): An AE occurring at any dose that results in any of the following outcomes: death, a life-threatening AE, in-patient hospitalization or prolongation of existing hospitalization, a persistent or significant disability/incapacity, or a congenital anomaly/birth defect.

Associated with Use of the Study Drug: There is a reasonable possibility that the experience may have been caused by the study drug. If the Investigator does not know whether or not study drug caused the event, then the event will be handled as "related to study drug" for reporting purposes. The determination of whether an AE is related to study drug is as follows:

- Related: The AE has a missing, unknown, possible, probable or definite relationship to the study medication.
- Not related: The AE is unlikely or definitely unrelated to the study drug.

6.5.9 Missing Start and Stop Dates for Adverse Events

Start date:

- If start date is completely missing, start date is set to date of first dose.
- If (1) year is present and month and day are missing or (2) year and day are present and month is missing:
  - If year = year of first dose, then set month and day to month and day of first dose.
  - If year < year of first dose, then set month and day to December 31st.
  - If year > year of first dose, then set month and day to January 1st.
- If month and year are present and day is missing:
  - If year = year of first dose and
    - If month = month of first dose, then set day to day of first dose date.
    - If month < month of first dose, then set day to last day of month.
    - If month > month of first dose, then set day to 1st day of month.
  - If year < year of first dose, then set day to last day of month.
  - If year > year of first dose, then set day to 1st day of month.

Stop date:
If the outcome of the AE was ongoing or unknown, then the rules outlined below will not be applied.

- If stop date is completely missing, stop date is set to date of study discontinuation/end of study date.
- If (1) year is present and month and day are missing or (2) year and day are present and month is missing:
  - If year = year of study discontinuation, then set month and day to month and day of study discontinuation.
  - If year < year of study discontinuation, then set month and day to December 31st.

- If year > year of study discontinuation, then set month and day to December 31st.

- If month and year are present and day is missing:
    - If year = year of study discontinuation/end of study date and
        - If month = month of study discontinuation/end of study date, then set day to day of study discontinuation date.
        - If month < month of study discontinuation/end of study date, then set day to last day of month.
        - If month > month of study discontinuation/end of study date, then set day to 1st day of month.
    - If year < year of study discontinuation/end of study date, then set day to last day of month.
    - If year > year of study discontinuation/end of study date, then set day to 1st day of month.

6.5.10 Visit Windows

It is expected that all visits should occur according to the protocol schedule. All visit data will be tabulated per the evaluation visit as recorded on the CRF even if the assessment is outside of the visit window. All ePRO/diary data will be tabulated per the weekly conventions defined in Section 6.5.3. In data listings, the relative day of all dates will be presented.

6.5.11 Categorization of Continuous Variables

For reporting and analysis purpose continuous variables may be categorized.

Age at Baseline will be categorized in 3 groups: 18 to 40 years, 41 to 54, 55 to 65 years.

BSA will be categorized in 3 groups: 10 to 20%, >20 to 30%, >30 to 40%. If subjects are admitted into the study with baseline BSA outside of these specified ranges, then the subject will be placed into the closest occurring category. For example, if a subject is admitted into the study with a baseline BSA of 50%, then they will be included in the >30-40% category. Footnotes will be added to relevant tables, listings, or figures (TLFs) noting how many subjects out of range were included in the analysis.

6.5.12 Site Related Baseline Covariates

Site may be included as a covariate in regression models. Additional covariates reflecting site, seasonality, and geographical variation may be derived and included in sensitivity analyses.

For pooling of sites, the following covariates may be defined:

- Enrollment low to high (for example, pool by number of subjects enrolled: 1-9, 10-15, 16-25, 26+)
- Enrollment gap (based on time between enrolled patients, one or more gaps of 56 days or above between enrollment v none)

For investigation of seasonality and geography effect the following covariates on site level will be defined:

- Colder v warmer months (randomized Oct. – Mar / Apr – Sep)
- Seasonal geographic locations (warm all year v cooler all year)

For IP effect the following covariates may be defined:

- IP lot used (subjects who use at least 2 kits from the same lot can be included in the lot analysis)

Above mentioned site related baseline covariates may be derived if post-hoc analyses are performed (see Section 7.4).

7 STATISTICAL ANALYSES

7.1 Sample Size Determination

Approximately 576 subjects may be enrolled to account for 16.7% drop out rate prior to completing the study. A total of 160 evaluable subjects per group (480 total) are required to achieve at least 80% power to detect a pairwise difference of 0.65 in mean WI-NRS change from Baseline to Week 4 between one of two active doses of B244 and vehicle control when assuming a standard deviation of 2.5 and applying a Dunnett Testing Method at a one-sided familywise error rate of 0.10.

7.2 Analysis Populations

Final analysis populations will be determined and agreed upon prior to database lock and patient unblinding as described in section 7.3.2.

7.2.1 Screen Failures

Investigators must account for all subjects who sign informed consent and will maintain an Enrollment Log capturing subjects screened and indicating who was enrolled or excluded and the reason why. If the subject is found not to be eligible prior to enrollment, the reason(s) for ineligibility must be documented by the Investigator.

These subjects will neither contribute to data presentations nor be included in formal statistical analyses. The number of screen failures will be included in the data disposition table. Subject Numbers assigned to subjects who fail Screening will not be re-used.

7.2.2 Subjects Enrolled and Subjects Not Assigned

Subjects are considered enrolled if they sign informed consent and meet all eligibility criteria. Randomization is not a requirement for subjects to be considered enrolled.

Any subject who meets eligibility criteria but is not randomized (e.g., withdrew from the study, lost-to-follow-up, etc.), will be classified as "Not Assigned" in the statistical outputs. This group of subjects will not include Screen Failures as those subjects will be reported separately.

Thus, enrolled subjects consist of randomized subjects and not assigned subjects.

7.2.3 Safety Population

The safety population includes all subjects who apply at least 1 dose of study medication. Subjects will be grouped as treated.

7.2.4 Modified ITT Population

Modified Intent to Treat (mITT): All randomized participants who apply at least 1 dose of study medication and have at least one post-baseline evaluation on-site visit. Subjects will be grouped as randomized.

This is a change from the protocol.

7.2.5 Per-Protocol (PP) Population

The PP population includes all subjects in the mITT population without any major protocol deviations that may have an impact on the efficacy assessments, who complete their Week 4 visit, and who administer at least 50% of investigational product (IP). Subjects will be grouped as treated.

7.2.6 Role of Populations in Analyses

The results in the mITT population will be considered definitive for superiority of each active treatment to vehicle with those in the PP population considered supportive. Safety analyses will be performed using the Safety population.

7.3 General Analysis

7.3.1 Subjects Disposition

The subject disposition summary will include the number screened, the number of screen failures, the number enrolled, the number in each patient population for analysis, the number who completed the study, the number who discontinued the study and reason for discontinuation from the study. Disposition data will be summarized by treatment and pooled B244 treatment arm.

A summary table of analysis populations and reasons for exclusion will be presented for all subjects enrolled.

A by-subject data listing of study completion information including the reason for study discontinuation will be presented. A by-subject listing of inclusion/exclusion criteria not met will also be presented.

7.3.2 Clinical Trial Protocol Deviations

A summary of all major protocol deviations for mITT and Safety populations by category will be generated. Protocol deviation data will be summarized by treatment and pooled B244 treatment arm. A by-subject data listing of all protocol deviations will also be presented. Protocol deviations will be categorized as major or minor per Protocol Deviation Guidance v2.1.
At the discretion of the Sponsor, major protocol deviations, as determined by a review of the data prior to unblinding of the study results and the conduct of statistical analyses, may result in the removal of a subject's data from the PP population. Biorasi and its data monitoring group as applicable will be responsible for producing the final protocol deviation file, in collaboration with Sponsor; this file will include a description of the protocol deviation and clearly identify whether or not this violation warrants exclusion from the PP population. This file will be finalized prior to hard database lock.

7.3.3 Demographic and Baseline Characteristics

Demographic and Baseline characteristic data summarization will be performed in order to descriptively assess the comparability of treatment groups. Data to be tabulated will include age, race, sex, ethnicity, height, weight, BMI, smoking status, and rescue medication days during the 2-weeks prior to baseline as well as Baseline WI-NRS, AI-NRS, IGA, EASI, BSA, 5-D Pruritus Scale, and POEM scores.

7.4 Efficacy Analysis

All efficacy analyses will be performed on the mITT population and PP population using the methods described below. Regression models will include all eligible subjects for the respective analysis, from the 3 treatment arms.

- *Primary Analysis*: Includes data for all subjects in the respective population (mITT/PP)
- *Sub-Analysis*: Excludes data collected on/after date of first on-treatment rescue medication use through the end of the study.
    - For WI-NRS/AI-NRS endpoints, excludes data from *week* of first on-treatment rescue medication use.
- *Non-Responder Imputation (NRI) Analysis*: Subjects who discontinue treatment early or who take on-treatment rescue medications will be imputed as a non-responder for assessments that occur on/after date of discontinuation/on-treatment rescue medication use through the end of the study.

- For WI-NRS/AI-NRS endpoints, excludes data from *week* of discontinuation/first on-treatment rescue medication use.
- Subjects who discontinue early should have at least one treatment-emergent adverse event (TEAE) leading to discontinuation in order to have their data imputed for NRI analysis.

Efficacy Analyses

Measure	Endpoints	Analysis
WI-NRS/ AI-NRS*	Mean Change from Baseline	1. Primary<br>2. Primary/Sensitivity<br>3. Sub-Analysis<br>4. Sub-Analysis/Sensitivity
WI-NRS/ AI-NRS*	Improvement (4-Point, Any)	1. Primary<br>2. Primary/Sensitivity<br>3. Sub-Analysis<br>4. Sub-Analysis/Sensitivity<br>5. NRI<br>6. NRI/Sensitivity
5D	Mean Change from Baseline	1. Primary<br>2. Sub-Analysis
EASI	Mean Change from Baseline	1. Primary<br>2. Sub-Analysis
EASI	Improvement (≥50%, ≥75%, ≥90%)	1. Primary<br>2. Sub-Analysis<br>3. NRI
IGA	Mean Change from Baseline	1. Primary<br>2. Sub-Analysis
IGA	Improvement (2-Point to Clear/Almost Clear, Clear/Almost Clear, Any)	1. Primary<br>2. Sub-Analysis<br>3. NRI
POEM	Mean Change from Baseline	1. Primary<br>2. Sub-Analysis

*WI-NRS/AI-NRS analyses require at least 1 diary entry per week to calculate the weekly average, except for the sensitivity analyses, where at least 4 diary entries per week are required.

PP population excludes subjects with any major protocol deviation that may impact efficacy and safety analyses, including those with any confounding events or those over the rescue medication use limits but includes data for subjects below rescue medication use limits. Rescue medication

use below the allowed limit per protocol are considered minor protocol deviations, are considered non-confounding, and is included in the PP population.

The following events are confounding:

- Change in stable use of oral H1 antihistamines (e.g., change in type, dose, and frequency of medication)
- Change in stable use of bland emollients (e.g., change in type, dose, and frequency of treatment)
- Use of excluded medications during study
- Use of rescue medications over the allowed limit per protocol (over 3 rescue medication days during the 2-week washout period, over 7 rescue medication days during the 4-week treatment, and over 7 rescue medication days during the 4-week follow-up)

In the PP sub-analysis, all subject data over the rescue medication limits per the PP primary analysis will be excluded, but subject data below the rescue medication limits will be included up to the date or week that the first rescue medication is taken.

Hypothesis tests for the primary efficacy endpoint will be performed using a Dunnett Testing Method, applying pairwise comparisons of each respective B244 dose group to vehicle using a one-sided familywise error rate of 0.10. Section 7.4.2 provides further details. No additional adjustments will be made for multiple testing. As such, p-values from analysis of secondary and exploratory efficacy analyses must be interpreted in an exploratory fashion.

Supportive analyses may be performed for all efficacy analyses combining the B244 dose groups in comparison against vehicle and comparison of the B244 groups to one another.

Furthermore, stratified analyses will be performed by presenting descriptive statistics by age group, race, gender, IGA at Baseline (2 or 3), EASI at baseline (≤10 or >10), BSA range at Baseline (10 to 20%, >20 to 30%, >30 to 40%), rescue treatment used at Baseline (Yes, No), and rescue medication used while on treatment (Yes/No).

In case there are clear differences between groups in the stratified analyses, then post-hoc sensitivity analyses may be performed to control for those covariates in the appropriate statistical model. This post-hoc analysis may also include examination of outcome measures stratified by presence/absence of asthma at Baseline. Site related baseline covariates may also be analyzed post-hoc as described in Section 6.5.12. The effect of seasonality may also be investigated via seasonal pattern as one sine and one cosine function[3], if applicable, including interaction with site specific covariate. Post-hoc analysis may also include examination of outcome measures that include/exclude certain lots due to aging or stratified by investigational product lot.

7.4.1 Analysis of WI-NRS and AI-NRS

The primary endpoint of mean change from Baseline to Week 4 in WI-NRS will be analyzed using analysis of covariance (ANCOVA) models.

In addition, the continuous efficacy endpoints WI-NRS, AI-NRS will be summarized using descriptive statistics at Baseline, Week 1, Week 2, Week 3, Week 4, Week 5, Week 6, Week 7, and Week 8 for actual values and change from Baseline values.

The difference in treatment groups in change from Baseline values at post-baseline visits will be analyzed using a mixed model with repeated measures to account for within subject variability and including visit (Baseline vs. Week 1, Week 2, Week 3, Week 4, Week 5, Week 6, Week 7, Week 8), treatment group, visit-by-treatment interaction, and Baseline value as explanatory variables. The subject variability will be modelled using a compound symmetry covariance matrix. Other types of covariance structure may be applied should they provide a better model fit.

The frequency and rate of WI-NRS and AI-NRS responders at Week 1, Week 2, Week 3, Week 4, Week 5, Week 6, Week 7, Week 8 (≥4 point change from Baseline in WI-NRS and AI-NRS) and other responder endpoints (any improvement from Baseline in WI-NRS and AI-NRS, defined as ≥1 point change from Baseline) will be reported and compared between treatment groups using a logistic regression model by week. Generalized estimating equations to account for repeated measures and within-subject variability may also be applied. Responder status varies by outcome measure, endpoint, and visit. That is, a subject can be considered a responder at one visit, but not the next for a specific measure. Similarly, a subject can be considered a responder for one measure but not another at the same visit.

Additionally, daily WI-NRS and AI-NRS scores will be presented in by-subject data listings.

7.4.2 Analysis of Primary Endpoint of Change from Baseline to Week 4 in Weekly Average WI-NRS

The ANCOVA model for the primary endpoint change from Baseline to Week 4 in weekly average WI-NRS will have treatment group and Baseline weekly average WI-NRS as explanatory variables. Hypothesis testing will be done using a Dunnett Testing Method, applying pairwise comparisons of each respective B244 dose group to vehicle using a one-sided familywise error rate of 0.10. Treatment effect will be estimated as least squares means using vehicle as reference and adjusted for multiplicity according to the Dunnett Testing Method and presented with one-sided 90% CI.

Additionally, the two B244 dose groups will be compared by presenting the treatment difference estimated using least squares means.

Finally, in a separate analysis, the pooled B244 dose groups will be compared to vehicle using the same ANCOVA model.

7.4.3 Analysis of IGA, EASI, POEM, 5-D Pruritus Scale

The endpoints IGA, EASI, POEM, 5-D Pruritus Scale will be summarized using descriptive statistics and analyzed using a logistic regression model at each respective timepoints. Mean change from Baseline to Week 2, Week 4, and Week 8 in IGA, EASI, POEM, and 5-D Pruritus Scale, as well as IGA and EASI responders, will be analyzed for mITT and PP as described in section 7.4.

Responder status is determined by reaching a certain level of improvement as defined below. Responder status varies by outcome measure, endpoint, and visit. That is, a subject can be considered a responder at one visit, but not the next for a specific measure. Similarly, a subject can be considered a responder for one measure but not another at the same visit. Responder criteria for categorical measures are as follows:

- Proportion of subjects with an IGA 0 (clear) or 1 (almost clear) and ≥2-point improvement from Baseline will be reported for Week 2, Week 4, and Week 8.
- Proportion of subjects with IGA 0 (clear) or 1 (almost clear) will be reported for Week 2, Week 4, and Week 8.
- Proportion of subjects with any improvement in IGA from Baseline (defined as ≥1 point change from Baseline) will be reported for Week 2, Week 4, and Week 8.
- Proportion of subjects with at least 50%, 75%, and 90% improvement in EASI (EASI-50, EASI-75, and EASI-90, respectively) from Baseline will be reported for Week 2, Week 4, and Week 8.

Each of these outcomes will be compared between treatment groups using a logistic regression model by week. Generalized estimating equations to account for repeated measures and within-subject variability may also be applied.

7.5 Safety Analysis

Safety analyses will be conducted using the Safety population.

7.5.1 Study Drug Exposure

Subject dosing/exposure is recorded in both the ERT (patient diary entries) and EDC (site entries). In case of any discrepancies of the first and/or last exposure date between ERT and EDC, the EDC date will be used. Only ERT records that fall within the first and last dosing dates reported in the EDC (inclusive) will be retained.

Each site participating in the trial will be instructed to assess subject's compliance by weighing the investigational product at the Baseline visit, Week 2 visit, and Week 4 visit and followed every time study medication is dispensed and returned.

At the Week 2 visit, subjects will return any empty bottle (likely Bottle 1 since each bottle should last approximately 10 days and the subject should be on Bottle 2 at the time of visit) to

the site for weight measurement. At the Week 4 visit, all used and unused bottles will be returned to the site for weight measurements.

The cumulative amount of study drug exposure will be estimated by calculating the difference between the weight of all bottles of drug dispensed and the weight of all bottles returned. Differences will be summed together and reported as Net Weight of Study Drug.

The amount of product used per day will be estimated by dividing the change by the number of days the subject was on treatment. These weights will be compared to the weight of the product that would be used if the subject was compliant with the protocol. Percent compliance will be calculated as follows, with planned study drug use per day defined as 2.8g (0.14 g per pump x 10 pumps per application x 2 applications per day = 2.8 g) and number of days = 28 days:

$$\text{Percent compliance} = \frac{\text{Net Weight of Study Drug}}{[\text{Planned study drug use per day}] * \text{Numbers of days}} \times 100$$

The expected amount of study drug used over 4 weeks is approximately 0.14 g per pump x 10 pumps per application x 2 applications per day x 28 days of treatment = 78.4 g. The compliance will be calculated for Baseline to Week 4 visit.

The total dispensed and returned IP weights, the Net Weight of Study Drug, and the percent compliance during the treatment period will be summarized by treatment group and pooled B244 treatment and will be presented in a by-subject data listing. Percent compliance will be calculated for all subjects who received study drug, even if they did not return any/all of their study drug kits. Kits not returned will be assumed to be fully used and the return weight will be imputed as 0g. In the listing, the total weight dispensed, returned, net weight, and % compliance will be presented.

Dose compliance for each subject will also be calculated based on the number of daily study drug dosing diaries entered out of the total study drug dosing diaries available (2 study drug dosing diaries available per day x 28 days of treatment = 56 available entries). Only ERT dosing diary records from date of first dose through date of Visit 4-1 will be considered for calculating secondary compliance.

7.5.2 Adverse Events

Adverse events will be summarized by treatment group and pooled B244 treatment using the Medical Dictionary for Regulatory Activities (MedDRA) system organ class and preferred term. Separate tabulations will be produced for all treatment-emergent AEs (TEAEs), treatment-related TEAEs (those considered by the Investigator as at least possibly drug related), treatment-emergent SAEs (TESAEs), discontinuation from study due to TEAEs, deaths due to TEAEs, TEAEs by severity, and TEAEs occurring in ≥ 2% of any study arm. By-subject listings will be provided for all treatment-emergent and non-treatment-emergent AEs.

Any treatment emergent AEs related to local safety (e.g., erythema, edema, induration, vesiculation, etc.) will be collected by the investigator during scheduled clinic visits. In addition, solicited patient assessment of local tolerability at application site (e.g., new itching, new rash, new pain/tenderness, new burning/stinging, new skin redness/color change, etc.) and grade will be collected during the first week of treatment using eDiary to inform local skin tolerability. Investigator will determine if any new local tolerability symptom or exacerbation of an existing symptom due to treatment warrants recording as an AE.

A summary of the incidence of any adverse event, SAE, and adverse events leading to discontinuation will be presented. Summaries will display, by treatment group and pooled B244 treatment, the incidence of patients with events, the frequency of patients with events within each primary system organ class and by preferred terms. For each preferred term and each system organ class a patient will be counted only once. For summaries on severe or drug-related AE, for a given patient, the highest severity or relationship for a specific preferred term will be considered.

7.5.3 Prior and Concomitant Medications

Prior and concomitant medications will be coded using the World Health Organization (WHO) dictionary version Mar 2020 or later. Prior medication is defined as any medication with a stop date prior to the date of first dose of study drug. Concomitant medication is defined as any medication with a start date on or before the date of last dose of study drug and a stop date on or after the date of first dose of study drug.

Prior and concomitant medications will be summarized by Anatomic Therapeutic Class (ATC) Level 1 and Preferred Term by treatment group and pooled B244 treatment. Patients taking the same medication multiple times will be counted once per medication. Previous and concomitant medications will also be presented in a data listing.

7.5.4 Rescue Medications

The number of rescue medication days during the treatment period will be compared to the number of rescue medication days during the 2-week washout period (Day -14 to -1) using:

- 4-week treatment period:
    - Day 1 to Day of Visit 4 – 1 for all 4 weeks of treatment (Weeks 1 – 4)
- 2-week intervals of the treatment period:
    - Day 1 to Day of Visit 3 – 1 for first 2 weeks of treatment (Weeks 1 – 2)
    - Day of Visit 3 to Day of Visit 4 – 1 for last 2 weeks of treatment (Weeks 3 – 4)

- 4-week follow-up period:
    - Day of Visit 4 to Day of Visit 5 – 1 for all 4 weeks of follow-up (Weeks 5 – 8)
- 2-week intervals of the follow-up period:
    - Day of Visit 4 to Day of Visit 4 + 13 for first 2 weeks of follow-up (Weeks 5 – 6)

- Day of Visit 4 + 14 to Day of Visit 5 – 1 for last 2 weeks of follow-up (Weeks 7 – 8)

Each time interval will be normalized as a rate using the following formula:

$$\# \, rescue \, medication \, days = \left(\frac{\# \, days \, rescue \, medications \, used \, in \, interval}{\# \, actual \, days \, in \, interval}\right) * \# \, expected \, days \, in \, interval$$

For example, if there are 30 days in the Week 4 interval (the 2 weeks between visits 3 and 4), and a subject uses rescue medications for 7 of those 30 days, then the formula will be calculated as (7/30)*14=3.27. That is, 3.27 rescue medication days should be reported for the interval covering Weeks 3 and 4.

Change in rescue medication days from Baseline to each post-baseline visit will be tabulated. Number of rescue medication days per 2-week interval and per study period (treatment, follow-up) will be listed.

The use of any of the allowed rescue medications in the rescue medication diary data will be counted for the rescue medication days. If multiple rescue medications are reported on a single date, that date is still counted as only 1 rescue medication day. "Other" rescue medication selected in the daily diary will be confirmed to be one of the allowed rescue medications, and only the allowed rescue medications will be included as rescue medication days while others will not (such as other concomitant medications or prohibited medications).

7.5.5 Patient Reported Local Tolerability

Patient Reported Local Tolerability assesses the following local tolerability symptoms: skin redness and/or color change, itching, burning and/or stinging, pain and/or tenderness, and new or changing rash. Responses are provided according to the following scale:

0 = None
1 = Mild
2 = Moderate
3 = Severe

Each of the 5 questions for local skin tolerability will be summarized by day and treatment as categorical variable (grade) by day and as continuous variable using the score. Patient reported local tolerability will be reported from Day 1 to 7 and in shift tables. Day 1 is the first day of treatment in relation to the previous untreated day and the change in tolerability for subsequent days (Day 2 to Day 7) is in relation to the previous treated day.

7.5.6 Laboratory Data

Clinical laboratory values will be expressed in SI units reported by the central laboratory.

The actual value and change from screening will be summarized for each clinical chemistry, hematology, lipid panel, HbA1C and urinalysis parameters and by each visit. In the event of repeat values, the last non-missing value per visit will be used.

For clinical laboratory values with references ranges classifying the values as low/high/normal/abnormal, shift tables from Baseline to each post-baseline visit will be provided, where values of high and low will be classified as abnormal.

Sample for Serology was collected only at Baseline. Hence, Serology data will be only provided in a listing.

All laboratory data will be also provided in data listings. Values outside of the lab parameter's normal range will be flagged as high, low, or abnormal based on the range of the test.

7.5.7 Vital Signs and Physical Examination

Vital sign measurements will be presented for each subject in a data listing. Systolic blood pressure, diastolic blood pressure, heart rate, respiration rate, and temperature will be summarized as actual value and change from Baseline by visit.

All physical examination findings will be presented in a data listing.

7.5.8 Medical History

A by-subject data listing of medical history will be presented. Medical history will be coded using MedDRA v23.0 or higher, and the number and percentage of patients experiencing at least 1 such diagnosis by MedDRA System Organ Class (SOC) and Preferred Term (PT) will be reported.

7.5.9 COVID-19 Issues

By-subject data listings will be provided for COVID-19 test types and results, missed visits and assessments due to COVID-19 specifying reason not done, and adverse events related to COVID-19.

7.6 Handling of Dropouts or Missing Data

To account for missing daily diary entries for WI-NRS and AI-NRS, the average score for a week will be presented, where at least one score or four scores must be collected in a week for the primary and sensitivity analyses, respectively.

No other accommodations will be made for missing data other than those discussed in sections 6.5.7 and 6.5.9.

7.7 Changes to Planned Analyses

With respect to planned analyses in the protocol, the following changes have been made:

- Use of Modified Intent to Treat (mITT) population rather than Intent to Treat (ITT) population. Both populations consist of subjects who are enrolled and apply at least 1 dose of study treatment. mITT population also requires that at least one post-baseline evaluation visit occur.
- Reporting of endpoints for all visits have been added.
- Responder endpoints for EASI and IGA have been added.
- The protocol states that the treatment period is 28 days, yet the IP administration in the Schedule of Assessments covers a 29-day period (Visit 2 to Visit 4). In order to be consistent with the 28-day treatment period, the time frame of Visit 2 to Visit 4-1 will be used to report the following data:
  - NRS rating scores
  - “On-treatment” rescue medication use
  - Dosing diaries for secondary compliance

8 REFERENCES

1. Protocol Number PRB244-01, Amendment 3, Date 28-Feb-2020: “A Phase II, Randomized, Double-Blind, Vehicle Controlled Study of the Efficacy, Safety, and Tolerability of B244 Topical Spray for the Treatment of Pruritus in Adults with a History of Atopic Dermatitis.”

2. Eichenfield LF, Tom WL, Chamlin SL, et al. Guidelines of care for the management of atopic dermatitis: Section 1: Diagnosis and assessment of atopic dermatitis. J Am Acad Dermatol. 2014; 70(2):338-351.

3. Stolwijk AM, Straatman H, et al. Studying seasonality by using sine and cosine functions in regression analysis. J Epidemiol Community Health. 1999 Apr; 53(4): 235–238. doi: 10.1136/jech.53.4.235

ADDENDUM TO
STATISTICAL ANALYSIS PLAN
PHASE II

VERSION: 1.0

DATE:

April 22, 2022

BASED ON:

Statistical Analysis Plan v3.0 Date: 12-JAN-2022

Protocol Version Amendment 3 Date: 28-FEB-2020

Study Drug:

B244 Topical application

Protocol Number:

PRB244-01

Study Title:

A Phase II, Randomized, Double-Blind, Vehicle Controlled Study of the Efficacy, Safety, and Tolerability of B244 Topical Spray for the Treatment of Pruritus in Adults with a History of Atopic Dermatitis

Sponsor:

AOBiome, LLC
125 Cambridgepark Drive
Cambridge, MA 02140

ADDENDUM TO SAP REVIEW AND APPROVAL

This Addendum to Statistical Analysis Plan v3.0 (12Jan2022) has been prepared in accordance with team reviewers' specifications.

Prepared by: [redacted]

03 May 2022

[redacted] Date

Sponsor Review:

[redacted]

05 May 2022

[redacted] Date

[redacted]

05 May 2022

[redacted] Date

Table of Contents

1 Background and Purpose 4
2 Request 1: Worst Case Analysis 4
3 Request 2: Intention-to-Treat Analysis 5
4 List of Tables 6
5 Table Shells 8

1 Background and Purpose

The purpose of this addendum to the final Statistical Analysis Plan (SAP) for the PRB244-01 study (v3.0 dated 12Jan2022) is to describe additional statistical analyses that will be performed in order to address requests by [redacted] made on 27Mar2022. These requests are detailed in the sections below.

2 Request 1: Worst Case Analysis

[redacted] *You proposed the non-responder imputation analysis in which subjects who discontinue treatment early or who take on-treatment rescue medications will be imputed as a non-responder. Similar to the non-responder imputation analysis, we recommend that you provide additional analyses on the primary efficacy endpoint and relevant efficacy endpoints in which the underlying score-based variable values (e.g., WI-NRS, AI-NRS, IGA, etc.), are imputed with the worst score on/after date of first rescue medication use through the end of the study.*

As a parallel to the non-responder imputation analysis, for score-based endpoints of WI-NRS, AI-NRS, IGA, and EASI, we will perform worst-value imputation on/after the date of first on-treatment rescue medication or date of subject early discontinuation through the end of the study. For this sensitivity analysis, all values on/after the date of first on-treatment rescue medication use or all values missing due to subject early discontinuation will be replaced with the worst value observed for each subject between first post-baseline assessment and first use of on-treatment rescue medication (WOCF). In the event there is no observation after baseline recorded, WOCF will be Baseline values.

Subjects who discontinue early should have (1) at least one treatment-emergent adverse event (TEAE) leading to discontinuation and (2) discontinuation occurring prior to their Week 4 visit in order to have their data imputed for WOCF analysis.

In cases where subjects completed the treatment period, but did not complete all of the follow-up period, the following logic will be used in order to fully parallel the NRI analysis. If subjects use rescue medications while on treatment and discontinue after the Week 4 visit, then only values from first on-treatment rescue medication use through last attended visit will be imputed with WOCF (i.e., missing data from visits that did not occur after Week 4 would not be imputed in this case).

The mITT population will be used for this analysis. For WOCF imputation of WI-NRS and AI-NRS values, the worst-observation would be the highest weekly average (not highest daily score) reported after baseline and before first on-treatment rescue medication use or before subject early discontinuation.

If a situation occurs where a visit is missed but a subsequent visit takes place for the same subject, then the missing data for that interim visit will not be imputed unless that visit occurs on/after the date of first on-treatment medication use.

3 Request 2: Intention-to-Treat Analysis

[redacted] *We recommend that you perform additional sensitivity analyses to include dropouts for whom no single score is collected and thus missing data handling strategies are to be considered. In this analysis, the analysis population will be the intent-to-treat (ITT) population.*

To approximate an intent-to-treat population, LOCF methods will be applied for all randomized subjects missing Week 4 responses for endpoints (WI-NRS, AI-NRS, IGA, EASI, and responder analyses). Subjects missing Week 4 values will have their measurement imputed to be the LOCF value on treatment or baseline, whichever is later. Sensitivity analysis of WI-NRS, AI-NRS, IGA, EASI, and related responder analyses will be performed.

Intention-to-Treat Population (ITT): All subjects who were randomized, regardless of exposure to treatment.

For subjects who were randomized, but did not receive treatment, the baseline WI-NRS and AI-NRS scores will be calculated using the same approach that is used for treated subjects (as described in section 6.4 of the SAP), except that the reference date will be the date of the baseline visit instead of the date of first dose.

4 List of Tables

Display Number	Title	Population	Unique/ Repeat	Request #
14.2.2.2a	Mean Change in WI-NRS from Baseline to Week 4 – ANCOVA - WOCF	mITT	Repeat	1
14.2.2.2b	Mean Change in WI-NRS from Baseline to Week 4 – ANCOVA – WOCF Sensitivity	mITT	Repeat	1
14.2.2.2c	Mean Change in WI-NRS from Baseline to Week 4 – ANCOVA - LOCF	ITT	Repeat	2
14.2.2.3.4a	Mean Change in WI-NRS from Baseline to Weeks 1 Through 8 - WOCF	mITT	Repeat	1
14.2.2.3.4b	Mean Change in WI-NRS from Baseline to Weeks 1 Through 8 - WOCF Sensitivity	mITT	Repeat	1
14.2.2.4.4a	Mean Change in AI-NRS from Baseline to Weeks 1 Through 8 - WOCF	mITT	Repeat	1
14.2.2.4.4b	Mean Change in AI-NRS from Baseline to Weeks 1 Through 8 - WOCF Sensitivity	mITT	Repeat	1
14.2.2.4.9	Mean Change in AI-NRS from Baseline to Week 4 - LOCF	ITT	Repeat	2
14.2.2.5.13	Proportion of Subjects with ≥4 Point Improvement in WI-NRS from Baseline to Week 4 - LOCF	ITT	Repeat	2
14.2.2.7.13	Proportion of Subjects with ≥4 Point Improvement in AI-NRS from Baseline to Week 4 - LOCF	ITT	Repeat	2
14.2.2.10.2a	Mean Change in EASI Total Score from Baseline to Weeks 2, 4, and 8 - WOCF	mITT	Repeat	1
14.2.2.10.5	Mean Change in EASI Total Score from Baseline to Week 4 - LOCF	ITT	Repeat	2
14.2.2.11.2a	Mean Change in IGA from Baseline to Weeks 2, 4, and 8 - WOCF	mITT	Repeat	1

14.2.2.11.5	Mean Change in IGA from Baseline to Week 4 - LOCF	ITT	Repeat	2
14.2.2.14.7	Proportion of Subjects with ≥75% Improvement in EASI (EASI-75) from Baseline to Week 4 - LOCF	ITT	Repeat	2
14.2.2.15.7	Proportion of Subjects with ≥90% Improvement in EASI (EASI-90) from Baseline to Week 4 - LOCF	ITT	Repeat	2
14.2.2.16.7	Proportion of Subjects with IGA of Clear or Almost Clear and ≥2 Point Improvement from Baseline to Week 4 - LOCF	ITT	Repeat	2
14.2.2.17.7	Proportion of Subjects with IGA of Clear or Almost Clear at Week 4 - LOCF	ITT	Repeat	2

5 Table Shells

Table 14.2.2.2a
Mean Change in WI-NRS from Baseline to Week 4 – ANCOVA – WOCF
(mITT Population)

Visit	Statistic Type	Statistic	B244 O.D. 5.0 (N=xx)	B244 O.D. 20.0 (N=xx)	Pooled B244 (N=xx)	Vehicle (N=xx)
Week 4 CFB [1]	ANCOVA [2,3]	n	xx	xx	xx	xx
		LSM (SE) [4]	xx.x (xx.xxx)	xx x (xx xxx)	xx x (xx xxx)	xx x (xx xxx)
		95% CI	(xx x, xx x)	(xx.x, xx x)	(xx x, xx x)	(xx.x, xx x)
	v. Vehicle					
		LSM Difference (SE)	xx.x (xx.xxx)	xx x (xx xxx)	xx x (xx xxx)	N/A
		90% UCL Adjusted [5]	xx x	xx x	xx x	N/A
		p-value	x xxxx	x xxxx	x xxxx	N/A
	20.0 v 5.0					
		LSM Difference (SE)	N/A	xx x (xx xxx)	N/A	N/A
		90% UCL Adjusted	N/A	xx x	N/A	N/A
		p-value	N/A	x xxxx	N/A	N/A

Note: WOCF = Worst Observation Carried Forward.
[1] CFB = Change from Baseline.
[2] ANCOVA = Analysis of Covariance model.
[3] Model contains treatment group and baseline WI-NRS as explanatory variables, where baseline WI-NRS = (Sum of daily WI-NRS scores from Day -7 to Day -1) / (Total number of WI-NRS diaries entered from Day -7 to Day -1) and at least one WI-NRS daily score is reported between Days -7 and -1. Multiplicity is adjusted for with the Dunnett Testing Method using a one-sided familywise error rate of 0.10. All subjects who meet mITT criteria are included. Data collected from the week of first on-treatment rescue medication use through the end of the study and missing data due to subject dropout will be imputed using WOCF. Data collected prior to the week of first on-treatment rescue medication use or dropout will be included.
[4] LSM = Least Squares Mean. SE = Standard Error.
[5] UCL = Upper Confidence Limit for one-sided confidence interval.
Source: Listing XXXX
PROGRAM NAME: XXXXXXXXXX DATE: HH:MM/DDMMMYYYY

Programming Note:

- Repeat for 14.2.2.2b Mean Change in WI-NRS from Baseline to Week 4 – ANCOVA – WOCF Sensitivity for mITT Population
    - Footnote [3] will be same as in 14.2.2.2a, except changing “one WI-NRS daily score is” to “four WI-NRS daily scores are”

- Repeat for 14.2.2.2e Mean Change in WI-NRS from Baseline to Week 4 – ANCOVA – LOCF for ITT Population
  - Add new footnote "Note: LOCF = Last Observation Carried Forward." as the first footnote.
  - Footnote [3] will be "Model contains treatment group and baseline WI-NRS as explanatory variables, where baseline WI-NRS = (Sum of daily WI-NRS scores from Day -7 to Day -1) / (Total number of WI-NRS diaries entered from Day -7 to Day -1) and at least one WI-NRS daily score is reported between Days -7 and -1. Multiplicity is adjusted for with the Dunnett Testing Method using a one-sided familywise error rate of 0.10. All subjects who meet ITT criteria are included and no data is excluded based on rescue medication use. Any values missing at Week 4 will be imputed using LOCF."

Table 14.2.2.3.4a
Mean Change in WI-NRS from Baseline to Weeks 1 Through 8 – WOCF
(mITT Population)

Visit	Statistic Type	Statistic	B244 O.D. 5.0 (N=xx)	B244 O.D. 20.0 (N=xx)	Pooled B244 (N=xx)	Vehicle (N=xx)
Week 1 CFB [1]	MMRM [2,3]	n	xx	xx	xx	xx
		LSM (SE) [4]	xx.x (xx.xxx)	xx x (xx xxx)	xx x (xx xxx)	xx x (xx xxx)
		95% CI	(xx x, xx x)	(xx.x, xx x)	(xx x, xx x)	(xx.x, xx x)
	v. Vehicle	LSM Difference (SE)	xx.x (xx.xxx)	xx x (xx xxx)	xx x (xx xxx)	N/A
		95% CI	xx x	xx x	xx x	N/A
		p-value	x xxxx	x xxxx	x xxxx	N/A
	20.0 v 5.0	LSM Difference (SE)	N/A	x xxxx	N/A	N/A
		95% CI	N/A	xx x (xx xxx)	N/A	N/A
Repeat for Weeks 2 – 8		p-value	N/A	xx x	N/A	N/A

Note: WOCF = Worst Observation Carried Forward.
[1] CFB = Change from Baseline.
[2] MMRM = Mixed Model Repeated Measures.
[3] Model uses a compound symmetry covariance structure and contains visit, treatment group, visit-by-treatment interaction, and baseline WI-NRS as explanatory variables, where baseline WI-NRS = (Sum of daily WI-NRS scores from Day -7 to Day -1) / (Total number of WI-NRS diaries entered from Day -7 to Day -1) and at least one WI-NRS daily score is reported between Days -7 and -1 and for the corresponding week. All subjects who meet mITT criteria are included. Data collected from the week of first on-treatment rescue medication use through the end of the study and missing data due to subject dropout will be imputed using WOCF. Data collected prior to the week of first on-treatment rescue medication use or dropout will be included.
[4] LSM = Least Squares Mean. SE = Standard Error.
Source: Listing XXXX
PROGRAM NAME: XXXXXXXXXX DATE: HH:MM/DDMMMYYYY

Programming Note:

- Repeat for 14.2.2.3.4b Mean Change in WI-NRS from Baseline to Weeks 1 Through 8 – WOCF Sensitivity for mITT Population
    - Footnote [3] will be same as in 14.2.2.3.4a, except changing “one WI-NRS daily score is” to “four WI-NRS daily scores are”
- Repeat for 14.2.2.4.4a Mean Change in AI-NRS from Baseline to Weeks 1 Through 8 – WOCF for mITT Population
    - Footnote [3] will be same as in 14.2.2.3.4a, except changing “WI-NRS” to “AI-NRS”
- Repeat for 14.2.2.4.4b Mean Change in AI-NRS from Baseline to Weeks 1 Through 8 – WOCF Sensitivity for mITT Population

  - Footnote [3] will be same as in 14.2.2.3.4b, except changing “WI-NRS” to “AI-NRS”
- Repeat for 14.2.2.4.9 Mean Change in AI-NRS from Baseline to Week 4 – LOCF for ITT Population
  - First column will contain only “Week 4 CFB [1]”
  - Add new footnote “Note: LOCF = Last Observation Carried Forward.” as the first footnote.
  - Footnote [3] will be “Model uses a compound symmetry covariance structure and contains treatment group and baseline AI-NRS as explanatory variables, where baseline AI-NRS = (Sum of daily AI-NRS scores from Day -7 to Day -1) / (Total number of AI-NRS diaries entered from Day -7 to Day -1) and at least one AI-NRS daily score is reported between Days -7 and -1 and for the corresponding week. All subjects who meet ITT criteria are included and no data is excluded based on rescue medication use. Any values missing at Week 4 will be imputed using LOCF.”

Table 14.2.2.5.13
Proportion of Subjects with ≥4 Point Improvement in WI-NRS from Baseline to Week 4 – LOCF
(ITT Population)

Visit	Statistic Type	Statistic	B244 O.D. 5.0 (N=xx)	B244 O.D. 20.0 (N=xx)	Pooled B244 (N=xx)	Vehicle (N=xx)
Week 4	Logistic [1,2]	n/N (Proportion %) [3]<br>95% CI	xx/xx (xx x)<br>(xx x, xx.x)	xx/xx (xx x)<br>(xx x, xx x)	xx/xx (xx x)<br>(xx x, xx.x)	xx/xx (xx.x)<br>(xx x, xx x)
	v. Vehicle	Odds Ratio (95% CI)<br>p-value	x xx (x.xx, x xx)<br>x.xxxx	x xx (x xx, x.xx)<br>x xxxx	x xx (x xx, x xx)<br>x.xxxx	N/A<br>N/A
	20.0 v 5.0	Odds Ratio (95% CI)<br>p-value	N/A<br>N/A	x xx (x xx, x.xx)<br>x xxxx	N/A<br>N/A	N/A<br>N/A

Note: LOCF = Last Observation Carried Forward.
[1] Logistic regression model contains treatment group as the explanatory variable. At least one WI-NRS daily score is reported between Days -7 and -1 and for the corresponding week. All subjects who meet ITT criteria are included and no data is excluded based on rescue medication use. Any values missing at Week 4 will be imputed using LOCF.
[2] Treatment response is improvement by ≥ 4 Points in WI-NRS score (i.e., decrease in WI-NRS score by at least 4 points from Baseline to Post-Baseline visit).
[3] n is the number of subjects who have improved at that visit. N is the number of subjects with data at that visit, regardless of improvement status.
Source: Listing XXXX
PROGRAM NAME: XXXXXXXXXX DATE: HH:MM/DDMMMYYYY

Programming Note:

- Repeat for 14.2.2.7.13 Proportion of Subjects with ≥4 Point Improvement in AI-NRS from Baseline to Week 4 – LOCF for mITT Population
  - Add new footnote "Note: LOCF = Last Observation Carried Forward." as the first footnote.
  - Footnote [1] will be same as in 14.2.2.5.13, except changing "WI-NRS" to "AI-NRS"

Table 14.2.2.10.2a
Mean Change in EASI Total Score from Baseline to Weeks 2, 4, and 8 – WOCF
(mITT Population)

Visit	Statistic Type	Statistic	B244 O.D. 5.0 (N=xx)	B244 O.D. 20.0 (N=xx)	Pooled B244 (N=xx)	Vehicle (N=xx)
Week 2 CFB [1]	MMRM [2,3]	n	xx	xx	xx	xx
		LSM (SE) [4]	xx.x (xx.xxx)	xx x (xx xxx)	xx x (xx xxx)	xx x (xx xxx)
		95% CI	(xx x, xx x)	(xx.x, xx x)	(xx x, xx x)	(xx.x, xx x)
	v. Vehicle	LSM Difference (SE)	xx.x (xx.xxx)	xx x (xx xxx)	xx x (xx xxx)	N/A
		95% CI	(xx x, xx x)	(xx.x, xx x)	(xx x, xx x)	N/A
		p-value	x xxxx	x xxxx	x xxxx	N/A
	20.0 v 5.0	LSM Difference (SE)	N/A	xx x (xx xxx)	N/A	N/A
		95% CI	N/A	(xx.x, xx x)	N/A	N/A
Repeat for Weeks 4 and 8		p-value	N/A	x xxxx	N/A	N/A

Note: WOCF = Worst Observation Carried Forward.
[1] CFB = Change from Baseline.
[2] MMRM = Mixed Model with Repeated Measures.
[3] Model uses a compound symmetry covariance structure and contains visit, treatment group, visit-by-treatment interaction, and baseline EASI total score as explanatory variables. All subjects who meet mITT criteria are included. Data collected from the week of first on-treatment rescue medication use through the end of the study and missing data due to subject dropout will be imputed using WOCF. Data collected prior to the week of first on-treatment rescue medication use or dropout will be included.
[4] LSM = Least Squares Mean. SE = Standard Error.
Source: Listing XXXX
PROGRAM NAME: XXXXXXXXXX DATE: HH:MM/DDMMMYYYY

Programming Note:

- Repeat for 14.2.2.10.5 Mean Change in EASI Total Score from Baseline to Week 4 – LOCF for ITT Population
    - First column will contain only “Week 4 CFB [1]”
    - Add new footnote “Note: LOCF = Last Observation Carried Forward.” as the first footnote.
    - Footnote [3] will be “Model uses a compound symmetry covariance structure and contains treatment group and baseline EASI total score as explanatory variables. All subjects who meet ITT criteria are included and no data is excluded based on rescue medication use. Any values missing at Week 4 will be imputed using LOCF.”
- Repeat for 14.2.2.11.2a Mean Change in IGA from Baseline to Weeks 2, 4, and 8 – WOCF for mITT Population
    - Footnote [3] will be same as in 14.2.2.10.2a, except changing “EASI” to “IGA”

- Repeat for 14.2.2.10.5 Mean Change in IGA from Baseline to Week 4 – LOCF for ITT Population
    - First column will contain only “Week 4 CFB [1]”
    - Add new footnote “Note: LOCF = Last Observation Carried Forward.” as the first footnote.
    - Footnote [3] will be same as in 14.2.2.10.5, except changing “EASI” to “IGA”

Table 14.2.2.14.7
Proportion of Subjects with ≥75% Improvement in EASI (EASI-75) from Baseline to Week 4 – LOCF
(ITT Population)

Visit	Statistic Type	Statistic	B244 O.D. 5.0 (N=xx)	B244 O.D. 20.0 (N=xx)	Pooled B244 (N=xx)	Vehicle (N=xx)
Week 4	Logistic [1,2]	n/N (Proportion %) [3]<br>95% CI	xx/xx (xx x)<br>(xx x, xx.x)	xx/xx (xx x)<br>(xx x, xx x)	xx/xx (xx x)<br>(xx x, xx.x)	xx/xx (xx.x)<br>(xx x, xx x)
	v. Vehicle	Odds Ratio (95% CI)<br>p-value	x xx (x.xx, x xx)<br>x.xxxx	x xx (x xx, x.xx)<br>x xxxx	x xx (x xx, x xx)<br>x.xxxx	N/A<br>N/A
	20.0 v 5.0	Odds Ratio (95% CI)<br>p-value	N/A<br>N/A	x xx (x xx, x.xx)<br>x xxxx	N/A<br>N/A	N/A<br>N/A

Note: LOCF = Last Observation Carried Forward.
[1] Logistic regression model contains treatment group as the explanatory variable. All subjects who meet ITT criteria are included and no data is excluded based on rescue medication use. Any values missing at Week 4 will be imputed using LOCF.
[2] Treatment response is improvement by ≥ 75% in EASI score.
[3] n is the number of subjects who have improved at that visit. N is the number of subjects with data at that visit, regardless of improvement status.
Source: Listing XXXX
PROGRAM NAME: XXXXXXXXXX DATE: HH:MM/DDMMMYYYY

Programming Note:

- Repeat for 14.2.2.15.7 Proportion of Subjects with ≥90% Improvement in EASI (EASI-90) from Baseline to Week 4 – LOCF for ITT Population
    - Footnote [2] will be same as in 14.2.2.14.7, except changing “75%” to “90%”
- Repeat for 14.2.2.16.7 Proportion of Subjects with IGA of Clear or Almost Clear and ≥2-Point Improvement from Baseline to Week 4 – LOCF for ITT Population
    - Footnote [2] will be “Treatment response is improvement by ≥ 2 Points in IGA score to Clear or Almost Clear.“
- Repeat for 14.2.2.17.7 Proportion of Subjects with IGA of Clear or Almost Clear at Week 4 – LOCF for ITT Population
    - Footnote [2] will be “Treatment response is IGA score of 0 (Clear) or 1 (Almost Clear).“

DocuSign

Certificate Of Completion

Enve ope Id: [redacted]
Subject: P ease DocuS gn: PRB244-01 ADDENDUM TO SAP V1.0 22Apr2022 c ean.docx
Sponsor Project Code: 128-3
Qua ty Document Type: Not App cab e
Source Enve ope:
Document Pages: 15
Cert f cate Pages: 5
AutoNav: Enab ed
Enve opeId Stamp ng: D sab ed
T me Zone: (UTC-08:00) Pac f c T me (US & Canada)

S gnatures: 3
In t a s: 0

Status: Comp eted

Enve ope Or g nator:
[redacted]

Record Tracking

Status: Or g na
5/3/2022 4:41:14 AM

Ho der: [redacted]

Locat on: DocuS gn

Signer Events	Signature	Timestamp
[redacted]<br>Secur ty Leve : Ema , Account Authent cat on (Requ red)	[redacted]<br>S gnature Adopt on: Pre-se ected Sty e<br>S gned by nk sent to [redacted]<br>S gnature ID:<br>[redacted]<br>W th S gn ng Authent cat on v a DocuS gn password<br>W th S gn ng Reasons (on each tab):<br>I am the author of th s document	Sent: 5/3/2022 4:44:13 AM<br>V ewed: 5/3/2022 4:44:27 AM<br>S gned: 5/3/2022 4:44:42 AM
**Electronic Record and Signature Disclosure:**<br>Accepted: 6/24/2020 9:03:50 AM<br>[redacted]		
[redacted]<br>Secur ty Leve : Ema , Account Authent cat on (Requ red)	[redacted]<br>S gnature Adopt on: Pre-se ected Sty e<br>S gned by nk sent to [redacted]<br>S gnature ID:<br>[redacted]<br>W th S gn ng Authent cat on v a DocuS gn password<br>W th S gn ng Reasons (on each tab):<br>I approve th s document	Sent: 5/3/2022 4:44:44 AM<br>V ewed: 5/5/2022 5:49:42 AM<br>S gned: 5/5/2022 5:52:00 AM
**Electronic Record and Signature Disclosure:**<br>Accepted: 5/5/2022 5:49:42 AM<br>ID: 39f870e3-9d2b-49b2-bc22-03f6bfe363e4		

Signer Events	Signature	Timestamp
[redacted]<br>[redacted], Account Authent cat on (Requ red)	[redacted]<br><br>S gnature Adopt on: Pre-se ected Sty e<br>S gned by nk sent to [redacted]<br>S gnature ID:<br>[redacted]<br><br>W th S gn ng Authent cat on v a DocuS gn password<br>W th S gn ng Reasons (on each tab):<br>I approve th s document	Sent: 5/5/2022 5:52:02 AM<br>V ewed: 5/5/2022 6:06:23 AM<br>S gned: 5/5/2022 6:06:57 AM
**Electronic Record and Signature Disclosure:**<br>Accepted: 3/31/2020 2:15:49 PM<br>[redacted]		

[redacted]	[redacted]	Timestamp

Editor Delivery Events	Status	Timestamp

Agent Delivery Events	Status	Timestamp

Intermediary Delivery Events	Status	Timestamp

Certified Delivery Events	Status	Timestamp

Carbon Copy Events	Status	Timestamp

Witness Events	Signature	Timestamp

Notary Events	Signature	Timestamp

Envelope Summary Events	Status	Timestamps
Enve ope Sent	Hashed/Encrypted	5/3/2022 4:44:13 AM
Cert f ed De vered	Secur ty Checked	5/5/2022 6:06:23 AM
S gn ng Comp ete	Secur ty Checked	5/5/2022 6:06:57 AM
Comp eted	Secur ty Checked	5/5/2022 6:06:57 AM

Payment Events	Status	Timestamps

Electronic Record and Signature Disclosure

ELECTRONIC RECORD AND SIGNATURE DISCLOSURE

From time to time, Biorasi, LLC (we, us or Company) may be required by law to provide to you certain written notices or disclosures. Described below are the terms and conditions for providing to you such notices and disclosures electronically through the DocuSign system. Please read the information below carefully and thoroughly, and if you can access this information electronically to your satisfaction and agree to this Electronic Record and Signature Disclosure (ERSD), please confirm your agreement by selecting the check-box next to 'I agree to use electronic records and signatures' before clicking 'CONTINUE' within the DocuSign system.

Getting paper copies

At any time, you may request from us a paper copy of any record provided or made available electronically to you by us. You will have the ability to download and print documents we send to you through the DocuSign system during and immediately after the signing session and, if you elect to create a DocuSign account, you may access the documents for a limited period of time (usually 30 days) after such documents are first sent to you. After such time, if you wish for us to send you paper copies of any such documents from our office to you, you will be charged a $0.00 per-page fee. You may request delivery of such paper copies from us by following the procedure described below.

Withdrawing your consent

If you decide to receive notices and disclosures from us electronically, you may at any time change your mind and tell us that thereafter you want to receive required notices and disclosures only in paper format. How you must inform us of your decision to receive future notices and disclosure in paper format and withdraw your consent to receive notices and disclosures electronically is described below.

Consequences of changing your mind

If you elect to receive required notices and disclosures only in paper format, it will slow the speed at which we can complete certain steps in transactions with you and delivering services to you because we will need first to send the required notices or disclosures to you in paper format, and then wait until we receive back from you your acknowledgment of your receipt of such paper notices or disclosures. Further, you will no longer be able to use the DocuSign system to receive required notices and consents electronically from us or to sign electronically documents from us.

All notices and disclosures will be sent to you electronically

Unless you tell us otherwise in accordance with the procedures described herein, we will provide electronically to you through the DocuSign system all required notices, disclosures, authorizations, acknowledgements, and other documents that are required to be provided or made available to you during the course of our relationship with you. To reduce the chance of you inadvertently not receiving any notice or disclosure, we prefer to provide all of the required notices and disclosures to you by the same method and to the same address that you have given us. Thus, you can receive all the disclosures and notices electronically or in paper format through the paper mail delivery system. If you do not agree with this process, please let us know as described below. Please also see the paragraph immediately above that describes the consequences of your electing not to receive delivery of the notices and disclosures electronically from us.

How to contact Biorasi, LLC:

You may contact us to let us know of your changes as to how we may contact you electronically, to request paper copies of certain information from us, and to withdraw your prior consent to receive notices and disclosures electronically as follows:
To contact us by email send messages to: [redacted]

To advise Biorasi, LLC of your new email address

To let us know of a change in your email address where we should send notices and disclosures electronically to you, you must send an email message to us at [redacted] and in the body of such request you must state: your previous email address, your new email address. We do not require any other information from you to change your email address.

If you created a DocuSign account, you may update it with your new email address through your account preferences.

To request paper copies from Biorasi, LLC

To request delivery from us of paper copies of the notices and disclosures previously provided by us to you electronically, you must send us an email to mrudolph@biorasi.com and in the body of such request you must state your email address, full name, mailing address, and telephone number. We will bill you for any fees at that time, if any.

To withdraw your consent with Biorasi, LLC

To inform us that you no longer wish to receive future notices and disclosures in electronic format you may:

i. decline to sign a document from within your signing session, and on the subsequent page, select the check-box indicating you wish to withdraw your consent, or you may;

ii. send us an email to [redacted] and in the body of such request you must state your email, full name, mailing address, and telephone number. We do not need any other information from you to withdraw consent.. The consequences of your withdrawing consent for online documents will be that transactions may take a longer time to process..

Required hardware and software

The minimum system requirements for using the DocuSign system may change over time. The current system requirements are found here: https://support.docusign.com/guides/signer-guide-signing-system-requirements.

Acknowledging your access and consent to receive and sign documents electronically

To confirm to us that you can access this information electronically, which will be similar to other electronic notices and disclosures that we will provide to you, please confirm that you have read this ERSD, and (i) that you are able to print on paper or electronically save this ERSD for your future reference and access; or (ii) that you are able to email this ERSD to an email address where you will be able to print on paper or save it for your future reference and access. Further, if you consent to receiving notices and disclosures exclusively in electronic format as described herein, then select the check-box next to 'I agree to use electronic records and signatures' before clicking 'CONTINUE' within the DocuSign system.

By selecting the check-box next to 'I agree to use electronic records and signatures', you confirm that:

- You can access and read this Electronic Record and Signature Disclosure; and
- You can print on paper this Electronic Record and Signature Disclosure, or save or send this Electronic Record and Disclosure to a location where you can print it, for future reference and access; and
- Until or unless you notify Biorasi, LLC as described above, you consent to receive exclusively through electronic means all notices, disclosures, authorizations, acknowledgements, and other documents that are required to be provided or made available to you by Biorasi, LLC during the course of your relationship with Biorasi, LLC.

Appendix (Supplemental)

Table 1 Supplemental
Inclusion and exclusion criteria:

Inclusion Criteria:	1. Male and female subjects 18 to 65 years of age.<br>2. Pruritus of at least 4 weeks duration prior to the initial Screening visit and during the 2 week washout period.<br>a. Subjects using stable doses of oral H1 antihistamines at the initial Screening visit must be willing to continue these at the same doses and frequencies throughout the study inclusive of the follow-up period.<br>3. Worst Itch Numeric Rating Scale (WI-NRS) score ≥ 7 in the 24-hour period prior to the initial Screening as well as Baseline visits.<br>4. Average weekly WI-NRS score ≥6 for each week of the washout period, as recorded in the eDiary.<br>5. A history of atopic dermatitis for greater than 12 months consistent with a diagnosis of atopic dermatitis, as defined by the 2014 American Academy of Dermatology (AAD) Guidelines of Care for the Management of Atopic Dermatitis.[5]<br>a. Subjects using bland emollients at the initial Screening visit will be allowed to continue to use their emollient of choice at the same dose and frequency throughout the study.<br>b. Subjects using low- to mid-potency topical corticosteroids at the initial Screening visit will be allowed to use their topical corticosteroid of choice at the same dose and frequency no more than 7 days per month throughout the study as rescue medication.<br>6. A minimum of 10% and not more than 40% of the subjects' Body Surface Area (BSA) affected by atopic dermatitis (AD) (*affected* is defined by physical examination findings: erythema, edema, scaling, lichenification, excoriation, with the excoriation serving as the physical examination correlate of pruritus) at Screening and Baseline.<br>a. Subjects' BSA can include face and body OR body alone BUT NOT face alone.<br>7. An Investigator Global Assessment (IGA) score of 2-3 at Screening and Baseline.<br>8. Willing and able to complete once-daily eDiary entries within a consistent timeframe for the duration of the study and have ≥80% eDiary compliance rate during the washout period.<br>9. Judged to be in good health in the investigator's opinion.

Exclusion Criteria:	1. Clearly defined etiology for pruritus other than atopic dermatitis. These include but are not limited to urticaria, psoriasis or other non-atopic dermatologic conditions, hepatic or renal disease, psychogenic pruritus, drug reaction, untreated hyperthyroidism, parasite presence and presence of acute infection either systemically or in the AD lesions.<br>2. Presence of any acute condition which may risk inducing an atopic dermatitis flare during the course of the study, such as impetigo or active herpes simplex infection.<br>3. Treatment with systemic corticosteroids within 4 weeks prior to randomisation.<br>4. Treatment with Class III or higher potency topical corticosteroids or any topical anti-pruritic therapies (other than stable doses of low- or mid-potency topical corticosteroids or bland emollients) within 4 weeks prior to randomisation.<br>5. Treatment with systemic therapies with recognized anti-pruritic (e.g. tricyclic antidepressants, sedatives, tranquilizers, gabapentin, marijuana or other cannabinoids, opioid receptor agonists/antagonists) or pruritic (e.g. opioids, angiotensin-converting enzyme inhibitors, cocaine, antimalarials) properties within 4 weeks prior to randomisation.<br>a. Stable doses of H1 antihistamines will be permitted. Subjects must be willing to continue these at the same doses and frequencies throughout the study inclusive of the follow-up period.<br>6. Any clinically significant changes in type, dose, or frequency of bland emollients, low- or mid-potency corticosteroids, and/or oral H1 antihistamines throughout the study from screening to follow-up.<br>7. Treatment with systemic immunosuppressive/ immunomodulatory therapies within 4 weeks prior to randomisation (including but not limited to phosphodiesterase-4 inhibitors, cyclosporine, mycophenolate-mofetil, methotrexate, azathioprine, interferon-gamma, or phototherapy).<br>8. Treatment with biologic therapies within 12 weeks or 5 half-lives prior to randomisation, whichever is longer.<br>9. Use of an indoor tanning facility within 4 weeks prior to randomisation.<br>10. Treatment with any investigational therapy within 4 weeks prior to randomisation.<br>11. Allergen immunotherapy within 6 months prior to randomisation.<br>12. Prior use of AO+ Mist.<br>13. History of malignancy within 5 years prior to randomisation, with the exception of completely treated and non-metastatic basal cell carcinoma or squamous cell carcinoma of the skin.<br>14. History of a major psychiatric condition (including major depressive disorder, bipolar disorder, or schizophrenia), suicidal ideation, or suicide attempt.<br>15. Known active hepatitis infection.<br>16. Known history of human immunodeficiency virus (HIV) infection.<br>17. Presence of any medical condition or disability that, in the investigator's opinion, could interfere with the assessment of safety or efficacy in this trial or compromise the safety of the subject.

	18. Currently pregnant or breastfeeding, or male subject with a pregnant or breastfeeding partner.<br>19. Females of childbearing potential who are unable or unwilling to practice highly effective contraception (pregnancy prevention); fertile males who are unable or unwilling to use condoms with female partners of childbearing potential.

File 1 Supplemental
Excluded medications:

Within 4 weeks prior to Baseline through the Follow-up period:

- Systemic immunosuppressive/immunomodulating drugs (i.e., methotrexate, cyclosporine, etc.).
- Immunoglobulin or blood products.
- Systemic corticosteroids (oral, IV, injectable)
- NK1-R antagonists
- Class III or higher potency topical corticosteroids or any topical anti-pruritic therapies (other than stable doses of low- or mid-potency topical corticosteroids or bland emollients)
- Systemic therapies with recognized anti-pruritic (e.g. tricyclic antidepressants, sedatives, tranquilizers, gabapentin, marijuana or other cannabinoids, opioid receptor agonists/antagonists) or pruritic (e.g. opioids, angiotensin-converting enzyme inhibitors, cocaine, antimalarials) properties
    - Stable doses of oral H1 antihistamines will be permitted
- Systemic immunosuppressive/immunomodulatory therapies (including but not limited to PDE4 inhibitors, cyclosporine, mycophenolate-mofetil, methotrexate, azathioprine, interferon-gamma, or phototherapy)
- Allergen immunotherapy
- Any investigational therapy
- Strong CYP3A4 inhibitors, such as
    - boceprevir
    - clarithromycin
    - cobicistat
    - conivaptan
    - danoprevir and ritonavir
    - diltiazem
    - elvitegravir and ritonavir
    - regular grapefruit juice consumption
    - idelalisib
    - indinavir and ritonavir
    - itraconazole
    - ketoconazole
    - lopinavir and ritonavir
    - nefazodone
    - nelfinavir
    - paritaprevir and ritonavir and (ombitasvir and/or dasabuvir)
    - posaconazole
    - ritonavir
    - saquinavir and ritonavir
    - telaprevir
    - tipranavir and ritonavir

- troleandomycin
- voriconazole

Within 2 weeks prior to Baseline through the Follow-up period:

- High-potency topical corticosteroids (Class 1-3). The use of low- to mid-potency topical corticosteroids (Class 4-7), inhaled corticosteroids, or intranasal corticosteroids will be allowed.
- Use of crisaborole ointment.
- Systemic antibiotics.
- Bleach baths or topical coal tar.
- Topical calcineurin inhibitor use (e.g., pimecrolimus, tacrolimus).
- New onset use of systemic antihistamines.
- UVA or UVB phototherapy
- Topical and oral antibiotics/antiviral/antifungal/antiseptic agents
- Topical probiotics
- Topical antihistamines.
- The use of intranasal and oral antihistamines will be allowed[a].

a Subjects following stable regimens (≥2 weeks consistent for intranasal and 3 months for oral use before study baseline) with systemic antihistamines were permitted to continue use but were instructed not to alter the dose or stop the regimen while in the study within 1 week prior to Baseline.

Biologics:

- Cell-depleting agents, including but not limited to rituximab: within 6 months of baseline.
- Infliximab, adalimumab, golimumab, certolizumab pegol, abatacept, etanercept, anakinra, and dupilumab: within 12 weeks of baseline, or 5 half-lives, whichever is longer.
- Other biologics: within 12 weeks of Baseline, or 5 half-lives (if known), whichever is longer.

Permitted rescue medications:

Over the course of the study, the Investigator will monitor and evaluate the subject's condition and determine whether rescue therapy may be necessary. The use of medications to treat a subject's atopic dermatitis during the study will be permitted if a medical professional determines that it becomes medically necessary. Such rescue treatment should not exceed 7 days per month for the duration of the trial (screening, washout, treatment, and follow-up), e.g., no more than 3 days total during the 2 week washout period, no more than 7 days total during the 4 week treatment period, and no more than 7 days total during the 4 week follow-up period. If a subject requires treatment for more than 7 days per month or requires systemic therapy, they may be discontinued from the study.

**Class 4 – Mid-strength**	
Clocortolone pivalate (0·1%)	Cloderm® Cream
Mometasone furoate (0·1%)	Elocon® Cream
Triamcinolone acetonide (0·1%)	Aristocort® A Cream, Kenalog® Ointment
Betamethasone valerate (0·1%)	Valisone Ointment
Fluocinolone acetonide (0·025%)	Synalar® Ointment
**Class 5 – Lower Mid-strength**	
Fluticasone propionate (0·05%)	Cutivate® Cream/Cutivate Lotion
Prednicarbate (0·1%)	Dermatop® Cream
Hydrocortisone probutate (0·1%)	Pandel® Cream
Triamcinolone acetonide (0·1%)	Aristocort A Cream, Kenalog Lotion
Fluocinolone acetonide (0·025%)	Synalar Cream
**Class 6 – Mild**	
Alclometasone dipropionate (0·05%)	Aclovate® Cream/Ointment
Desonide (0·05%)	Verdeso™ Foam, Desonate Gel™
Triamcinolone acetonide (0·025%)	Aristocort A Cream, Kenalog Lotion

Hydrocortisone butyrate (0·1%)	Locoid Cream/Ointment
Fluocinolone acetonide (0·01%)	Derma-Smoothe/FS® Scalp Oil, Synalar Topical Solution
**Class 7 – Least potent**	
Hydrocortisone (2%/2·5%)	Nutracort® Lotion, Synacort Cream
Hydrocortisone (0·5 to 0·1%)	Cortaid® Cream/Spray/Ointment and many other over-the-counter products

File 2 Supplemental.

Patient Reported Local Tolerability:

INSTRUCTIONS:

Please examine all of your skin lesions that are treated with B244 topical solution. Please circle one score for each category that best describes the changes you observed in all skin lesions. For each day of the first 7 days of treatment, please examine all skin lesions at approximately the same time every day.

**SKIN REDNESS AND/OR COLOR CHANGE AT APPLICATION SITE**		
Score	Grade	Definition
0	None	No new redness or new color change; no new increase in redness or new increase in color change
1	Mild	Slight increase in redness or slight increase in color change
2	Moderate	Increase in redness or increase in color change
3	Severe	Intense redness or intense color change

ITCHING AT APPLICATION SITE		
Score	Grade	Definition
0	None	No new itching or new scratching
1	Mild	Slight increase in itching or slight increase in scratching
2	Moderate	Increase in itching or increase in scratching that is not disturbing sleep
3	Severe	Increase in itching or increase in scratching that is disturbing sleep

BURNING AND/OR STINGING AT APPLICATION SITE		
Score	Grade	Definition
0	None	No new burning or new stinging
1	Mild	Slight increase in warm or tingling sensation that is not bothersome
2	Moderate	Increase in warm or tingling sensation that is bothersome
3	Severe	Hot or stinging sensation that is causing definite discomfort

PAIN AND/OR TENDERNESS AT APPLICATION SITE		
Score	Grade	Definition
0	None	No new pain or new tenderness
1	Mild	Slight increase in pain or slight increase in tenderness that is not bothersome
2	Moderate	Increase in pain or increase in tenderness that is bothersome
3	Severe	Intense pain or intense tenderness causing definite discomfort or disturbing sleep

NEW OR CHANGING RASH AT APPLICATION SITE		
Score	Grade	Definition
0	None	No new rash or no change in rash (such as swelling, fullness, or blistering)
1	Mild	Slight new rash or slight increase in rash (such as swelling, fullness, or blistering) that is not bothersome
2	Moderate	New rash or increase in rash (such as swelling, fullness, or blistering) that is bothersome
3	Severe	Significant new rash or significant increase in rash (swelling, fullness, or blistering) causing discomfort

Supplemental Results:

A total of 28% (46/166) of the subjects in the Optical density (OD) 5·0 treatment group and a total of 38% (64/164) in the OD 20·0 treatment group had an improvement to Clear or Almost Clear in Investigator global assessment (IGA) from baseline to Week 4 in the modified intent to treat (mITT) population and was similar in the per protocol (PP) population. Collectively, 33% (108/330) of subjects in the pooled B244 groups experienced improvement to Clear or Almost clear at Week 4, whereas only 20% (34/171) of subjects in the vehicle group experienced this same level of improvement. Although the OD 5·0 group did not have a significantly greater proportion of subjects with improvement to Clear or Almost Clear compared to the vehicle group (OR=1·54, p=0·093), the OD 20·0 and pooled B244 groups did reach the level of statistical significance (OR=2·45, p=0·0003 and OR=1·96, p=0·0027, respectively) compared to the vehicle group. Although no difference was observed comparing OD 5·0 to OD 20·0, it did start to approach statistical significance (OR=1·59, p=0·051). Results for the sub-analysis and non responder imputation (NRI) analysis were consistent with that of the primary analysis for proportion of subjects with IGA of Clear or Almost Clear at Week 4 (Table 2 Supplemental).

A total of 15% (25/166) of the subjects in the OD 5·0 treatment group and a total of 18% (30/164) in the OD 20·0 treatment group had a ≥ 90% improvement in the eczema area and severity index (EASI) score from baseline to Week 4 in the mITT population and was similar in the PP population. Collectively, 17% (55/330) of subjects in the B244 groups experienced at least a 90% improvement in EASI total score. Whereas only 6% (10/171) of subjects in the vehicle group experienced this level of improvement. All B244 groups had a significantly higher proportion of subjects who experienced this response rate compared to subjects in the vehicle group (OR=2·85, p=0·0074 for OD 5·0; OR=3·60, p-value=0·0008 for OD 20·0; and OR=3·22, p=0·0011 for pooled B244). Similar to other analyses, no difference was observed between OD 5·0 and OD 20·0 at Week 4 (OR=1·26, p=0·43). The results for the sub-analysis and NRI analysis were consistent with the proportion of subjects with ≥90% improvement in EASI total score from baseline to Week 4 (Table 2 Supplemental).

Improvements in mean change in the patient oriented eczema measure (POEM) score from baseline to Week 4 for OD 5·0, OD 20·0, pooled group, and the vehicle group and were respectively: -6·4 (95% CI -7·0, -5·7), -6·0 (95% CI -6·7, -5·4), -6·2 (95% CI -6·8, -5·6), and -5·2 (95% CI -6·0, -4·4) in the mITT population and was similar in the PP population. The least square mean (LSM) difference, 95% confidence interval (CI), and p-values were as follows: -1·1 (95% CI -2·2, -0·1; p=0·029); -0·8 (95% CI -1·9, 0·2; p=0·11), and -1·0 (95% CI -1·9, 0·0; p=0·043) for O. D. 5·0, OD 20·0, and pooled B244, respectively, highlighting a significant difference in response rate between the OD 5·0 and pooled B244 groups compared to vehicle. Additionally, improvements in mean change (LSM) in 5-D Pruritus Scale from baseline to Week 4 for OD 5·0, OD 20·0, pooled B244, and the vehicle group and were respectively: -5·2 (95% CI -5·6, -4·7), -5·2 (95% CI -5·6, -4·7), -5·2 (95% CI -5·5, -4·8), and -4·2 (95% CI -4·7, -3·7) in the mITT population and was similar in the PP population. All B244 treatment groups experienced a significantly larger reduction from baseline compared to the vehicle group. LSM difference, 95% CI, and p-values are as follows: -1·0 (95% CI -1·7, -0·3; p=0·0045); -1·0 (95% CI -1·7, -0·3; p=0·0043), and -1·0 (95% CI -1·6, -0·4; p=0·0021) for O. D. 5·0, OD 20·0, and pooled B244, respectively (Figure 2).

For onset of treatment effect in itch, mean change (LSM) in worst itch numerical rating scale (WI-NRS) from baseline to Week 2 for OD 5·0, OD 20·0, pooled group, and the vehicle group were respectively: -2·0 (95% CI -2·3, -1·7), -2·0 (95% CI -2·2, -1·7), -2·0 (95% CI -2·2, -1·7), and -1·5 (95% CI -1·8, -1·2). LSM difference, 95% CI, and p-values were as follows: -0·5 (95% CI -0·9, -0·1; p=0·025); -0·5 (95% CI -0·9, 0·0; p=0·037), and -0·5 (95% CI -0·9, -0·1; p=0·024) for O. D. 5·0, OD 20·0, and pooled B244, respectively, compared to subjects in the vehicle group. At Week 1, OD 5·0 and pooled B244 groups had a significantly higher proportion of subjects who experienced a ≥4 point improvement in WI-NRS from baseline to Week 1 compared to subjects in the vehicle group (Figure 3). That is, 8% (14/169), 8% (13/171), and 8% (27/340) of subjects in OD 5·0 (OR=3·09; p=0·034), OD 20·0 (OR=2·81; p=0·054), and pooled B244 (OR=2·95; p=0·029), respectively, compared to 3% (5/176) for the vehicle group, experienced this level of improvement.

For the durability of treatment effect, subjects in the B244 treatment groups showed significantly larger reductions in their WI-NRS score from baseline to Week 8 compared to subjects in the vehicle group. Mean change in WI-NRS from baseline to Week 8 for the OD 5·0, OD 20·0, and pooled B244 treatment groups were LSM = -3·1 (95% CI -3·4, -2·8), -3·3 (95% CI -3·6, -3·0), and -3·2 (95% CI -3·4, -2·9), respectively. The decrease from baseline was less for the vehicle group with LSM = -2·4 (95% CI -2·8, -2·1). The LSM difference was -0·6 [p=0·005] for OD 5·0, -0·9 [p=0·0001] for OD 20·0, and -0·8 [p=0·0005] for pooled B244. In addition, subjects in the B244 treatment groups showed significantly larger reductions in their IGA score from baseline to Week 8 compared to subjects in the vehicle group. Mean change in IGA from baseline to Week 8 for the OD 5·0, OD 20·0, and pooled B244 treatment groups were LSM = -0·9 (95% CI -1·0, -0·8), -1·0 (95% CI -1·1, -0·8), and -0·9 (95% CI -1·0, -0·8), respectively. The decrease from baseline was less for the vehicle group with LSM = -0·7 (95% CI -0·8, -0·6). The LSM difference was -0·2 [p=0·013] for OD 5·0, -0·3 [p=0·0032] for OD 20·0, and -0·2 [p=0·0034] for pooled B244. Similar trends were observed across all continuous and categorical endpoints, including EASI.

Table 2 Supplemental

Proportion of Subjects with IGA of Clear or Almost Clear at Week 4 – Primary Analysis (Modified Intent-to-Treat Population)

Visit	Statistic Type	Statistic	B244 OD 5·0 (N=172)	B244 OD 20·0 (N=172)	Pooled B244 (N=344)	Vehicle (N=177)
Week 4	Logistic	n/N (Proportion %) 95% CI [1]	46/166 (28) (21·1, 35·2)	62/164 (38) (30·4, 45·7)	108/330 (33) (27·7, 38·1)	34/171 (20) (14·2, 26·7)
	v. Vehicle	Odds Ratio (95% CI)	1·54 (0·93, 2·56)	2·45 (1·50, 4·00)	1·96 (1·26, 3·04)	N/A
		p-value	0·093	0·0003	0·0027	N/A
	20·0 v 5·0	Odds Ratio (95% CI)	N/A	1·59 (1·00, 2·52)	N/A	N/A
		p-value	N/A	0·051	N/A	N/A

Proportion of Subjects with IGA of Clear or Almost Clear at Week 4, Sub-Analysis (Modified Intent-to-Treat Population)

Visit	Statistic Type	Statistic	B244 OD 5·0 (N=172)	B244 OD 20·0 (N=172)	Pooled B244 (N=344)	Vehicle (N=177)
Week 4	Logistic	n/N (Proportion %) 95% CI [1]	45/157 (29) (21·7, 36·4)	55/150 (37) (29·0, 44·9)	100/307 (33) (27·4, 38·1)	32/161 (20) (14·0, 26·9)
	v. Vehicle	Odds Ratio (95% CI)	1·62 (0·96, 2·72)	2·33 (1·40, 3·89)	1·95 (1·24, 3·07)	N/A
		p-value	0·069	0·0011	0·0041	N/A
	20·0 v 5·0	Odds Ratio (95% CI)	N/A	1·44 (0·89, 2·33)	N/A	N/A
		p-value	N/A	0·14	N/A	N/A

Proportion of Subjects with IGA of Clear or Almost Clear at Week 4 – Non-Responder Imputation (NRI) Analysis (Modified Intent-to-Treat Population)

Visit	Statistic Type	Statistic	B244 OD 5·0 (N=172)	B244 OD 20·0 (N=172)	Pooled B244 (N=344)	Vehicle (N=177)
Week 4	Logistic	n/N (Proportion %) 95% CI [1]	45/167 (27) (20·4, 34·3)	55/164 (34) (26·4, 41·3)	100/331 (30) (25·3, 35·5)	32/171 (19) (13·2, 25·4)
	v. Vehicle	Odds Ratio (95% CI)	1·60 (0·96, 2·68)	2·19 (1·33, 3·62)	1·88 (1·20, 2·95)	N/A
		p-value	0·072	0·0022	0·006	N/A
	20·0 v 5·0	Odds Ratio (95% CI)	N/A	1·37 (0·85, 2·19)	N/A	N/A
		p-value	N/A	0·19	N/A	N/A

Proportion of Subjects with >=90% Improvement in EASI (EASI-90) from Baseline to Week 4 – Primary Analysis (Modified Intent-to-Treat Population)

Visit	Statistic Type	Statistic	B244 OD 5·0 (N=172)	B244 OD 20·0 (N=172)	Pooled B244 (N=344)	Vehicle (N=177)
Week 4	Logistic	n/N (Proportion %) 95% CI [1]	25/166 (15) (10·0, 21·4)	30/164 (18) (12·7, 25·1)	55/330 (17) (12·8, 21·1)	10/171 (6) (2·8, 10·5)
	v. Vehicle	Odds Ratio (95% CI)	2·85 (1·32, 6·14)	3·60 (1·70, 7·64)	3·22 (1·60, 6·49)	N/A
		p-value	0·0074	0·0008	0·0011	N/A
	20·0 v 5·0	Odds Ratio (95% CI)	N/A	1·26 (0·71, 2·26)	N/A	N/A
		p-value	N/A	0·43	N/A	N/A

Proportion of Subjects with >=90% Improvement in EASI (EASI-90) from Baseline to Week 4– Sub-Analysis (Modified Intent-to-Treat Population)

Visit	Statistic Type	Statistic	B244 O.D. 5·0 (N=172)	B244 O.D. 20·0 (N=172)	Pooled B244 (N=344)	Vehicle (N=177)
Week 4	Logistic	n/N (Proportion %) 95% CI [1]	25/157 (16) (10·6, 22·6)	28/150 (19) (12·8, 25·8)	53/307 (17) (13·2, 22·0)	9/161 (6) (2·6, 10·3)
	v. Vehicle	Odds Ratio (95% CI)	3·20 (1·44, 7·10)	3·88 (1·76, 8·52)	3·52 (1·69, 7·35)	N/A
		p-value	0·0042	0·0008	0·0008	N/A
	20·0 v 5·0	Odds Ratio (95% CI)	N/A	1·21 (0·67, 2·19)	N/A	N/A
		p-value	N/A	0·53	N/A	N/A

Proportion of Subjects with >=90% Improvement in EASI (EASI-90) from Baseline to Week 4 – Non-Responder Imputation (NRI) Analysis (Modified Intent-to-Treat Population)

Visit	Statistic Type	Statistic	B244 OD 5·0 (N=172)	B244 OD 20·0 (N=172)	Pooled B244 (N=344)	Vehicle (N=177)
Week 4	Logistic	n/N (Proportion %) 95% CI [1]	25/167 (15) (9·9, 21·3)	28/164 (17) (11·7, 23·7)	53/331 (16) (12·2, 20·4)	9/171 (5) (2·4, 9·8)
	v. Vehicle	Odds Ratio (95% CI)	3·17 (1·43, 7·01)	3·71 (1·69, 8·12)	3·43 (1·65, 7·14)	N/A
		p-value	0·0044	0·0011	0·001	N/A
	20·0 v 5·0	Odds Ratio (95% CI)	N/A	1·17 (0·65, 2·11)	N/A	N/A
		p-value	N/A	0·6	N/A	N/A

Table 2 Supplemental. Efficacy outcomes, baseline to Week 4 of the mITT population

Primary analysis required that at least one WI-NRS rating be reported in a week for the weekly average to be calculated.

mITT=Modified intent to treat. CI=confidence interval. Logistic=Logistic regression model. IGA=Investigator global assessment. OD=Optical density. EASI=Eczema area and severity index. NRI=Non responder imputation.

[1] n is the number of subjects who have improved at that visit. N is the number of subjects with data at that visit.
